# Egg-Derived Anti-SARS-CoV-2 Immunoglobulin Y (IgY) With Broad Variant Activity as Intranasal Prophylaxis Against COVID-19

**DOI:** 10.3389/fimmu.2022.899617

**Published:** 2022-06-01

**Authors:** Lyn R. Frumkin, Michaela Lucas, Curtis L. Scribner, Nastassja Ortega-Heinly, Jayden Rogers, Gang Yin, Trevor J. Hallam, Alice Yam, Kristin Bedard, Rebecca Begley, Courtney A. Cohen, Catherine V. Badger, Shawn A. Abbasi, John M. Dye, Brian McMillan, Michael Wallach, Traci L. Bricker, Astha Joshi, Adrianus C. M. Boon, Suman Pokhrel, Benjamin R. Kraemer, Lucia Lee, Stephen Kargotich, Mahima Agochiya, Tom St. John, Daria Mochly-Rosen

**Affiliations:** ^1^ School of Medicine, SPARK at Stanford, Stanford University, Stanford, CA, United States; ^2^ Faculty of Health and Medical Sciences Internal Medicine, The University of Western Australia, Perth, WA, Australia; ^3^ Independent Regulatory Consultant, Oakland, CA, United States; ^4^ Avian Vaccine Services, Charles River Laboratories, Storrs, CT, United States; ^5^ Linear Clinical Research Ltd, Nedlands, WA, Australia; ^6^ Sutro Biopharma Inc., South San Francisco, CA, United States; ^7^ Virology Division, United States Army Medical Research Institute of Infectious Diseases, Frederick, MD, United States; ^8^ The Geneva Foundation, Tacoma, WA, United States; ^9^ Bravado Pharmaceuticals, Lutz, FL, United States; ^10^ University of Technology Sydney, Sydney, NSW, Australia; ^11^ SPARK Sydney, Sydney, NSW, Australia; ^12^ Department of Medicine, Washington University School of Medicine, St. Louis, MO, United States; ^13^ Department of Chemical and Systems Biology, Stanford University, School of Medicine, Stanford, CA, United States; ^14^ School of Medicine, SPARK Global, Stanford University, Stanford, CA, United States

**Keywords:** immunoglobulin Y, IgY, chicken immunoglobulin, infectious diseases, SARS-CoV-2, COVID-19, clinical trial body

## Abstract

COVID-19 emergency use authorizations and approvals for vaccines were achieved in record time. However, there remains a need to develop additional safe, effective, easy-to-produce, and inexpensive prevention to reduce the risk of acquiring SARS-CoV-2 infection. This need is due to difficulties in vaccine manufacturing and distribution, vaccine hesitancy, and, critically, the increased prevalence of SARS-CoV-2 variants with greater contagiousness or reduced sensitivity to immunity. Antibodies from eggs of hens (immunoglobulin Y; IgY) that were administered the receptor-binding domain (RBD) of the SARS-CoV-2 spike protein were developed for use as nasal drops to capture the virus on the nasal mucosa. Although initially raised against the 2019 novel coronavirus index strain (2019-nCoV), these anti-SARS-CoV-2 RBD IgY surprisingly had indistinguishable enzyme-linked immunosorbent assay binding against variants of concern that have emerged, including Alpha (B.1.1.7), Beta (B.1.351), Delta (B.1.617.2), and Omicron (B.1.1.529). This is different from sera of immunized or convalescent patients. Culture neutralization titers against available Alpha, Beta, and Delta were also indistinguishable from the index SARS-CoV-2 strain. Efforts to develop these IgY for clinical use demonstrated that the intranasal anti-SARS-CoV-2 RBD IgY preparation showed no binding (cross-reactivity) to a variety of human tissues and had an excellent safety profile in rats following 28-day intranasal delivery of the formulated IgY. A double-blind, randomized, placebo-controlled phase 1 study evaluating single-ascending and multiple doses of anti-SARS-CoV-2 RBD IgY administered intranasally for 14 days in 48 healthy adults also demonstrated an excellent safety and tolerability profile, and no evidence of systemic absorption. As these antiviral IgY have broad selectivity against many variants of concern, are fast to produce, and are a low-cost product, their use as prophylaxis to reduce SARS-CoV-2 viral transmission warrants further evaluation.

**Clinical Trial Registration:**

https://www.clinicaltrials.gov/ct2/show/NCT04567810, identifier NCT04567810.

## 1 Introduction

As of May 1, 2022, over 510 million persons with coronavirus disease 2019 (COVID-19) have been identified in 222 countries and territories, resulting in an estimated 6.2 million deaths (1). It is estimated that only 65% of the world population has been fully vaccinated (2), a figure lower than believed needed to reach herd (or community) immunity to stop the pandemic (3, 4). The failure of virus- and vaccine-induced immunity to prevent transmission has complicated this goal (5). Furthermore, only 15% of people in low-income countries have received at least one vaccine dose to date (2).

The fast emergency regulatory authorizations and approvals of COVID-19 vaccines in various countries were a critical turning point in slowing the spread of the pandemic. However, there remains a global need to develop additional safe, effective, easy-to-produce, and inexpensive prophylaxis to prevent or reduce the risk of acquiring severe acute respiratory syndrome coronavirus 2 (SARS-CoV-2) infection (6, 7). This need is due in part to the global shortage of essential components necessary for manufacturing the intramuscular mRNA vaccines and the requirement for cold chain storage for distribution. In addition, novel means of prevention are of heightened importance as variants of SARS-CoV-2 such as Delta (B.1.617.20) and Omicron (B.1.1.529) increase contagiousness and evade immunity produced by existing vaccines and previous infection (8–13) and where COVID-19 vaccination is unavailable, especially in resource-poor settings. These variants have occurred even in populations with high vaccine uptake and are now the most prevalent COVID-19 strains globally (14).

The main entry route for SARS-CoV-2 is the nasal mucosa, which has high levels of the human angiotensin-converting enzyme 2 (hACE2) receptor that is used by the virus to gain cellular entry (15). Viral binding to the hACE2 receptor is mediated by the spike (S) protein on the surface of the viral envelope (16) for all SARS-CoV-2 variants identified so far; even the highly mutated Omicron variant, with 15 mutations in the receptor-binding domain (RBD) of the S protein, is still dependent on hACE2 for its infectivity (11). Therefore, the nasal mucosa is an excellent site as a critical barrier to reduce SARS-CoV-2 entry; antibodies against the SARS-CoV-2 RBD can compete with viral binding to the hACE2 receptor. In addition, antibodies on epithelial surfaces can greatly inhibit lateral viral motility, agglutinate viral particles, and anchor the virus to the extracellular matrix (17, 18), thus making intranasally administered antibodies a potentially important antiviral strategy. Indeed, intranasal antibody prophylaxis has been shown to be effective against multiple viral pathogens in humans and veterinary applications, including respiratory tract viruses (18). Thus, covering the nasal mucosa with anti-SARS-CoV-2 antibodies could prevent SARS-CoV-2 infection in naïve individuals and may also reduce viral transmission from an infected individual by reducing levels of active virus.

Because the RBD remains essential for SARS-CoV-2 infection, even for variants of concern, we chose recombinant RBD of the S protein (amino acids 328-533) as the immunogen. We next considered the optimal species to raise anti-SARS-CoV-2 polyclonal antibodies and chose to immunize egg-laying hens to enable fast, low-cost, and high-volume production. Antibodies generated in commercial hens (immunoglobulin Y; IgY) are concentrated in their eggs to 50-100 mg/egg (thus yielding approximately 35 g of IgY per year) within 2-3 weeks following vaccination (19–21). This yield can be up to five times higher (175 g of IgY in one year) when using specific-pathogen-free (SPF) hens.

Here, we describe the production of anti-SARS-CoV-2 RBD IgY of the S protein in SPF hens and the characterization of these IgY, including in culture neutralization efficacy against current pathogenic viral variants and a phase 1 study that evaluated the safety, tolerability, and pharmacokinetics of anti-SARS-CoV-2 RBD IgY given by intranasal drops in healthy humans.

## 2 Materials and Methods

### 2.1 Study Design

The experiments were conducted in four parts: 1) production of immunogen (recombinant RBD), immunization of 12 SPF hens, IgY collection from egg yolks, and *in vitro* characterization of the IgY anti-SARS CoV-2 RBD, 2) Good Laboratory Practice (GLP)-blinded safety studies in rat, treated intranasally twice daily for 28 days with a total of 16 mg/kg IgY or vehicle, 3) a preliminary efficacy study of hamsters treated with IgY or phosphate-buffered saline for 4 hours before viral challenge, and 4) a placebo-controlled, double-blind phase 1 safety, tolerability, and pharmacokinetic (PK) study conducted in healthy humans using intranasal IgY or vehicle in single-ascending doses followed by multiple doses (3-times daily every 4 hours) for 14 days. Both the single-ascending and multiple-dose parts were followed by a 7-day nontreatment period to further evaluate safety.

### 2.2 Recombinant SARS-CoV-2 RBD and Characterization

SARS-CoV-2 RBD (residues 328-533) of the 2019 novel coronavirus index virus (2019-nCoV) was expressed in cell-free protein synthesis reactions at Sutro Biopharma, Inc. (South San Francisco, CA) using the XpressCFTM platform (22, 23). Binding kinetics of cell-free expressed SARS-CoV-2 RBD construct or mammalian expressed his-tagged RBD control (ACROBiosystems SPD-C52H1) were then measured on a Biacore T200 instrument, using Fc-tagged hACE2 receptor protein (ACROBiosystems AC2-H5257); see **Supplementary Methods**.

### 2.3 Hen Immunization and IgY Purification and Characterization

Nine SPF were hens immunized with an inoculum containing 50 µg of recombinant cell-free expressed RBD fragment derived from S1 spike protein and water-in-oil adjuvant, boosted after 14 days, and then boosted again 4 weeks later, unless otherwise indicated. Nonfertilized eggs were collected weekly. Yolks in batches up to 100 eggs/batch were separated and IgY was purified using water extraction, an established method to purify IgY (24–27). Hen sera and purified IgY were tested by enzyme-linked immunosorbent assay (ELISA) and Western blot analysis using both the immunogen (RBD) and full length glycosylated S1 protein (ACROBiosystems, cat# S1N-C5255). See **Supplementary Methods** for more details.

### 2.4 Evaluation of IgY

#### 2.4.1 ELISA Evaluation of IgY Titer Against SARS-CoV-2 Variants of Concern

ELISA titer of the final IgY preparation used in the clinical studies against the Alpha, Beta, Delta, and Omicron-derived RBD [amino acids 319-537; ACROBiosystems SPD-C52H1(index), SPD-C52Hn (Alpha), SPD-C52Hp (Beta), SPD-C525e (Delta), and SPD-C522e (Omicron)] was carried out as described in **Supplementary Methods**.

#### 2.4.2 In Culture Viral Neutralization Studies Using Pseudovirus

The neutralization assays using pseudovirus were performed at RetroVirox (San Diego, CA). The assay used three non-replicative vesicular stomatitis virus (VSV) pseudoviruses carrying a firefly luciferase reporter gene and expressing the S proteins of the index SARS-CoV-2 Hu-1 spike, a truncated spike with a C-terminal 19 amino acid deletion; the Beta variant (K417N/E484K/N501Y/D614G full-length spike); or a D614G spike variant with a full-length sequence of the index spike protein. The neutralization assay was performed with HEK 293T-hACE2, a human embryonic kidney cell line overexpressing hACE2. For details, see **Supplementary Methods**.

#### 2.4.3 In Culture Neutralization Studies Using Live Virus

At the United States Army Medical Research Institute of Infectious Diseases (USAMARIID; Frederick, MD), all work with authentic (live) SARS-CoV-2 (SARS-CoV-2/B.1.617.2) studies were conducted in Biosafety Level 3 laboratories following federal and institutional biosafety standards and regulations. Vero-76 cells were inoculated for 1 hour with SARS-CoV-2/Was1 (MT020880.1) after a 1 hour preincubation of the virus with control or anti SARS-CoV-2 IgY antibodies. At 23 hours after infection, cells were washed, fixed in 10% formalin, and permeabilized with 0.2% Triton-X for 10 minutes. Detection of infection was accomplished using an anti-SARS-CoV-2 nucleocapsid protein detection antibody (Sino Biological).

At RetroVirox (San Diego, CA), purified IgY (lot Y0180) was also tested against three live SARS-CoV-2 clinical isolates: MEX-BC15/2021 (lineage B.1.617.2, Delta), USA/SD-RVX01/2022 (lineage B.1.529, Omicron) and MEX-BC2/2020 (lineage B.1, carrying the D614G mutation) by infecting Vero E6 cells in the presence or absence of test items. Virus-induced cytopathic effect was monitored under the microscope after 3 days of infection. Some cells were treated with plasma from an uninfected individual who received two doses of Moderna’s mRNA COVID-19 vaccine as a positive control. For more details, see **Supplementary Methods**.

### 2.5 GMP IgY Formulation, Analytical Studies, and Stability

Using Good Manufacturing Practice (GMP), anti-S1 RBD IgY was formulated for use as intranasal drops as 0 (placebo control), 5, 10, and 20 mg/mL anti-S1 RBD IgY preparations in sterile 2% microcrystalline cellulose and carboxymethylcellulose sodium at Bravado Pharmaceuticals (Lutz, FL). The suspension was packed in a 1.5-mL dropper bottle and tests for GMP drug product release complied with the relevant standards and methods, including microbiological examination of nonsterile products (USP <1111>, <61> and <62>). All formulated products were 100% stable as measured by physical and analytical properties, including high-performance liquid chromatography (HPLC), when stored for at least 6 months at 2-8°C and about 1 month when stored at room temperature.

### 2.6 GLP Rat Toxicity and Safety Study

Thirty-five female and 35 male >8-week-old Sprague Dawley rats were used in a GLP study conducted at Charles River Laboratories (Spencerville, OH). Water and food were freely available. The experimental protocol is summarized in **Supplementary Tables S1-S3**.

#### 2.6.1 Cytokine Level Measurement and Analysis

This non-GLP blinded assay was performed on serum by the Immunoassay Team at the Human Immune Monitoring Center at Stanford University (Stanford, CA). Assay kits (RECYMAG65K27PMX Rat) were purchased from EMD Millipore and used according to the manufacturer’s recommendations, with modifications described in **Supplementary Methods**.

#### 2.6.2 IgY in Sera of Treated Rats

The presence of anti-SARS-CoV-2 IgY in sera of rats was evaluated using GLP standards in a qualified ELISA assay at Charles River Laboratories (Reno, NV). See **Supplementary Methods** for more details.

### 2.7 Human Tissue Cross-Reactivity Study

A GLP study examining human tissue reactivity of the anti-SARS-CoV-2 RBD IgY was conducted at Charles River Laboratories (Frederick, MD) using at least 3 tissues from at least 3 donors (**Supplementary Table S4**), using 10 mg/mL or 20 mg/mL IgY control (negative control) and anti-SARS-CoV-2 RBD IgY or an anti-human hypercalcemia of malignancy peptide (amino acid residues 1-34, Sigma-Aldrich; Catalog No. H9148; positive control), each in 1% bovine serum albumin. The slides were then visualized by light microscopy.

### 2.8 Virus Preparation for the Efficacy Study in a Hamster Model of COVID-19 and Quantitation of Viral Load in the Animals

Syrian hamsters develop mild-to-moderate disease with progressive weight loss that starts several days after SARS-CoV-2 infection by intranasal inoculation (28, 29). SARS-CoV-2 (strain 2019-nCoV/USA-WA1/2020) was propagated on Vero-TMPRSS2 cells, and the virus titer was determined by plaque assays on Vero-hACE2 and Vero-hACE2-TMPRSS2 cells. Five- to 6-week-old Syrian golden hamsters (Charles River Laboratories) infected with SARS-CoV-2 as previously described (29) were housed at the Washington University (St. Louis, MO) Biosafety Level 3 facility in HEPA-filtered rodent cages. Before challenge with SARS-CoV-2, all animals received 100 μL of a placebo control fluid or fluid-formulated anti-SARS-CoV-2 RBD IgY (1 mg/50 mL of the 20 mg/mL solution per nare). Four hours after the delivery, the animals were challenged with 10^4^ or 5 x 10^4^ plaque-forming units (PFU) of SARS-CoV-2 (titer determined on Vero-hACE2; see below).

The protocol included 18 hamsters in 4 planned groups: Group 1: Control with 5 x 10^4^ PFU (4 animals); Group 2: Control with 1 x 10^4^ PFU (4 animals); Group 3: Anti-SARS-CoV-2 RBD IgY with 5 x 10^4^ PFU (5 animals); and Group 4: Anti-SARS-CoV-2 RBD IgY with 1 x 10^4^ PFU (5 animals). Note that the viral titer in Vero-hACE2-hTMPRSS2 cells, which express the essential protease to liberate the RBD from the S protein on the surface of the virus (30), was subsequently found to be almost 100-fold higher for Groups 1 and 3 (4 x 10^6^ PFU) and Groups 2 and 4 (0.8 x 10^6^ PFU). Three days after challenge, the animals were sacrificed and lungs were collected. See **Supplementary Methods**.

### 2.9 Phase 1 Safety, Tolerability, and Pharmacokinetic Study in Humans

#### 2.9.1 Study Design and Dose Selection

**Figure 1:**
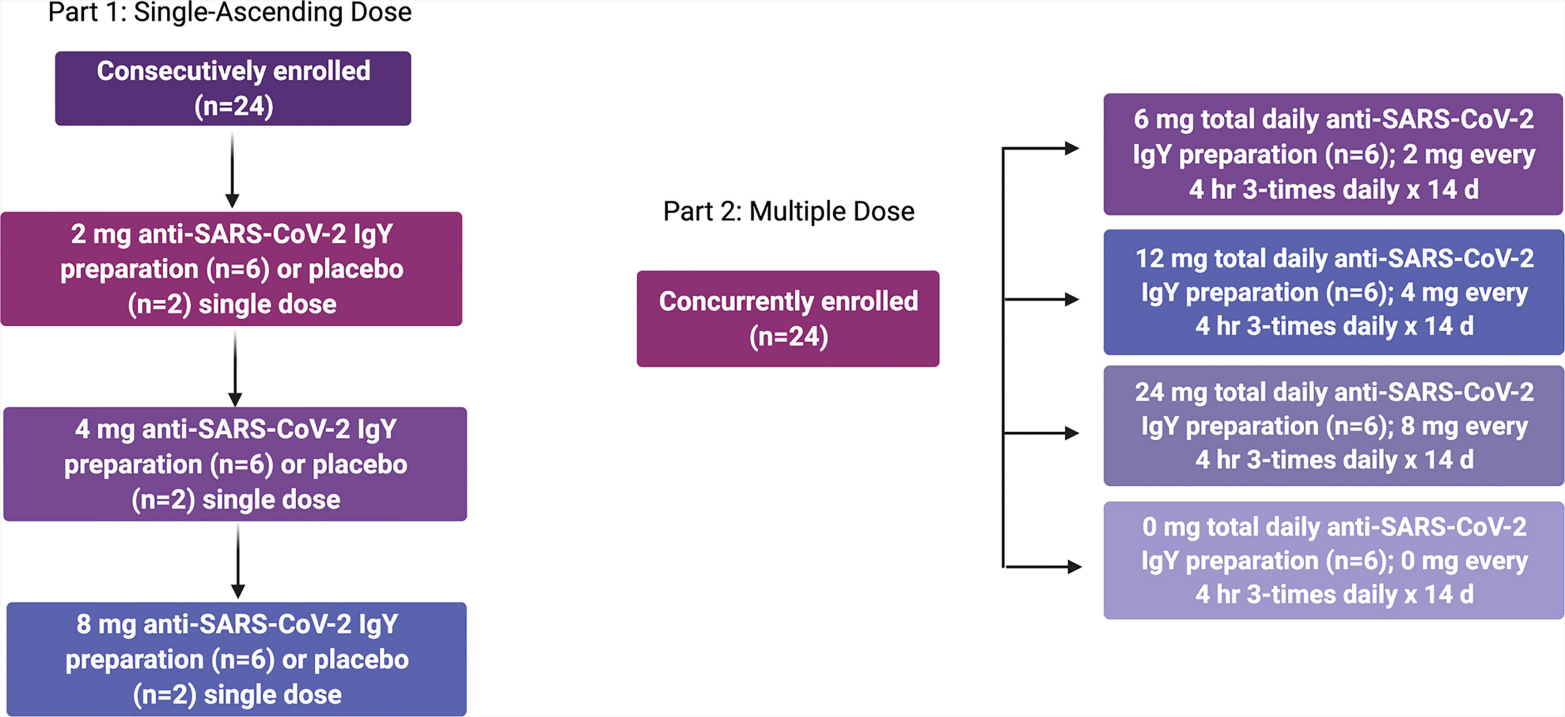
Design of phase 1 human single-ascending and multiple-dose study.

A single-center, randomized, double-blind, placebo-controlled phase 1 study of anti-SARS-CoV-2 RBD IgY given intranasally to 48 healthy adults was conducted at Linear Clinical Research-Harry Perkins Research Institute (Nedlans, WA, Australia) (see **Figure 1**). Written informed consent was obtained from all participants. The study is registered at ClinicalTrials.gov: NCT04567810. The study period was conducted between September 25, 2020 and December 14, 2020. The protocol for this trial is available as supporting information (**Supplementary Methods** **)**.

Healthy male and female participants ≥18 and ≤ 45 years old with a body weight ≥ 50 kg and a body mass index ≥ 18.0 and ≤ 32.0 kg/m^2^ were eligible for this study. Females of childbearing potential who were pregnant or lactating or planning to become pregnant during the study and participants with a history of alcohol and drug abuse, current smoking, clinically significant laboratory abnormalities, history of nasal surgical procedures, frequent or recurrent nasal conditions, current use of any nasal preparations, evidence of or history of clinically significant conditions, or positive test for hepatitis B, hepatitis C, human immunodeficiency virus, or SARS-CoV-2 nucleic acid or serology were excluded from participation. Full eligibility criteria are summarized in **Supplementary Methods**.

The master randomization schedule and the associated code break envelope files were produced by an unblinded statistician using a computer-generated (SAS^®^ v9.4 PLAN procedure) pseudo-random permutation procedure. For Part 1, the first two randomization numbers for each cohort were randomly assigned in a 1:1 ratio (anti-SARS-CoV-2 IgY: Placebo) to allow for sentinel dosing, and the remainder of the numbers for each cohort was generated in a 5:1 (anti-SARS-CoV-2 IgY: Placebo) ratio using a permuted blocked randomization with a block size of six. For Part 2, 24 numbers were generated in a 1:1:1:1 ratio (6 mg anti-SARS-CoV-2 IgY: 12 mg anti-SARS-CoV-2 IgY: 24 mg anti-SARS-CoV-2 IgY: Placebo) using a permuted blocked randomization with a block size of six. The block sizes were kept confidential during the study.

The site personnel randomized eligible participants on Day 1 by assigning the next available randomization number for the specific study part to the participant and reporting the randomization number on the case report form. Study drug was prepared by an unblinded pharmacist based on the treatment corresponding to the assigned randomization number on the randomization schedule that was only available to the pharmacist. In the event of an emergency, authorized personnel were able to unblind a participant through the code break envelope associated with the randomization number assigned to the participant.

In Part 1, participants were randomly assigned to receive a single dose of anti-SARS-CoV-2 RBD IgY antibodies or placebo in a sequential escalating manner. Three groups were sequentially dosed with 8 healthy participants per group (6 active and 2 placebo in each group). Each group in Part 1 included the initial dosing of a sentinel group (1 anti-SARS-CoV-2 RBD IgY and 1 placebo) at least 24 hours before dosing the remaining 6 participants in the cohort (5 anti-SARS-CoV-2 RBD IgY and 1 placebo). The remainder of the cohort were dosed if, in the opinion of the investigator, there were no significant safety concerns identified in the sentinel participants within the first 24 hours after administration of the dose (anti-SARS-CoV-2 RBD IgY or placebo). A Safety Monitoring Committee (SMC) reviewed safety data before each dose escalation. The following regimens were administered: 2 mg anti-SARS-CoV-2 RBD IgY preparation or placebo, 4 mg anti-SARS-CoV-2 RBD IgY preparation or placebo, and 8 mg anti-SARS-CoV-2 RBD IgY preparation or placebo. In Part 1, 2 drops were applied to each nostril as a single administration. A 7-day nontreatment follow-up period assessed safety after completion of the dosing.

In Part 2, participants were randomly assigned to receive multiple daily administrations of anti-SARS-CoV-2 RBD IgY or placebo every 4 hours (3-times daily) for 14 days in a parallel-group manner. Up to 24 healthy participants were randomized to 1 of 4 treatment regimens (6 participants per regimen). The following regimens were administered: 6 mg total daily dose anti-SARS-CoV-2 RBD IgY preparation for 14 days, 12 mg total daily dose anti-SARS-CoV-2 RBD IgY preparation for 14 days, 24 mg total daily dose anti-SARS-CoV-2 RBD IgY preparation for 14 days, and 0 mg total daily dose placebo preparation for 14 days. In Part 2, 2 drops were applied to each nostril every 4 hours (3-times daily). A 7-day nontreatment follow-up period assessed safety after completion of the dosing.

In each part, 3 groups with 8 healthy participants per group (6 active and 2 placebo in each group) were dosed. Safety and tolerability were evaluated using adverse event, physical examination (including vital signs), electrocardiogram, and clinical laboratory data. PK of anti-SARS-CoV-2 RBD IgY was evaluated by measuring serum concentrations pretreatment and at Day 14 when given as multiple doses administered intranasally for 14 days.

The investigational drug was supplied as a liquid preparation in a nose drop bottle containing 1.5 mL anti-SARS-CoV-2 RBD IgY preparation nasal suspension at 5, 10, or 20 mg/mL, or placebo, for intranasal application. Each bottle of nasal drops had enough material for one day of use. The liquid preparation contained anti-SARS-CoV-2 RBD IgY 0.5 mg/100 µL/drop, 1 mg/100 µL/drop, or 2 mg/100 µL/drop. The total maximum daily dose of anti-SARS-CoV-2 RBD IgY used in the present study (24 mg) was based in part on solubility considerations and is less than the daily dose of anti- *Pseudomonas aeruginosa* IgY previously given prophylactically as an oral (mouth rinse) treatment to prevent pulmonary infections in 17 patients with cystic fibrosis (31). For the maximum anti-SARS-CoV-2 RBD IgY dose of 4 mg/nare, we calculated a favorable ratio of IgY to viral particles, even when virus covers the nasal pathway.

All participants were provided with a Dose Administration Guide and instructed to “Gently blow your nose before using this drug. Then tilt your head back while sitting or lying down. After the study drug is administered, keep your head tilted for a few minutes. Try not to blow your nose for at least 5 minutes after study drug administration.”

#### 2.9.2 Assessments

Safety (and tolerability) were evaluated using adverse event, physical examination (including vital signs), electrocardiogram, and clinical laboratory data that included nonfasted collection of hematology, serum metabolic panel, coagulation, urinalysis, and urine human chorionic gonadotropin values. Pharmacokinetics following intranasal administration of anti-SARS-CoV-2 RBD IgY were evaluated in the multiple-dose part of the study by measuring serum anti-SARS-CoV-2 RBD IgY concentration (lower limit of quantification, 30 ng/mL) at baseline and 2 hours after final dosing on Day 14, as described above (Charles River Laboratories, Reno, NV).

Serum cytokine levels for exploratory analyses were evaluated from the sera of 19 multiple-dose participants before and 2 hours after dosing on Days 1 and 2, as described above (Stanford Human Immune Monitoring Center, Stanford CA). An exploratory analysis was conducted of pretreatment serum total immunoglobulin E (IgE) and anti-IgE antibody (mainly anti-ovalbumin) levels in the 24 participants in the multiple-dose part of the trial.

#### 2.9.3 Changes in the Conduct of the Study

All participants were enrolled, treated, and assessed under Protocol CVR001 version 3.0, dated 29 September 2020 (**Supplementary Methods** **)**. The following changes were made to the conduct of the study from what was specified in the protocol:

- Participants were reconsented to allow for exploratory cytokine analyses on stored blood samples.
- Per the study protocol, serum anti-SARS-CoV-2 IgY samples were obtained from participants in the multiple-dose part of the study before dosing and at 0.5, 1, 1.5, and 2 hours after dosing on Days 1 and 14, as well as before dosing and 2 hours after dosing on Days 2, 3, and 4. Because anti-SARS-CoV-2 IgY was not measurable at the time of the theoretical maximum serum concentration at 2 hours postdose on Day 14, the remaining postbaseline PK samples were not analyzed.

#### 2.9.4 Interim Analyses

Before dose escalation in the single-ascending dose cohorts, the SMC was to review all available safety and tolerability data for a minimum of 7 participants who completed the planned safety assessments up to 48 hours after dosing. The SMC comprised three physician members (the principal investigator, sponsor medical representative, and independent medical monitor). The data was to be reviewed blinded, unless the SMC considered it necessary to unblind the data for safety concerns. Before breaking the code per standard procedures, the potential decisions and actions were to be determined. SMC decisions on dose escalation were to be taken in consensus between the members of the SMC. The SMC decisions and their rationale were documented.

#### 2.9.5 Analyses

No formal sample size calculations were done. Based on experience from previous similar studies, the target number of participants was appropriate for the assessment of safety, tolerability, and PK. The planned sample size was 48 participants. A total of 48 participants were enrolled and included in the safety analyses. The analysis of safety variables included all participants who received study drug. All variables were summarized by descriptive statistics for each treatment group. The statistics for continuous variables included mean, median, standard deviation, and number of observations. Categorical variables were tabulated using frequencies and percentages. The incidence of all reported treatment-emergent adverse events and treatment-related adverse events was tabulated by treatment group. Adverse events were also classified by system organ class and preferred term using the Medical Dictionary for Regulatory Activities. Adverse events were to be listed and summarized by treatment group, preferred term, severity, seriousness, and relationship to study drug. In the event of multiple occurrences of the same adverse event with the same preferred term in one participant, the adverse event was counted once as the worst occurrence. Summary statistics for actual values and change from baseline were analyzed for laboratory results by treatment group and scheduled visit. Data summarized by treatment included adverse events, vital signs, electrocardiogram parameters, and clinical laboratory evaluations.

#### 2.9.6 Cytokine Measurement and Analysis

This non-GLP blinded assay was performed on the sera of study participants by the Immunoassay Team at the Human Immune Monitoring Center at Stanford University (Stanford, CA), as described for the rat cytokine assay above. The levels of 80 different cytokines were determined.

#### 2.9.7 GLP Bioanalytical Analysis of IgY in Blood Samples – Toxicokinetic Analysis

The presence of anti-SARS-CoV-2 IgY in sera of study participants was evaluated using GLP standards in a qualified ELISA assay at Charles River Laboratories (Reno, NV) described in **Supplemental Methods**.

### 2.10 Good Laboratory Practice

The study was performed at Charles River Laboratories following the U.S. Department of Health and Human Services, Food and Drug Administration (FDA), United States Code of Federal Regulations, Title 21, Part 58: Good Laboratory Practice for Nonclinical Laboratory Studies and as accepted by Regulatory Authorities throughout the European Union (OECD Principles of Good Laboratory Practice), Japan (MHLW), and other countries that are signatories to the OECD Mutual Acceptance of Data Agreement.

### 2.11 Animal Welfare Assurance and Standards

The protocols and any amendment(s) or procedures involving the care and use of animals (hen immunization and rat tolerability studies) were reviewed and approved by Charles River Laboratories Institutional Animal Care and Use Committee before conduct. The hamster efficacy study was conducted following the recommendations in the Guide for the Care and Use of Laboratory Animals of the National Institutes of Health and the protocol was approved by the Institutional Animal Care and Use Committee at the Washington University School of Medicine (assurance number A3381–01). Stanford Institutional Animal Care and Use Committee did not initially review the animal studies because they were not conducted at Stanford but were retroactively approved.

### 2.12 Regulatory and Ethics Considerations

Ethical review of the clinical trial protocol and any amendments was obtained by Bellberry Human Research Ethics Committee (Australian equivalent to U.S. Institutional Review Board) and the clinical trial was conducted solely at a single investigative site, Linear Clinical Research-Harry Perkins Research Institute (Nedlands, Australia). Stanford Institutional Review Board did not review the research. The study was conducted following the protocol and ethical principles stated in the 2013 version of the Declaration of Helsinki and the applicable guidelines on Good Clinical Practice, and all applicable federal, state, and local laws, rules, and regulations.

## 3 Results

### 3.1 Antigen Production

**Figure 2:**
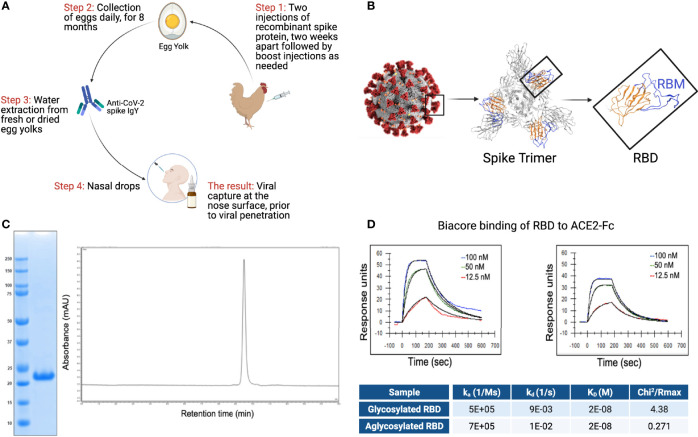
RBD and IgY preparation. **(A)** Workflow of the study. IgY preparation for intranasal drops as antiviral prophylaxis. **(B)** Cell-free expressed RBD derived from the Spike protein on the viral envelope of SARS-CoV-2. **(C)** Characterization of the recombinant protein RBD by ELISA and HPLC. **(D)** Determination of the affinity of the cell-free expressed RBD (amino acids 328-533) and mammalian-expressed full-length S1 to the hACE2 using Biacore.

The overall scheme describing the production of anti-SARS CoV-2 IgY antibody to be used as intranasal prophylaxis in humans is shown in **Figure 2A**. We produced a recombinant protein to immunize hens. A tagless RBD of SARS-CoV-2 (**Figure 2B**; index SARS-CoV-2 variant), amino acids 328-533, was produced in a cell-free protein synthesis reaction using *E.coli* extract (19, 20, 29). Analysis of the purified CoV-2 RBD protein yielded a single protein band with an apparent molecular weight of 23 kDa (**Figure 2C**). The purified SARS-CoV-2 RBD was eluted as a single peak by analytical size-exclusion chromatography with >95% monomer content (**Figure 2C**). Bacterial endotoxin contamination was determined to be <0.1 EU/mg by Charles River Endosafe LAL cartridge system.

Integrity of the cell-free (non-glycosylated) SARS-CoV-2 RBD was then verified by kinetic binding to the hACE2 receptor. Binding kinetics and affinity were similar to a mammalian expressed and glycosylated S1 fragment (**Figure 2D**) and were consistent with previously described binding affinities, suggesting the RBD expressed cell-free was properly folded and bioactive.

### 3.2 Hen Immunization With SARS-CoV-2 RBD and IgY Characterization *In Vitro*

**Figure 3:**
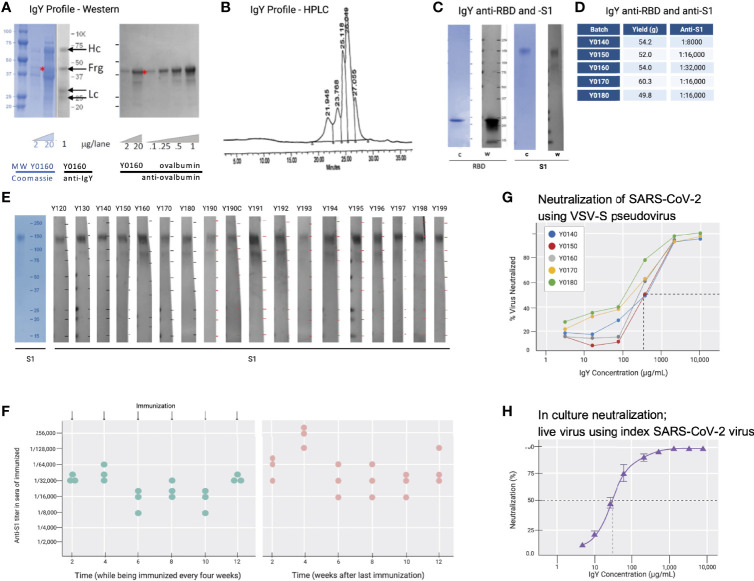
IgY purification and characterization. **(A)** Western blot analysis of the IgY preparation. **(B)** HPLC profile of the IgY preparation. **(C)** Western blot analysis of anti-SARS-CoV-2 IgY against RBD fragment and full S1 recombinant protein. **(D)** IgY yield for various batches derived from 100 eggs each. **(E)** Western blot data of different lots of anti-SARS-CoV-2 RBD IgY (Y0120-Y0199). Pools of 100 eggs laid by 9 hens over 2 weeks were used for each pool of IgY preparation between May 2020 and March 2021. IgY lot samples were diluted 1:500 followed by a 1:3000 dilution of rabbit anti-IgY HRP conjugate. First left lane shows the Coomassie stain of the same gels. **(F)** Time-dependent ELISA titers of sera from 3 individual hens following continual immunization (left); arrows indicate immunization timing. Time-dependent ELISA titer of 3 hens after immunization was stopped for up to 12 weeks (right). **(G)** Neutralization of pseudovirus SARS-CoV-2 by various lots of anti-SARS-CoV-2 RBD IgY (conducted at RetroVirox). **(H)** Neutralization of live index SARS-CoV-2 virus by anti-SARS-CoV-2 RBD IgY (Y0180, conducted at USAMRIID).

Cell-free expressed RBD (**Figure 2B**; 50 µg in simple oil emulsion) was injected into 9 SPF hens (46-weeks old) and IgY was extracted from egg yolks using a water-based method 2 weeks after the second immunization and thereafter. The IgY preparation was subjected to protein and Western blot analyses (**Figure 3A**). The IgY preparations were >95% pure; a quantitative Western blot analysis demonstrated that this preparation contained less than 2% ovalbumin by weight (**Figure 3A**). Chromatography of the IgY preparations on size-exclusion HPLC identified 5 peaks (**Figure 3B**); SDS-PAGE and Western blot analysis of the peaks collected between 17 and 30 minutes confirmed that these peaks all contain IgY. The anti-SARS-CoV-2 RBD IgY antibodies recognized both the immunogen, cell-free expressed RBD, and the mammalian-expressed full-length and glycosylated S1 protein (**Figure 3C**). One egg yolk of the SPF hens provided about 500 mg of purified IgY and each of >10 independent batches of IgY, purified from 100 eggs, each yielded an average of 47 ± 13 g (SD) of purified IgY (see **Figure 3D** for select examples). There was limited variability in the affinity of the various IgY batches for the S glycosylated protein as judged by ELISA (**Figure 3D**; average titer against full-length S1 was 1:18,000). Furthermore, there was almost no difference in titer of individual hens towards the full-length glycosylated S protein, suggesting minimal variability between hens (**Figures 3E, F** **)**. Over 11 months, IgY was collected in batches of 100 eggs per preparation with a similar yield of IgY per preparation and a similar response; interruption of immunizations for 3 months did not result in a drop in titer (**Figure 3F**, right panel).

### 3.3 Neutralization of SARS-CoV-2 Index Strain and Variants of Interest and Concern With Anti-SARS-CoV-2 RBD IgY

Pseudoviruses are synthetic chimeras that consist of a surrogate viral core derived from a parent virus and an envelope glycoprotein derived from a heterologous virus (30). Viral neutralization assays in culture (RetroVirox) used non-replicative VSV pseudoviruses carrying a firefly luciferase reporter gene and expressing S of SARS-CoV-2 on the surface of the virion (VSV-S). The neutralization assay was performed with HEK 293T-hACE2, a human embryonic kidney cell line overexpressing hACE2, the receptor of the SARS-CoV-2 virus. First, 5 batches of RBD IgY preparations were tested. The neutralization activity of the purified IgY, defined as the concentration inhibiting 50% of the viruses (IC_50_) was ~170 µg/mL (**Figure 3G**). Importantly, ~10-fold higher neutralization activity towards the index SARS-CoV-2 virus was observed when using a live index virus (**Figure 3H**). Approximately 30 µg/mL IgY provided 50% neutralization of the index virus in culture (**Figure 3H**).

**Figure 4:**
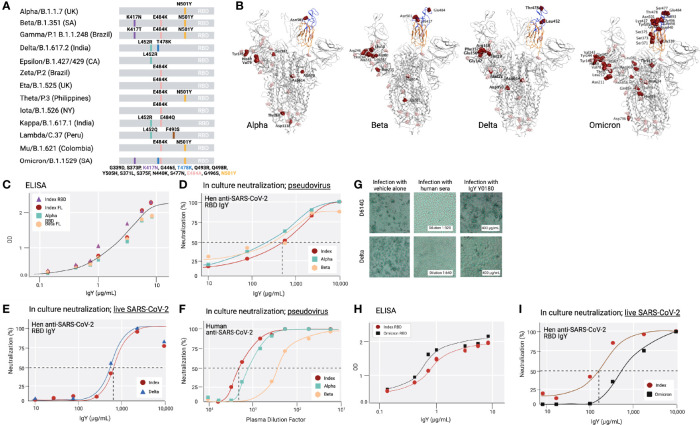
Common SARS-CoV-2 variants and anti-SARS-CoV-2 IgY interaction with them **(A)** A scheme depicting locations of mutated amino acids in Alpha through Mu variants of SARS-CoV-2, focusing on the RBD domain only. Each color bar indicates the amino acid in the index virus that was mutated in the variant. **(B)** Spike protein of SARS-CoV-2 Alpha, Beta, Delta, and Omicron are shown from left to right. Molecular Operating Environment was used to create the figure (35). The location of mutations in the structure of the S protein trimer of SARS-CoV-2 (PDB ID: 7A98) for 4 of the common variants are indicated in red and glycosylation sites are indicated in pink throughout the S protein. Blue ribbon indicates RBD (amino acids 328-533), and the orange ribbon indicates receptor binding motif (amino acids 437-508). **(C)** Binding of anti-SARS-CoV-2 RBD IgY to recombinant S1 full length (FL) of the index virus, the RBD of the Alpha and Beta variants, and the immunizing RBD of the index virus by ELISA. **(D)** Neutralization of pseudovirus (VSV-S) SARS-CoV-2 carrying S protein of index virus, Alpha, or Beta variants by anti-RBD IgY. **(E)** Neutralization of live index or Delta viruses by anti-SARS-CoV-2 IgY against the RBD. **(F)** Neutralization of pseudoviruses listed in **(D)** by human serum of immunized individuals. **(G)** Neutralization of live D614G *vs*. Delta variants by human serum of immunized individual or by anti-SARS CoV-2 IgY. Microscopic evaluation of monolayers of Vero E6 cells after 96 hours infection with the indicated authentic (live) SARS-CoV-2 variant. Images from infected cells are shown after 4 days of infection with SARS-CoV-2 variants in the absence or presence of test items. Top three panels: Infection in the presence of MEX-BC2/2020 and bottom three panels: infection with the Delta variant each in the presence of vehicle alone, serum of a person immunized twice with the Moderna (mRNA-1273) vaccine or anti-SARS CoV-2 RBD IgY, as indicated, all at the indicated concentration (neutralization experiments in panels D-H & I were conducted by RetroVirox using pseudovirus or live virus, as indicated). **(H)** Binding of anti-SARS-CoV-2 RBD IgY to the index virus and Omicron variant (B.1.1.529) RBD domain using ELISA. **(I)** Neutralization of live index or Omicron variant of SARS-CoV-2 by anti-SARS-CoV-2 RBD IgY. Except when indicated, the studies were done over several months; therefore, the absolute titers in the ELISA and neutralization studies were not identical. However, each experiment included the same positive control; index RBD for ELISA and index virus for neutralization assays.

Because at least 13 common variants of SARS-CoV-2 with amino acid mutations in the RBD had emerged since December 2020 (32–34) (**Figure 4A**), we tested the activity of the anti-SARS-CoV-2 IgY against several variants (including Beta, Delta, and Omicron; **Figures 4A, B** **)** and D614G, an amino acid substitution outside the RBD that is now found in most variants. Beta, Delta, and Omicron were classified as variants of concern, associated with increased transmissibility or detrimental change in COVID-19 epidemiology, or an increase in virulence or change in clinical disease presentation.

First, IgY antibody ELISA titer against Beta RBD was compared with the RBD of the index SARS-CoV-2 virus as well as the most common Alpha variant. ELISA with recombinant full-length S protein or the RBD of the 3 mutants as well as the immunizing RBD fragment of the index virus yielded a virtually identical titer (**Figure 4C**). Although the Omicron variant still uses the hACE2 receptor to infect human cells (11, 36), the RBD contains a total of 15 mutations compared to the index virus, 11 of which were not found in the previous variants (**Figures 4A, B** **)**. Yet, the ELISA titer of IgY against the Omicron RBD was also equivalent or slightly better than that towards the RBD of the index virus (**Figure 4H**).

Next, we tested the neutralization titer of anti-SARS-CoV-2 RBD IgY (lot Y0180) against the RBD of the index, Alpha, and Beta variants, thus including all the amino acid substitutions within the RBD also found in the RBD of Gamma, Zeta, Eta, Theta, Iota, and Mu variants and 1 of the 2 substitutions in the Kappa variant (**Figure 4A**). The IC_50_ of the VSV-S pseudovirions for the index strain and Beta variant were virtually identical: 668 μg/mL for the index strain, 568 μg/mL for Beta (**Figure 4D**), and 2-fold lower for Alpha (IC_50_ = 302 μg/mL; **Figure 4D**). RetroVirox also provided data using plasma from a single Moderna-vaccinated individual for a titer comparison of neutralization with anti-SARS-CoV-2 RBD IgY, tested with 3 of the variants. In a side-by-side study, the IC_50_ generated with the human plasma from a recipient of the mRNA Moderna vaccine (2 doses; index SARS-CoV-2) showed the highest titer against the index virus, followed by a 2.8-fold drop in titer towards Alpha and a 6.7-fold lower titer for Beta (**Figure 4F**).

The anti-SARS-CoV-2 IgY preparation was similarly effective against the Delta variant compared with the index strain (**Figure 4E**). The assays show that Y0180 displays similar neutralizing activity against both isolates, with slightly reduced neutralizing activity against Omicron (**Figures 4E, I** **)**; IC_50_ values generated were 635 μg/mL (Delta) and 739 μg/mL (index virus). The neutralization assay against Omicron generated IC_50_ values of 143 μg/mL (index) and 785 μg/mL (Omicron) (**Figure 4I**). Plasma from one Moderna-vaccinated individual was also tested in parallel with both isolates. NT_50_ values generated with this plasma against the variants were 1:1274 (Delta pseudovirus) and 1:1091 (index pseudovirus). The neutralization activities of the antibody and control plasma against both variants were also confirmed by microscopy evaluating the virus-induced cytopathic effect in infected cell monolayers **Figure 4G**). The human serum had a similar degree of neutralization against the index pseudovirus and Delta (**Figure 4G**) when tested at a dose that is three times higher than that required for 50% neutralization (1:640 in **Figure 4G**, middle panels, *vs*. 1:2,000 in **Figure 4F**). In contrast, anti-SARS-CoV-2 IgY tested at 400 µg/mL (**Figure 4G**, right panels), a dose below that required for 50% neutralization (~650 µg/mL; **Figure 4E**), was equally effective against both variants. Limited information can be drawn based on a single Moderna vaccinated individual, but other data about the reduced titer of humans immunized with this vaccine towards various variants confirm our findings (37). Together these data indicate that the spectrum of the polyclonal anti-SARS-CoV-2 RBD IgY displays sufficient diversity so that none of the common point mutations in the RBD associated with a greater transmission rate of the virus affected the neutralization efficacy of these most common SARS-CoV-2 variants of concern.

### 3.4 Efficacy Study in a Hamster Model of COVID-19

We did not find *in vivo* efficacy of anti-SARS-CoV-2 RBD IgY in the Syrian golden hamster COVID-19 model against a challenge with a titer of 0.8 or 4 x 10^6^ of SARS-CoV-2 virus, likely because the amount of virus we employed was too high. Lung viral load after 3 days was comparable between the control treatment group and the anti-SARS-CoV-2 RBD IgY treatment group. (**Supplementary Figure S2**). By Day 3, all hamsters lost between 5 and 10% of their weight, without a significant difference between the groups

### 3.5 Rat Toxicity and Safety Study

**Figure 5:**
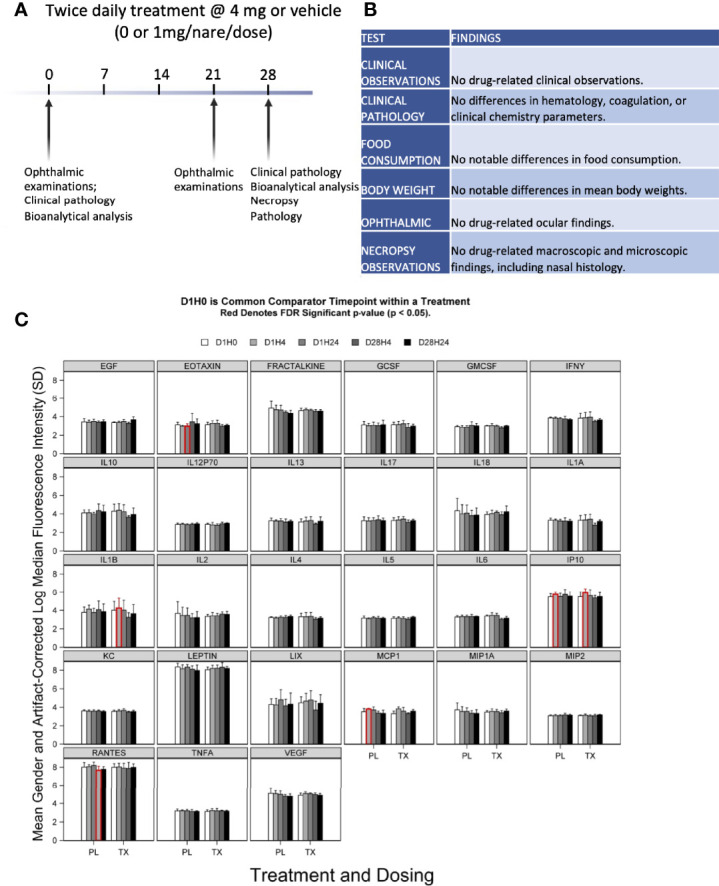
Preclinical toxicity of 28-day treatment with anti-SARS-CoV-2 RBD IgY in rats. **(A)** Study design. **(B)** Summary of findings. **(C)** Serum levels of 27 cytokines over time in rats treated with IgY (Tx) or vehicle/placebo (PL). Data are provided for Day 1 before treatment (D1H0); Day 1, 4 hours after the treatment (D1H4); 24 hours after the two treatments, 6 hours apart, at 24 hours after the first treatment (D1H24); 28 days of twice-daily treatments and 4 hours of the treatment of that day (D28H4); and 28 days of twice-daily treatments and 24 hours of the treatment of that day (D28H24). Red indicates a statistical difference with a false discovery rate (FDR) significant p-value (p < 0.05).

All 8-week-old Sprague Dawley rats (10 males and 10 females) in the 28-day GLP safety study survived to scheduled euthanasia with no mortality, test article-related organ weight changes, or gross or microscopic findings (see **Figures 5A, B**). There were also no differences between female and male groups in each treatment arm. The GLP-qualified assay detected no anti-SARS-CoV-2 RBD IgY in the sera of animals after 28 days of daily treatment with 4 mg anti-SARS-CoV-2 RBD IgY (lower limit of detection of 30 ng/mL).

There was no evidence of significant systemic immune activation in the rats (20/group) treated as above by measuring changes in levels of 27 proinflammatory serum cytokines after anti-SARS-CoV-2 RBD IgY administration (**Figure 5C**). When comparing posttreatment (Day 1 at 4 hours and Day 28 at 24 hours after twice-daily treatments) to pretreatment serum levels (Day 1, time 0; D1 H0), there were no changes for most of the cytokines (22 of the 27 tested) in both placebo- and IgY-treated groups at any time. Compared with D1 H0, we detected a transient, slight increase in interleukin-1 (IL-1) beta and monocyte chemoattractant protein-1 (MCP1) on Day 1, 4 hours after anti-SARS-CoV-2 RBD IgY administration. A similar trend that did not reach significance was also observed in the placebo group at the same time; increases in IL-1 beta and MCP1 were not seen at any other time. There was a significant increase in interferon-inducible protein 10 (IP10) on Day 1, 4 hours after the first treatment compared to D1 H0, observed in both treatment and placebo group. A significant decrease in D1 H24 in the placebo arm was observed only for RANTES. Nonetheless, none of these changes in cytokines were observed in the rats at other time points. Together, our data show that long-term twice-daily administration of 4 mg/mL IgY (or 16 mg/kg) up to 28 days in rats has excellent safety and tolerability with no evidence of systemic immune activation.

### 3.6 Human Tissue Cross-Reactivity Study

**Figure 6:**
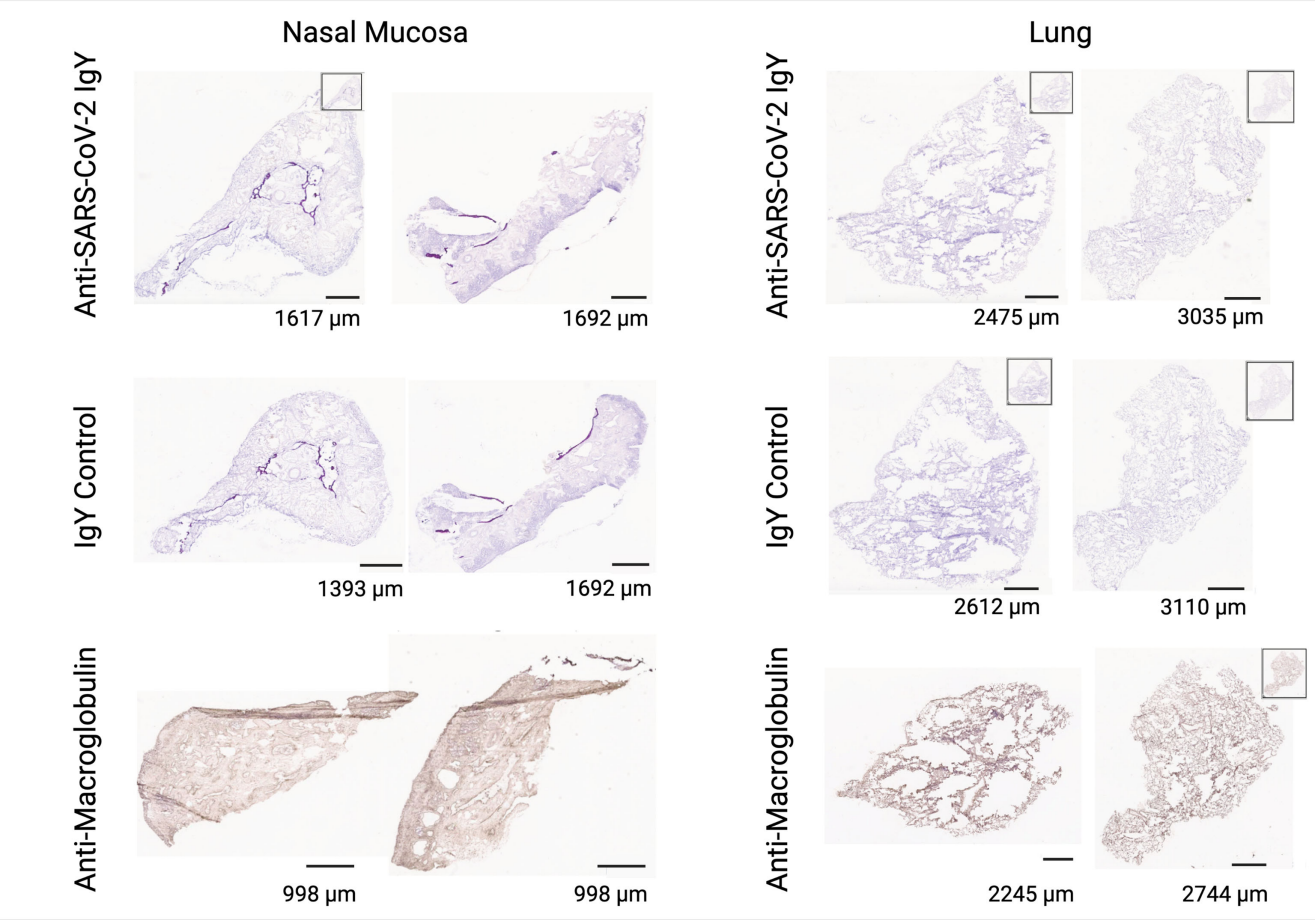
Lack of cross-reactivity of anti-SARS-CoV-2 RBD IgY with human tissues. Immunohistochemical testing of anti-SARS-CoV-2 RBD IgY (top row), control IgY (middle row; negative control), and anti-human macroglobulin antibodies (bottom row; positive control) with human nasal mucosa (left two panels) and human lungs (right two panels). Bars provide a magnification scale.

We determined potential cross-reactivity of the anti-SARS-CoV-2 RBD IgY protein to a full panel of human tissues (at least 3 donors per tissue; see **Supplementary Table S4** for a list of human tissues tested for reactivity). Anti-SARS-CoV-2 RBD IgY reactivity at two concentrations (20 and 10 µg/mL) was compared with control polyclonal chicken IgY antibodies (negative control), and with anti-human macroglobulin (positive control for staining). No specific binding was observed with the anti-SARS-CoV-2 RBD IgY to any of the human tissue panels examined, including human nasal cavity and lung (see **Figure 6**).

### 3.7 Phase 1 Clinical Trial Results

Forty-seven of 48 enrolled participants completed the study drug treatment period and planned study visits (**Supplementary Figure S1**, CONSORT flow diagram). One participant in the multiple-dose part was withdrawn from the study after receiving 3 doses (Day 1) of placebo due to a concurrent upper respiratory tract infection, judged to be unrelated to study drug by the investigator.

#### 3.7.1 Baseline Demographics

Study participants ranged in age from 20 to 43 years (median, 25.5) in the single-dose part of the study and 18 to 40 years (median, 23.0) in the multiple-dose part (**Tables 1**, **2**). Female participants comprised 75% of the population in the single-dose study segment and 46% in the multiple-dose study segment. Demographics are summarized in **Tables 1**, **2**.

**Table 1:** Demographics of participants in single-ascending dose group (Part 1).

Anti-SARS-CoV-2 IgY					
	2 mgN=6	4 mgN=6	8 mgN=6	Placebo N=6	All Participants N=24
**Age, years**					
Mean (SD)	28.8 (7.99)	27.3 (4.76)	27.7 (5.65)	22.7 (2.34)	26.6 (5.72)
Median (range)	26.5 (20-43)	28.0 (21-33)	27.5 (20-37)	22.0 (20-27)	25.5 (20-43)
**Gender, n (%)**					
Female	4 (66.7)	4 (66.7)	4 (66.7)	6 (100)	18 (75.0)
Male	2 (33.3)	2 (33.3)	2 (33.3)	0	6 (25.0)
**Ethnicity, n (%)**					
Hispanic or Latino	1 (16.7)	0	0	0	1 (4.2)
Not Hispanic or Latino	5 (83.3)	6 (100)	6 (100)	6 (100)	23 (95.8)
**Race, n (%)**					
Asian	2 (33.3)	1 (16.7)	0	0	3 (12.5)
Native Hawaiian or OtherPacific Islander	0	0	0	1 (16.7%)	1 (4.2%)
White	4 (66.7)	5 (83.3)	6 (100)	5 (83.3)	20 (83.3)

**Table 2:** Demographics of participants in the multiple-dose group (Part 2).

Anti-SARS-CoV-2 IgY					
	2 mg TID N=6	4 mg TID N=6)	8 mg TID N=6	Placebo TID N=6	All Participants N=24
**Age, years**					
Mean (SD)	26.0 (8.37)	21.8 (2.14)	27.5 (6.92)	25.3 (6.41)	25.2 (6.33)
Median (range)	23.0 (18-40)	22.0 (19-25)	25.0 (21-37)	25.0 (18-36)	23.0 (18-40)
**Gender, n (%)**					
Female	1 (16.7)	4 (66.7)	2 (33.3)	4 (66.7)	11 (45.8)
Male	5 (83.3)	2 (33.3)	4 (66.7)	2 (33.3)	13 (54.2)
**Ethnicity, n (%)**					
Hispanic or Latino	0	0	1 (16.7)	1 (16.7)	2 (8.3)
Not Hispanic or Latino	6 (100)	6 (100)	5 (83.3)	5 (83.3)	22 (91.7)
**Race, n (%)**					
Asian	1 (16.7)	2 (33.3)	2 (33.3)	2 (33.3)	7 (29.2)
Black or African American	0	0	0	1 (16.7)	1 (4.2)
White	5 (83.3)	4 (66.7)	4 (66.7)	3 (50.0)	16 (66.7)

tid, 3-times daily.

#### 3.7.2 Safety and Tolerability

The overall incidence of treatment-emergent adverse events was 29% (7 of 24 participants; **Table 3**) in the single-dose part of the study and 58% (14 of 24 participants; **Table 4**) in the multiple-dose part of the study, with similar incidence rates between anti-SARS-CoV-2 RBD IgY (42%) and placebo (50%) groups. The most frequent treatment-emergent adverse event was headache, with similar rates between placebo (17%) and anti-SARS-CoV-2 RBD IgY (14%) (**Tables 3**, **4**).

**Table 3:** Adverse events by preferred term- single ascending-dose study (Part 1).

	Anti-SARS-CoV-2 IgY				
	2 mg	4 mg	8 mg	Placebo	All Participants
	(N=6)	(N=6)	(N=6)	(N=6)	(N=24)
	n (%) E	n (%) E	n (%) E	n (%) E	n (%) E
Participants with ≥1 TEAE	1 (16.7%) 1	2 (33.3%) 2	2 (33.3%) 2	2 (33.3%) 2	7 (29.2%) 7
**MedDRA Preferred Term**					
Fatigue	0	0	1 (16.7%) 1	1 (16.7%) 1	2 (8.3%) 2
Erythema	0	0	1 (16.7%) 1	0	1 (4.2%) 1
Headache	0	1 (16.7%) 1	0	0	1 (4.2%) 1
Sneezing	0	1 (16.7%) 1	0	0	1 (4.2%) 1
Tension headache	1 (16.7%) 1	0	0	0	1 (4.2%) 1
Thermal burn	0	0	0	1 (16.7%) 1	1 (4.2%) 1

E, number of events; n, number of participants; TEAE, treatment-emergent adverse event.

**Table 4:** Adverse events by preferred term- multiple-dose study (Part 2).

	Anti-SARS-CoV-2 IgY				
	2 mg TID	4 mg TID	8 mg TID	Placebo TID	All Participants
	(N=6)	(N=6)	(N=6)	(N=6)	(N=24)
	n (%) E	n (%) E	n (%) E	n (%) E	n (%) E
Participants with ≥1 TEAE	4 (66.7%) 7	2 (33.3%) 3	4 (66.7%) 5	4 (66.7%) 5	14 (58.3%) 20
**MedDRA Preferred Term**					
Headache	0	0	3 (50.0%) 3	2 (33.3%) 2	5 (20.8%) 5
Upper respiratory tract infection	0	0	1 (16.7%) 1	1 (16.7%) 1	2 (8.3%) 2
Contusion	0	0	0	1 (16.7%) 1	1 (4.2%) 1
Dental discomfort	1 (16.7%) 1	0	0	0	1 (4.2%) 1
Dizziness	1 (16.7%) 1	0	0	0	1 (4.2%) 1
Ear pain	1 (16.7%) 1	0	0	0	1 (4.2%) 1
Epistaxis	1 (16.7%) 1	0	0	0	1 (4.2%) 1
Eyelid irritation	0	0	1 (16.7%) 1	0	1 (4.2%) 1
Injection site hematoma	1 (16.7%) 1	0	0	0	1 (4.2%) 1
Nasal congestion	0	0	0	1 (16.7%) 1	1 (4.2%) 1
Parosmia	0	1 (16.7%) 1	0	0	1 (4.2%) 1
Presyncope	1 (16.7%) 1	0	0	0	1 (4.2%) 1
Rhinorrhea	0	1 (16.7%) 1	0	0	1 (4.2%) 1
Skin abrasion	0	1 (16.7%) 1	0	0	1 (4.2%) 1
Tenderness	1 (16.7%) 1	0	0	0	1 (4.2%) 1

E, number of events; n, number of participants; TEAE, treatment-emergent adverse event.

All adverse events were mild (grade 1) in severity. No serious adverse event or lab-related adverse event was reported, and there was no dose dependency of adverse events observed. Furthermore, no participant receiving anti-SARS-CoV-2 RBD IgY had an adverse event of nasal irritation or nasal congestion. There were no clinically significant observations or trends noted in laboratory assessments, vital signs, physical exam findings, or electrocardiograms during the study.

#### 3.7.3 Pharmacokinetics

PK analyses indicated no evidence of serum anti-SARS-CoV-2 RBD IgY above the lowest detection levels of 30 ng/mL (using a GLP study at Charles River Laboratories) before and after 14 days of treatment in the 18 participants who received anti-SARS-CoV-2 RBD IgY in the multiple-dose part of the study.

#### 3.7.4 Serum Cytokines

Levels of 80 different cytokines in the sera of 19 participants of the multiple-dose part were tested before treatment (D1 pre-dose), 2 hours after dosing on Day 1 (D1 H2); Day 2, before treatment (D2 pre-dose), and 2 hours after the first dosing on day 2 (D2 H2; **Supplementary Figure S3**). There were slight but statistically significant decreases (red histograms) in 10 of the 80 cytokines (**Supplementary Figure S3**). These slight declines in CCL27, CXCL9, IL23, IL27, LIF, MIP5, RESISTIN, TNFα, TNFα, and TNFRSF6 were noted only in the 12 mg/day group compared to the pretreatment levels. These declines also occurred only 2 hours after the first dose (D1 H2) and were not sustained, except for MIP5. There was also a small decrease in IL3, 2 hours after the first dose of 6 mg/day group anti-SARS-CoV-2 RBD IgY, but there were no changes in this cytokine at any other times or doses. These slight changes, which were also not sustained or dose dependent, were judged to be artifactual. Overall, there were no clinically relevant increases in serum cytokines at any time for any of the treatment groups (6, 12, or 24 mg total daily dose of anti-SARS CoV-2 RBD IgY for 14 days).

#### 3.7.5 IgE and Anti-IgE

The anti-SARS-CoV-2 IgY preparation contains ovalbumin. Although participants with egg allergies were excluded from the trial, an exploratory analysis was conducted of the pretreatment serum total IgE and anti-IgE antibody (mainly anti-ovalbumin) levels in the 24 participants in the multiple-dose part of the trial. No participant had detectable serum egg-white specific IgE antibodies (all <0.35 kU/L).

## 4 Discussion

Despite recent successes in generating highly effective COVID-19 vaccines, there is an ongoing need for widely available and safe antiviral strategies that reduce infection and transmission worldwide. Limitations to current vaccines include global vaccine availability and affordability, vaccine hesitancy, and rapidly emerging highly infective viral strains that escape vaccine-induced immunity. This has been particularly apparent following the emergence of Delta and Omicron. The latter variant was first detected in specimens collected on November 8, 2021 (38). Within a few weeks, the variant became the dominant SARS-CoV-2 variant in the United States (39) and is now the most common strain globally (14). Both convalescent sera from early strain-infected patients and fully vaccinated individuals exhibited a low neutralization capacity against Omicron (11–13); a reduction of 30 to 40-fold in neutralization titers was reported. Furthermore, of the eight currently authorized or approved monoclonal antibodies, seven did not neutralize the Omicron variant and one had a 3-fold reduction in neutralization titer (12). These data highlight the need for alternative and complementary approaches to curb COVID-19.

Here, we describe the production of the first chicken egg yolk-derived anti-index SARS-CoV-2 RBD IgY polyclonal antibodies as an intranasal drop product for humans with equal *in vitro* activity against all variants of concern. These IgY were raised in SPF hens and showed an excellent safety profile when given intranasally by drops to rats for 28 days (4 mg/day). No toxicity, innate inflammatory response, or systemic exposure to IgY were noted in this GLP study. In 48 healthy adult participants, anti-SARS-CoV-2 RBD IgY given intranasally at single-ascending doses of 2, 4, and 8 mg and as total daily doses of 6, 12, and 24 mg for 14 days also had a highly favorable safety and tolerability profile. Importantly, no participant receiving intranasal anti-SARS-CoV-2 IgY in the multiple-dose phase had measurable levels of anti-SARS-CoV-2 RBD IgY in their sera, reflecting the absence of systemic absorption of topically administered IgY following intranasal application. We also found no evidence of a systemic inflammatory immune response triggered by the topical treatment with anti-SARS-CoV-2 RBD IgY in humans, and no detectable increase in 80 sera cytokines.

Hen-derived IgY antibodies have several advantages for topical use in humans; these antibodies do not bind the Fc receptor or rheumatoid factor or activate the human complement cascade (40), thus greatly reducing the risk of severe immune responses. These features support the clinical applications of IgY for nasal treatment in a wide range of persons, including the elderly, immunocompromised, and children. IgY antibodies have been beneficial with favorable safety and tolerability when given prophylactically in both animal models and clinical settings of viral diseases, including respiratory infections [reviewed in (41)]. Overall, available data to date suggest that IgY preparations given by nonparenteral administration do not have unwanted off-target proinflammatory effects and are nontoxic to humans, thus permitting potential clinical applications in diverse populations and diseases.

The potential use of IgY from hens immunized with inactivated virus (42), recombinant S protein (43–46), or N protein (47) has been explored with studies evaluating neutralization of the virus in cells, with effective inhibition values, 1 mg/mL (43), 16.8 mg/mL (46), and 0.27 mg/mL (44). However, the ability of these egg-derived antibodies to neutralize other common SARS-CoV-2 variants has not been evaluated and their safety profile in animals or humans has not been assessed.

Intranasally administered proteins are removed from the mucosal surface through ciliary movement (42, 48), which was the basis for using a 3-times daily (every 4 hours) regimen in our phase 1 study. The anti-SARS-CoV-2 RBD IgY was designed to capture and immobilize SARS-CoV-2 on the nasal mucosa, preventing the virus from binding to and spreading across the nasal mucosa, and also preventing the transmission of the virus to other individuals. Intranasal delivery of mammalian immunoglobulins as antiviral agents has been extensively evaluated in humans (49–54). Human immunoglobulins G (IgG) and A given intranasally are well tolerated (49–54), including in pediatric populations. Our decision to protect from viral entry at the nasal mucosa stems from the observation that levels of lung hACE2 are much lower than in the nose; infection of lung tissue is >5 orders of magnitude lower compared with nasal mucosa (55, 56). Therefore, inhibition of viral entry at the nose is likely the correct target site for optimal efficacy.

Our product is egg-derived immunoglobulins, which could contain potentially antigenic residual chicken proteins. However, it is not indicated for those who are allergic to egg yolks. Note also that most humans are exposed to egg-derived antigens through their diet and are not allergic. Furthermore, anaphylaxis for those who consume eggs regularly is rare. However, the safety and tolerability of hen-derived IgY as an intranasal treatment in humans have not been described despite their extensive use in a variety of routes in animals and aquaculture (41, 57).

Several other studies have examined anti-COVID-19 intranasal prophylaxis, mostly in animal models (58–65). These prophylaxes include polymer barriers, active vaccines, existing antiviral drugs, inhibitors of protease-induced activation of the virus, antiseptics, antimicrobial agents, and antibodies. Most relevant for comparison with our study is the use of neutralizing antibodies. In one study, intranasal treatment with a monoclonal human antibody (500 μg in 100 μL/nare) 12 hours after infection in hamsters inoculated with 5 x 10^4^ median tissue culture infective dose (TCID_50_) of SARS-CoV-2 decreased clinical disease signs and improved recovery during the 9 days of infection compared with control antibody-treated hamsters (65). However, in contrast to our study, there was a substantial systemic exposure to the human antibody 24 hours after a single intranasal treatment with 2.5 mg; serum levels of the treated antibodies in that study were 210 ng/mL *vs*. below detection levels (30 ng/mL) in our study following administration of 4 mg IgY antibodies intranasally daily for up to 28 days. Therefore, the benefit of the treatment in their study (65) could have been due to neutralization of the virus that had entered the body rather than blocking entry of the virus at the nasal mucosa. Similarly, a single intranasal monoclonal immunoglobin M (IgM) antibody administration in a mouse model of COVID-19 was highly efficacious when mice were infected with 10^4^ PFU (62). Human IgM systemic exposure was also noted in mice treated with human IgM monoclonal antibody anti-SARS-CoV-2, although the study attributed the protection to the persistent presence of the antibody at the nasal cavity for over 48 hours based on fluorescent tag measurement (60). Such long persistence of levels of IgM in the nasal cavity is at odds with other studies, including when using 99mTc-labeled albumin particles or fluorescently labeled IgY antibodies that showed a residence time of 2-4 hours (42). If the long persistence is not an artifact of the method, it may suggest a unique benefit of IgM treatment as COVID-19 prophylaxis. Note, however, that with one exception (62), none of these studies assessed the cross-reactivity of antibodies against the variants.

Our work shows that, although the IgY was raised against the ancestral (index) strain RBD, the repertoire of the antibodies raised in hens was diverse and polyclonal so that binding affinities measured by ELISA for the single (Alpha), double (Delta), and triple amino acid (Beta) substitutions, or the Omicron variant with 15 amino acid substitutions in the RBD were similar to the affinity for the index RBD or full-length S protein (**Figures 4C, H** **)**. We then confirmed that there was no difference between Alpha, Beta, and Delta variants, and the index strain in a neutralization assay in culture using a VSV-S pseudovirus or live virus (**Figures 4D, E, I** **)**, whereas a reduced neutralization activity of human serum was observed in side-by-side experiments (**Figures 4F, G** **)**. The viral neutralization studies reported here were conducted by a commercial provider (RetroVirox) and by an established laboratory at the USAMRIID, both comparing the results with either convalescent sera or an immunized human, for relative titer evaluation.

The culture neutralization titer of anti-SARS-CoV-2 RBD IgY is lower than the human anti-SARS-CoV-2 sera (e.g., **Figuress 4F vs. 4D**). However, this may reflect the need for protease-induced RBD exposure in the full-length S protein for binding by IgY, which might not occur effectively in the culture model. A comparison of titer values between our product and sera from an immunized person can also be calculated based on values of IgG levels in human sera (~15 mg/mL); a titer of 1:2,000 (**Figure 4F**; dashed line) is equivalent to ~7 ug/mL or 100-fold higher IgG titer than our IgY (**Figure 4D**; ~600 ug/mL). However, as the dose of the IgY anti-SARS-CoV-2 preparation in humans is planned to be 4 mg/dose, more important is the equal potency of the IgY towards the various variants when used even at ~1/10 of the intended IgY dose (**Figures 4E, I** **)**.

Another potentially important difference between our findings and the previously published study is the antigen used to raise the antibodies. When expressed in mammalian cells, the full-length S1 protein has at least 22 glycosylation sites per S monomer (66). As glycosylated amino acids are more immunogenic, the affinity of the human antisera may reflect binding to the glycosylated determinants of the protein. However, as glycosylation sites in the S1 protein are heavily mutated and new sites may be formed in many of the variants (34), immune reactivity that is biased towards glycosylated sites may lead to loss of activity as the virus mutates. This will not occur when the non-glycosylated RBD is used as the immunogen, as we have done in our study using cell-free expressed RBD (22, 23). Supporting the negative impact of glycosylated antigen, increased immunogenicity of protein antigens after removal of glycosylation sites has been previously shown for hepatitis C virus envelope antigen-based vaccines (67). Furthermore, the apparent higher titer in the neutralization assay may be biased if the tested virus has the same glycosylation sites as that used as an immunogen in vaccinated individuals; many studies use the original viral isolates rather than the common current variants.

An important feature of our product is the ease of developing prophylaxis that can be quickly and inexpensively produced. We found that 24 mg total daily dose (divided into three equal doses) of intranasal anti-SARS-CoV-2 IgY for 14 days had an excellent safety profile in humans; this daily dose represents ~1/20 of one egg of immunized SPF hen and ~1/5 of an egg of commercial hen, underscoring that such an IgY dose is feasible for both production cost and effort.

There are some limitations to our studies. Our phase 1 clinical trial in healthy volunteers was to assess initial safety, tolerability, and PK of anti-SARS-CoV-2 IgY and was not designed to evaluate efficacy. In addition, we were unable to obtain *in vivo* data showing viral neutralization (**Supplementary Figure S2**). This may reflect using too much virus in this animal model of COVID-19; our study used 8 x 10^5^ or 4 x 10^6^, *vs*. 1 or 5 x 10^4^ TCID_50_ (63, 65). In addition, our intranasal formulated IgY preparation was viscous to obtain better delivery in humans. As hamsters are obligatory nose-breathers, they may have blown out the formulated IgY. Finally, the virus that was delivered in 50 μL of liquid directly into each nare may have washed out some antibodies. However, a recent study demonstrated a protective effect of intranasal administration of anti-RBD IgY in hamsters challenged with SARS-CoV-2 (64), which suggests that our prophylactic will also be protective. Another limitation in our study is that the neutralization studies comparing the hen IgY *vs*. human sera were not comprehensive and included only 1 or 2 human samples. Nevertheless, our study is the first to demonstrate that anti-SARS-CoV-2 IgY against all the current variants of concern had a favorable safety profile when used chronically as intranasal drops in rats and humans.

There are also several advantages for the use of IgY as prophylaxis against other pathogens besides SARS-CoV-2 that cause disease in humans. As we noted above, IgY generation is inexpensive and fast; one egg of an SPF hen produces 20-80 daily doses (at 6 mg/dose) within 3 weeks from the first injection (1 week after the first boost). We found a limited variability between individual immunized hens as determined by ELISA and Western blot analyses and a batch-to-batch consistency (**Figures 3A-F**). IgY is also easy to distribute; besides the known long-term stability of purified IgY (68), we also found excellent stability of the formulated material at 2-8°C for at least 6 months (maximum time point measured so far) and greater than 2 weeks when stored at room temperature. This is in contrast to vaccines, some of which require cold-chain storage at -80°C that complicates the coordination of global distribution and to resource-poor regions, in particular.

Another important advantage of IgY-based prophylaxis is the ease of local production, including in low- and middle-income countries. As hen immunization is a standard procedure around the world, IgY purification and formulation do not require expensive equipment and are simple to conduct. In a separate study, we developed a step-by-step protocol for IgY purification in low- and middle-income countries using inexpensive, readily available materials in place of costly specialized laboratory equipment and chemicals (69). Therefore, anti-SARS-CoV-2 IgY can be readily made available worldwide as an additional means of reducing SARS-CoV-2 infection. Each day, one immunized hen can produce the daily dose required for prophylaxis of a family. In addition, by reducing viral mobility and anchoring the virus to the nasal mucus, transmission of the virus from infected to healthy individuals may be reduced.

## 5 Conclusion

The current COVID-19 pandemic illustrates the need for prophylactics that can be produced rapidly at low cost, are technically accessible anywhere in the world, and complement traditional vaccine development. The safety and benefit of IgY and the ease to produce it at low cost are well described for animal farms. In contrast, the clinical adaptation of IgY for human use has been slow, likely hampered by a lack of economic benefit that has hindered commercial development by industry. For that reason, we undertook the effort of establishing the ability of anti-SARS-CoV-2 IgY to neutralize variants of concern and the initial safety of the IgY preparation using industry GLP and GMP standards. We suggest that until vaccination that is highly effective against prevalent variants becomes available worldwide or community immunity is achieved, intranasal delivery of anti-SARS-CoV-2 IgY may provide passive immunization, including for use as an add-on to personal protective equipment and other preventive measures for the general population. This IgY may also provide short-term protection in addition to vaccines in less well-ventilated environments, such as trains, airplanes, and lecture halls. We also suggest that this approach has the potential to provide a means to curb new threats of epidemics by airborne infectious agents; by providing the relevant immunogen for hen immunization at the geographical site where the threat was detected, an effective passive immunity can be initiated locally to stop the spread of the airborne infectious agent before it becomes an epidemic. We hope that this study will trigger further work to evaluate the safety and efficacy of anti-SARS-CoV-2 IgY in those at risk of SARS-CoV-2 infection.

## Data Availability Statement

The original contributions presented in the study are included in the article/**Supplementary Material**. Further inquiries can be directed to the corresponding author.

## Ethics Statement

The studies involving human participants were reviewed and approved by Bellberry Human Research Ethics Committee (Australian equivalent to U.S. Institutional Review Board) and the clinical trial was conducted solely at a single investigative site, Linear Clinical Research-Harry Perkins Research Institute (Nedlands, Australia). Stanford Institutional Review Board did not review the research. The study was conducted following the protocol and ethical principles stated in the 2013 version of the Declaration of Helsinki and the applicable guidelines on Good Clinical Practice, and all applicable federal, state, and local laws, rules, and regulations. The patients/participants provided their written informed consent to participate in this study. The animal study was reviewed and approved by Charles River Laboratories Institutional Animal Care and Use Committee before conduct. The hamster efficacy study was conducted following the recommendations in the Guide for the Care and Use of Laboratory Animals of the National Institutes of Health and the protocol was approved by the Institutional Animal Care and Use Committee at the Washington University School of Medicine (assurance number A3381–01). Stanford Institutional Animal Care and Use Committee did not initially review the animal studies because they were not conducted at Stanford but were retroactively approved.

## Author Contributions

Contributions were made by MW, DM-R, and LF to project conceptualization; LF, ML, CS, NO-H, AY, TH, KB, CC, JD, BM, AB, and TJ to methodology; ML, NO-H, GY, CC, CB, SA, TB, AJ, SP, BK, and LL to the investigations; LL and DM-R to visualization; DM-R and SK to funding acquisition; MA, RB, LL, and JR to project administration; JR, JD, BM, TH, AB, SK, and DM-R to supervision; DM-R and LF to writing the original draft; and DM, LF, BK, and TJ to editing. All authors contributed to the article and approved the submitted version.

## Funding

This project was supported by funds from SPARK at Stanford, SPARK GLOBAL, and grants from the Booz-Allen Foundation and ChEM-H (Stanford University). We are also grateful for the financial support from the Moonchu Foundation, the Human Immune Monitoring Center (HIMC) at Stanford University, and the generous monetary donations of many others. The funders had no role in study design, data collection and analysis, decision to publish, or preparation of the manuscript.

## Author Disclaimer

Opinions, conclusions, interpretations, and recommendations are those of the authors and are not necessarily endorsed by the U.S. Army. The mention of trade names or commercial products does not constitute endorsement or recommendation for use by the Department of the Army or the Department of Defense.

## Conflict of Interest

GY, TH, AY, and KB are affiliated with Sutro Biopharma, Inc; NO-H with Charles River Laboratories, Inc; JR, with Linear Clinical Research. Ltd; and BM with Bravado Pharmaceuticals, Inc. DM-R is named on a patent filed for composition and methods for passive immunization against viral infections such as SARS-CoV-2.

The remaining authors declare that the research was conducted in the absence of any commercial or financial relationships that could be construed as a potential conflict of interest.

## Publisher’s Note

All claims expressed in this article are solely those of the authors and do not necessarily represent those of their affiliated organizations, or those of the publisher, the editors and the reviewers. Any product that may be evaluated in this article, or claim that may be made by its manufacturer, is not guaranteed or endorsed by the publisher.

## Abbreviations

COVID-19, coronavirus disease 2019; ELISA, enzyme-linked immunosorbent assay; GLP, Good Laboratory Practice; GMP, Good Manufacturing Practice; hACE2, human angiotensin-converting enzyme 2; HPLC, high-performance liquid chromatography; HRP, horseradish peroxidase; IC_50_, half-maximal inhibitory concentration; IgE, immunoglobulin E; IgG, immunoglobulin G; IgM, immunoglobulin M; IgY, immunoglobulin Y; NT_50_, 50% neutralization titer; PFU, plaque-forming units; PK, pharmacokinetic; RBD, receptor-binding domain; S, spike; SARS-CoV-2, severe acute respiratory syndrome coronavirus 2; SDS-PAGE, sodium dodecyl sulfate-polyacrylamide gel electrophoresis; SPF, specific pathogen-free; SMC, Safety Monitoring Committee; TCID_50,_ tissue culture infective dose; USAMARIID, United States Army Medical Research Institute of Infectious Diseases; VSV, vesicular stomatitis virus; VSV-S, VSV expressing spike protein on its surface.

## References

[1] World Health Organization. WHO Coronavirus (COVID-19) Dashboard (2022). Available at: https://covid19.who.int (Accessed May 1, 2022).

[2] University of Oxford. Vaccination by Location (2022). Available at: https://ourworldindata.org/covid-vaccinations?country=OWID_WRL (Accessed May 1, 2022).

[3] Irwin A. What it Will Take to Vaccinate the World Against COVID-19. Nature (2021) 592:176–8. doi: 10.1038/d41586-021-00727-3 33767468

[4] Lopman BA Shioda K Nguyen Q Beckett SJ Siegler AJ Sullivan PS. A Framework for Monitoring Population Immunity to SARS-CoV-2. Ann Epidemiol (2021) 63:75–8. doi: 10.1016/j.annepidem.2021.08.013 PMC8379082 34425208

[5] Goldblatt D. SARS-CoV-2: From Herd Immunity to Hybrid Immunity. Nat Rev Immunol (2022) 579:1–2. doi: 10.1038/s41577-022-00725-0 PMC9016684 35440758

[6] Koff WC Schenkelberg T Williams T Baric RS McDermott A Cameron CM. Development and Deployment of COVID-19 Vaccines for Those Most Vulnerable. Sci Transl Med (2021) 13:eabd1525. doi: 10.1126/scitranslmed.abd1525 33536277

[7] Pardi N Weissman D. Development of Vaccines and Antivirals for Combating Viral Pandemics. Nat BioMed Eng (2020) 4:1128–33. doi: 10.1038/s41551-020-00658-w PMC8336060 33293724

[8] Mlcochova P Kemp S Dhar MS Papa G Meng B Ferreira IATM. SARS-CoV-2 B.1.617.2 Delta Variant Replication and Immune Evasion. Nature (2021) 599:114–9. doi: 10.1038/s41586-021-03944-y PMC8566220 34488225

[9] Liu C Ginn HM Dejnirattisai W Supasa P Wang B Tuekprakhon A. Reduced Neutralization of SARS-CoV-2 B.1.617 by Vaccine and Convalescent Serum. Cell (2021) 184:4220–6. doi: 10.1016/j.cell.2021.06.020 PMC8218332 34242578

[10] National Institutes of Health, National Center for Advancing Translational Sciences, in: OpenData Portal. SARS-CoV-2 Variants & Therapeutics. Available at: https://opendata.ncats.nih.gov/variant/summary (Accessed April 22, 2022).

[11] Zhang Z Wu S Wu B Yang Q Chen A Li Y. SARS-CoV-2 Omicron Strain Exhibits Potent Capabilities for Immune Evasion and Viral Entrance. Sig Transduct Target Ther (2021) 6:430. doi: 10.1038/s41392-021-00852-5 PMC8678971 34921135

[12] Cameroni E Bowen JE Rosen LE Saliba C Zepada SK Culap K. Broadly Neutralizing Antibodies Overcome SARS-CoV-2 Omicron Antigenic Shift. Nature (2022) 602:664–70. doi: 10.1038/s41586-021-04386-2 PMC9531318 35016195

[13] Liu L Iketani S Guo Y Chan JF-W Wang M Liu L. Striking Antibody Evasion Manifested by the Omicron Variant of SARS-CoV-2. Nature (2022) 602:676–81. doi: 10.1038/s41586-021-04388-0 35016198

[14] University of Oxford. SARS-CoV-2 Variants in Analyzed Sequences (by Country). Available at: https://ourworldindata.org/grapher/covid-variants-area?country=~USA (Accessed May 1, 2022).

[15] Hu B Guo H Zhou P Shi Z-L. Characteristics of SARS-CoV-2 and COVID-19. Nat Rev Microbiol (2021) 19:141–54. doi: 10.1038/s41579-020-00459-7 PMC7537588 33024307

[16] Walls AC Park Y-J Tortorici MA Wall A McGuire AT Veesler D. Structure, Function, and Antigenicity of the SARS-CoV-2 Spike Glycoprotein. Cell (2020) 181:281–92.e6. doi: 10.1016/j.cell.2020.02.058 32155444 PMC7102599

[17] Murin CD Wilson IA Ward AB. Antibody Responses to Viral Infections: A Structural Perspective Across Three Different Enveloped Viruses. Nat Microbiol (2019) 4:734–47. doi: 10.1038/s41564-019-0392-y PMC6818971 30886356

[18] Weltzin R Monath TP. Intranasal Antibody Prophylaxis for Protection Against Viral Disease. Clin Microbiol Rev (1999) 12:383–93. doi: 10.1128/CMR.12.3.383 PMC100244 10398671

[19] Xu Y Li X Jin L Zhen Y Lu Y Li S. Application of Chicken Egg Yolk Immunoglobulins in the Control of Terrestrial and Aquatic Animal Diseases: A Review. Biotechnol Adv (2011) 29:860–8. doi: 10.1016/j.biotechadv.2011.07.003 PMC7126572 21787857

[20] Michael A Meenatchisundaram S Parameswari G Subbraj T Selvakumaran R Ramalingam S. Chicken Egg Yolk Antibodies (IgY) as an Alternative to Mammalian Antibodies. Indian J Sci Technol (2010) 3:468–74. doi: 10.17485/ijst/2010/v3i4.24

[21] Pauly D Chacana PA Calzado EG Brembs B Schade R. IgY Technology: Extraction of Chicken Antibodies From Egg Yolk by Polyethylene Glycol (PEG) Precipitation. J Vis Exp (2011) 51:3084. doi: 10.3791/3084 PMC3197133 21559009

[22] Cai Q Hanson JA Steiner AR Tran C Masikat MR Chen R. A Simplified and Robust Protocol for Immunoglobulin Expression in Escherichia Coli Cell-Free Protein Synthesis Systems. Biotechnol Prog (2015) 31:823–31. doi: 10.1002/btpr.2082 PMC5029582 25826247

[23] Zawada JF Yin G Steiner AR Yang J Naresh A Roy SM. Microscale to Manufacturing Scale-Up of Cell-Free Cytokine Production - a New Approach for Shortening Protein Production Development Timelines. Biotechnol Bioeng (2011) 108:1570–8. doi: 10.1002/bit.23103 PMC3128707 21337337

[24] Rehan IF Youssef M Abdel-Rahman MAM Fahmy SG Ahmed E Ahmed AS. The Impact of Probiotics and Egg Yolk IgY on Behavior and Blood Parameters in a Broiler Immune Stress Model. Front Vet Sci (2020) 7:145. doi: 10.3389/fvets.2020.00145 32328501 PMC7160245

[25] Hussein MA Rehan IF Rehan AF Eleiwa NZ Abdel-Rahman MAM Fahmy SG. Egg Yolk IgY: A Novel Trend of Feed Additives to Limit Drugs and to Improve Poultry Meat Quality. Front Vet Sci (2020) 7:350. doi: 10.3389/fvets.2020.00350 32760743 PMC7371932

[26] Rehan IF Rehan AF Abouelnaga AF Hussein MA El-Ghareeb WR Eleiwa NZ. Impact of Dietary Egg Yolk IgY Powder on Behavior, Meat Quality, Physiology, and Intestinal Escherichia Coli Colonization of Broiler Chicks. Front Vet Sci (2022) 9:783094. doi: 10.3389/fvets.2022.783094 35425829 PMC9004463

[27] Rehan I Elnagar A. Chicken Egg Yolk-IgY: Passive Immunization Promising Targeted Therapy of COVID-19 Pandemic. J Appl Vet Sci (2021) 6:67–91. doi: 10.21608/JAVS.2021.164324

[28] Chan JF-W Zhang AJ Yuan S Poon VK-M Chan CC-S Lee AC-Y. Simulation of the Clinical and Pathological Manifestations of Coronavirus Disease 2019 (COVID-19) in a Golden Syrian Hamster Model: Implications for Disease Pathogenesis and Transmissibility. Clin Infect Dis (2020) 71:2428–46. doi: 10.1093/cid/ciaa325 PMC7184405 32215622

[29] Bricker TL Darling TL Hassan AO Harastani HH Soung A Jiang X. A Single Intranasal or Intramuscular Immunization With Chimpanzee Adenovirus-Vectored SARS-CoV-2 Vaccine Protects Against Pneumonia in Hamsters. Cell Rep (2021) 20:36. doi: 10.1016/j.celrep.2021.109400 PMC8238649 34245672

[30] Neerukonda SN Vassell R Herrup R Liu S Wang T Takeda K. Establishment of a Well-Characterized SARS-CoV-2 Lentiviral Pseudovirus Neutralization Assay Using 293T Cells With Stable Expression of ACE2 and TMPRSS2. PloS One (2021) 16:e0248348. doi: 10.1371/journal.pone.0248348 33690649 PMC7946320

[31] Kollberg H Carlander D Olesen H Wejåker PE Johannesson M Larsson A. Oral Administration of Specific Yolk Antibodies (IgY) may Prevent Pseudomonas Aeruginosa Infections in Patients With Cystic Fibrosis: A Phase I Feasibility Study. Pediatr Pulmonol (2003) 35:433–40. doi: 10.1002/ppul.10290 12746939

[32] Pokhrel S Kraemer BR Lee L Samardzic K Mochly-Rosen D. Increased Elastase Sensitivity and Decreased Intramolecular Interactions in the More Transmissible 501Y.V1 and 501Y.V2 SARS-CoV-2 Variants’ Spike Protein-an in Silico Analysis. PloS One (2021) 16:e0251426. doi: 10.1371/journal.pone.0251426 34038453 PMC8153447

[33] Pokhrel S Kraemer BR Burkholz S Mochly-Rosen D. Natural Variants in SARS-CoV-2 Spike Protein Pinpoint Structural and Functional Hotspots With Implications for Prophylaxis and Therapeutic Strategies. Sci Rep (2021) 11:13120. doi: 10.1038/s41598-021-92641-x 34162970 PMC8222349

[34] World Health Organization. Tracking SARS-CoV-2 Variants. Available at: https://www.who.int/en/activities/tracking-SARS-CoV-2-variants (Accessed April 22, 2022). 37184162

[35] Chemical Computing Group. Molecular Operating Environment (MOE). Montreal, Canada (2021).

[36] Dejnirattisai W Shaw RH Supasa P Liu C Stuart AS Pollard AJ. Reduced Neutralisation of SARS-CoV-2 Omicron B.1.1.529 Variant by Post-Immunisation Serum. Lancet (2021) 399:S0140–6736:02844-0. doi: 10.1016/S0140-6736(21)02844-0 PMC8687667 34942101

[37] Choi A Koch M Wu K Dixon G Oestreicher J Legault H. Serum Neutralizing Activity of mRNA-1273 Against SARS-CoV-2 Variants. J Virol (2021) 95:e01313–21. doi: 10.1128/JVI.01313-21 PMC8577347 34549975

[38] Centers for Disease Control and Prevention. Science Brief: Omicron (B.1.1.529) Variant (December 2, 2021). Available at: https://www.cdc.gov/coronavirus/2019-ncov/science/science-briefs/scientific-brief-omicron-variant.html (Accessed April 22, 2022). 34932278

[39] Centers for Disease Control and Prevention. Potential Rapid Increase of Omicron Variant Infections in the United States (December 20, 2021). Available at: https://www.cdc.gov/coronavirus/2019-ncov/science/forecasting/mathematical-modeling-outbreak.html (Accessed April 22, 2022).

[40] Carlander D Stålberg J Larsson A. Chicken Antibodies: A Clinical Chemistry Perspective. Ups J Med Sci (1999) 104:179–89. doi: 10.3109/03009739909178961 10680951

[41] Lee L Samardzic K Wallach M Frumkin LR Mochly-Rosen D. Immunoglobulin Y for Potential Diagnostic and Therapeutic Applications in Infectious Diseases. Front Immunol (2021) 12:696003. doi: 10.3389/fimmu.2021.696003 34177963 PMC8220206

[42] Shen H Cai Y Zhang H Wu J Ye L Yang P. Anti-SARS-CoV-2 IgY Isolated From Egg Yolks of Hens Immunized With Inactivated SARS-CoV-2 for Immunoprophylaxis of COVID-19. Virol Sin (2021) 36:1080–2. doi: 10.1007/s12250-021-00371-1 PMC8137804 34019233

[43] Bao L Zhang C Lyu J Yi P Shen X Tang B. Egg Yolk Immunoglobulin (IgY) Targeting SARS-CoV-2 S1 as Potential Virus Entry Blocker. J Appl Microbiol (2022) 132:2421–30. doi: 10.1111/jam.15340 PMC8657347 34706134

[44] Wei S Duan S Liu X Wang H Ding S Chen Y. Chicken Egg Yolk Antibodies (IgYs) Block the Binding of Multiple SARS-CoV-2 Spike Protein Variants to Human ACE2. Int Immunopharmacol (2021) 90:107172. doi: 10.1016/j.intimp.2020.107172 33191178 PMC7608017

[45] Lu Y Wang Y Zhang Z Huang J Yao M Huang G. Generation of Chicken IgY Against SARS-COV-2 Spike Protein and Epitope Mapping. J Immunol Res (2020) 2020:9465398. doi: 10.1155/2020/9465398 33134398 PMC7568776

[46] Artman C Brumfield KD Khanna S Goepp J. Avian Antibodies (IgY) Targeting Spike Glycoprotein of Severe Acute Respiratory Syndrome Coronavirus 2 (SARS-CoV-2) Inhibit Receptor Binding and Viral Replication. PloS One (2021) 16:e0252399. doi: 10.1371/journal.pone.0252399 34048457 PMC8162713

[47] Lyu J Bao L Shen X Yan C Zhang C Wei W. The Preparation of N-IgY Targeting SARS-CoV-2 and its Immunomodulation to IFN-γ Production In Vitro. Int Immunopharmacol (2021) 96:107797. doi: 10.1016/j.intimp.2021.107797 34162159 PMC8133490

[48] Marttin E Schipper GM Verhoef JC Merkus WHM. Nasal Mucociliary Clearance as a Factor in Nasal Drug Delivery. Adv Drug Delivery Rev (1998) 29:13–38. doi: 10.1016/s0169-409x(97)00059-8 10837578

[49] Keller MA Stiehm ER. Passive Immunity in Prevention and Treatment of Infectious Diseases. Clin Microbiol Rev (2000) 13:602–14. doi: 10.1128/CMR.13.4.602 PMC88952 11023960

[50] Hemmingsson P Hammarström L. Nasal Administration of Immunoglobulin as Effective Prophylaxis Against Infections in Elite Cross-Country Skiers. Scand J Infect Dis (1993) 25:783–5. doi: 10.3109/00365549309008580 8052822

[51] Lindberg K Berglund B. Effect of Treatment With Nasal IgA on the Incidence of Infectious Disease in World-Class Canoeists. Int J Sports Med (1996) 17:235–8. doi: 10.1055/s-2007-972838 8739580

[52] Giraudi V Riganti C Torales MR Sédola H Gaddi E. Upper Respiratory Infections in Children: Response to Endonasal Administration of IGA. Int J Pediatr Otorhinolaryngol (1997) 39:103–10. doi: 10.1016/s0165-5876(96)01472-3 9104618

[53] Heikkinen TA Ruohola A Ruuskanen O Waris M Uhari M Hammarström L. (1998) 17:367–72. doi: 10.1097/00006454-199805000-00004 9613647

[54] Gleich GJ Yunginger JW. Ragweed Hay Fever: Treatment by Local Passive Administration of IgG Antibody. Clin Allergy (1975) 5:79–87. doi: 10.1111/j.1365-2222.1975.tb01838.x 1053432

[55] Hou YJ Okuda K Edwards CE Martinez DR Asakura T Dinnon KH 3rd. SARS-CoV-2 Reverse genetics reveals a variable infection gradient in the respiratory tract. Cell (2020) 182:429–446.e14. doi: 10.1016/j.cell.2020.05.042 32526206 PMC7250779

[56] Mason RJ. Pathogenesis of COVID-19 From a Cell Biology Perspective. Eur Resp J (2020) 55:2000607. doi: 10.1183/13993003.00607-2020 PMC7144260 32269085

[57] Kovacs-Nolan J Mine Y. Egg Yolk Antibodies for Passive Immunity. Ann Rev Food Sci Technol (2012) 3:163–82. doi: 10.1146/annurev-food-022811-101137 22136128

[58] Kim Y-II Kim D Yu K-M Seo HD Lee S-A Casel MAB. Development of Spike Receptor-Binding Domain Nanoparticles as a Vaccine Candidate Against SARS-CoV-2 Infection in Ferrets. mBio (2021) 12:e00230–21. doi: 10.1128/mBio.00230-21 PMC8092224 33653891

[59] Palit P Chattopadhyay D Thomas SS Kundu A Kim HS Rezaeif N. Phytopharmaceuticals Mediated Furin and TMPRSS2 Receptor Blocking: Can it be a Potential Therapeutic Option for Covid-19? Phytomedicine (2021) 85:153396. doi: 10.1016/j.phymed.2020.153396 33380375 PMC7591300

[60] Burton MJ Clarkson JE Goulao B Glenny A-M McBain AJ Schilder AG. Use of Antimicrobial Mouthwashes (Gargling) and Nasal Sprays by Healthcare Workers to Protect Them When Treating Patients With Suspected or Confirmed COVID-19 Infection. Cochrane Database Syst Rev (2021) 9:CD013626. doi: 10.1002/14651858.CD013626.pub2 PMC8202127 32936949

[61] Higgins TS Wu AW Illing EA Sokoloski KJ Weaver BA Anthony BP. Intranasal Antiviral Drug Delivery and Coronavirus Disease 2019 (COVID-19): A State of the Art Review. Otolaryngol Head Neck Surg (2021) 163:682–94. doi: 10.1177/0194599820933170 32660339

[62] Ku Z Xie X Hinton PR Liu X Ye X Muruato AE. Nasal Delivery of an IgM Offers Broad Protection From SARS-CoV-2 Variants. Nature (2021) 595:718–23. doi: 10.1038/s41586-021-03673-2 PMC8742224 34082438

[63] Nambulli S Xiang Y Tilston-Lunel NL Rennick LJ Sang Z Klimstra WB. Inhalable Nanobody (PiN-21) Prevents and Treats SARS-CoV-2 Infections in Syrian Hamsters at Ultra-Low Doses. Sci Adv (2021) 7:eabh0319. doi: 10.1126/sciadv.abh0319 34039613 PMC8153718

[64] Agurto-Arteaga A Rios-Matos D Choque-Guevara R Montesinos-Millán R Montalván Á Isasi-Rivas G. Preclinical Assessment of IgY Antibodies Against Recombinant SARS-CoV-2 RBD Protein for Prophylaxis and Post-Infection Treatment of COVID-19. Front Immunol (2022) 13:881604. doi: 10.3389/fimmu.2022.881604 35664008 PMC9157249

[65] Fu Y Maruyama J Singh A Lim R Ledesma A Lee D. Protective Effects of Sti-2020 Antibody Delivered Post-Infection by the Intranasal or Intravenous Route in a Syrian Golden Hamster COVID-19 Model. bioRxiv (2020). doi: 10.1101/2020.10.28.359836v1

[66] Watanabe Y Berndsen ZT Raghwani J Seabright GE Allen JD Pybus OG. Vulnerabilities in Coronavirus Glycan Shields Despite Extensive Glycosylation. Nat Commun (2020) 11:2688. doi: 10.1038/s41467-020-16567-0 32461612 PMC7253482

[67] Fournillier A Wychowski C Boucreux D Baumert TF Meunier JC Jacobs D. Induction of Hepatitis C Virus E1 Envelope Protein-Specific Immune Response can be Enhanced by Mutation of N-Glycosylation Sites. J Virol (2001) 75:12088–97. doi: 10.1128/JVI.75.24.12088-12097.2001 PMC116104 11711599

[68] Schade R Gutierrez Calzado E Sarmiento R Chacana PA Porankiewicz-Asplund J Terzolo HR. Chicken Egg Yolk Antibodies (IgY-Technology): A Review of Progress in Production and Use in Research and Human and Veterinary Medicine. Altern Lab Anim (2005) 33:129–54. doi: 10.1177/026119290503300208 16180988

[69] Chen CJ Hudson AF Jia AS Kunchur CR Song AJ Tran E. Affordable IgY-Based Antiviral Prophylaxis for Resource-Limited Settings to Address Epidemic and Pandemic Risks. J Glob Health (2022) 12:5009. doi: 10.7189/jogh.12.05009 PMC8877785 35265332

[70] Frumkin LR Lucas L Scribner CL Ortega-Heinly N Rogers J Yin G. Egg-Derived Anti-SARS-CoV-2 Immunoglobulin Y (IgY) With Broad Variant Activity as Intranasal Prophylaxis Against COVID-19. medRxiv (2022). doi: 10.1101/2022.01.07.22268914 PMC9199392 35720389

