# Supplementary material for: Egg-Derived Anti-SARS-CoV-2 Immunoglobulin Y (IgY) With Broad Variant Activity as Intranasal Prophylaxis Against COVID-19
Source: Front Immunol. 2022 Jun 1;13:899617. doi: 10.3389/fimmu.2022.899617 (PMC9199392; doi:10.3389/fimmu.2022.899617)
Supplement: Supplementary file 1 [file DataSheet_1.pdf]

Supplementary Material

Table of Contents

Supplementary Table S1 Experimental protocol of the rat toxicity study.

Supplementary Table S2 Bioanalytical sample collection from the rat toxicity study.

Supplementary Table S3 Cytokine sample collection for the rat toxicity study.

Supplementary Table S4 Human tissue cross-reactivity; list of tissues examined.

Supplementary Figure S1 CONSORT flow diagram of phase 1 single-ascending and multiple-dose study.

Supplementary Figure S2 Hamster efficacy study.

Supplementary Figure S3 Serum cytokines for human participants in the phase 1 study

Supplementary Methods
1.1 Study Design
1.2 Recombinant SARS-CoV-2 RBD
  1.2.1. Characterization of the cell-free expressed recombinant RBD using surface plasmon resonance
1.3 Hen immunization and IgY purification and characterization
1.4 Evaluation of IgY
  1.4.1. ELISA evaluation of IgY titer against SARS-CoV-2 variants of concern
  1.4.2 In culture viral neutralization studies using pseudovirus
  1.4.3 In culture neutralization studies using live virus
  1.4.4 CPE-based neutralization assay
1.5 GMP IgY formulation, analytical studies, and stability
1.6 GLP rat toxicity and safety study
  1.6.1 Cytokine level measurement and analysis
  1.6.2 IgY in sera of treated rats
1.7 Human tissue cross-reactivity study
1.8 Efficacy study in a hamster model of COVID-19
  1.8.1 Study protocol: hamster infection with SARS-CoV-2
  1.8.2 Virus titration assays from hamster samples
1.9 Statistical Analysis

Supplementary Methods, Protocol Protocol for "Phase 1 Study in Healthy Participants to Evaluate the Safety, Tolerability, and Pharmacokinetics of Single-Ascending and Multiple Doses of an Anti-Severe Acute Respiratory Syndrome Coronavirus 2 (SARS-CoV-2) Chicken Egg Antibody (IgY)"

Supplementary Table S1. Experimental protocol of the rat toxicity study

Group No.	Test Material	Dose Volume (µL dose)	Dose Concentration (mg/mL)	No. of Animals			
				Main Study		Toxicokinetic Study	
				Males	Females	Males	Females
1	Control Article	100	0	10	10	3	3
2	IgY SARS-CoV-2 RBD	100	20	10	10	6	6
3	Control Article	100	0	3[a]	3[a]	-	-
4	IgY SARS-CoV-2 RBD	100	20	3[a]	3[a]	-	-

[a] = These animals were for cytokine analysis only.

Supplementary Table S2. Bioanalytical sample collection from the rat toxicity study

Group No.	Subgroup	No. of Animals (M/F)	(Time Post the first daily dose) on Day 1		
			**15 min**	**6[a] hr**	**24 hr**
1	A	3/3	X	-	X
2	A	3/3	X	-	X
	B	3/3	-	X	-

**Method/Comments:**	Venipuncture of the jugular vein (under isoflurane anesthesia). The tail vein may be used if blood cannot be obtained via the jugular vein.
**Target Volume (mL):**	0.3
**Anticoagulant:**	K_2EDTA
**Special requirements:**	Tubes will be chilled after blood collection.
**Processing:**	Plasma

X = Sample to be collected; M = Male; F = Female; - = Not applicable
[a] Sample to be collected before the second daily dose.

S3 Table. Cytokine sample collection for the rat toxicity study

Group No.	No. of Animals (M/F)	(Time Post the first daily dose) on Day 28		
		0[a] hr	4 hr	24 hr
3	3/3	X	X	X
4	3/3	X	X	X

**Method/Comments:**	Venipuncture of the jugular vein (under isoflurane anesthesia). The tail vein may be used if blood cannot be obtained via the jugular vein.
**Target Volume (mL):**	0.3
**Anticoagulant:**	K_2EDTA
**Special requirements:**	Tubes will be chilled on wet ice after blood collection.
**Processing:**	Plasma

X = Sample to be collected; M = Male; F = Female; - = Not applicable

[a] Sample to be collected before dosing.

Supplementary Table S4. Human tissue cross-reactivity; list of tissues examined.

Binding of anti-SARS-CoV-2 RBD IgY was determined using the following normal human tissue from 3 separate donors.

Adrenal	Liver	Spinal Cord
Bladder (urinary)	Lung	Spleen
Blood Cells [a]	Lymph Node	Striated Muscle (skeletal)
Blood Vessels (endothelium) [b]	Ovary	Testis
Bone Marrow	Nasal Mucosa	Thymus
Brain - cerebellum	Pancreas	Thyroid
Brain - cerebral cortex	Parathyroid	Tongue
Breast (mammary gland)	Peripheral Nerve	Tonsil
Eye	Pituitary	Trachea
Fallopian Tube (oviduct)	Placenta	Ureter
Gastrointestinal (GI) Tract [c]	Prostate	Uterus - cervix
Heart	Salivary Gland	Uterus- endometrium
Kidney (glomerulus, tubule)	Skin	

[a] Evaluated from peripheral blood smears.

[b] Evaluated from all tissues where present.

[c] Included esophagus, large intestine/colon, small intestine, and stomach (including underlying smooth muscle).

Supplementary Figure S1. CONSORT flow diagram of phase 1 single-ascending and multiple-dose study.

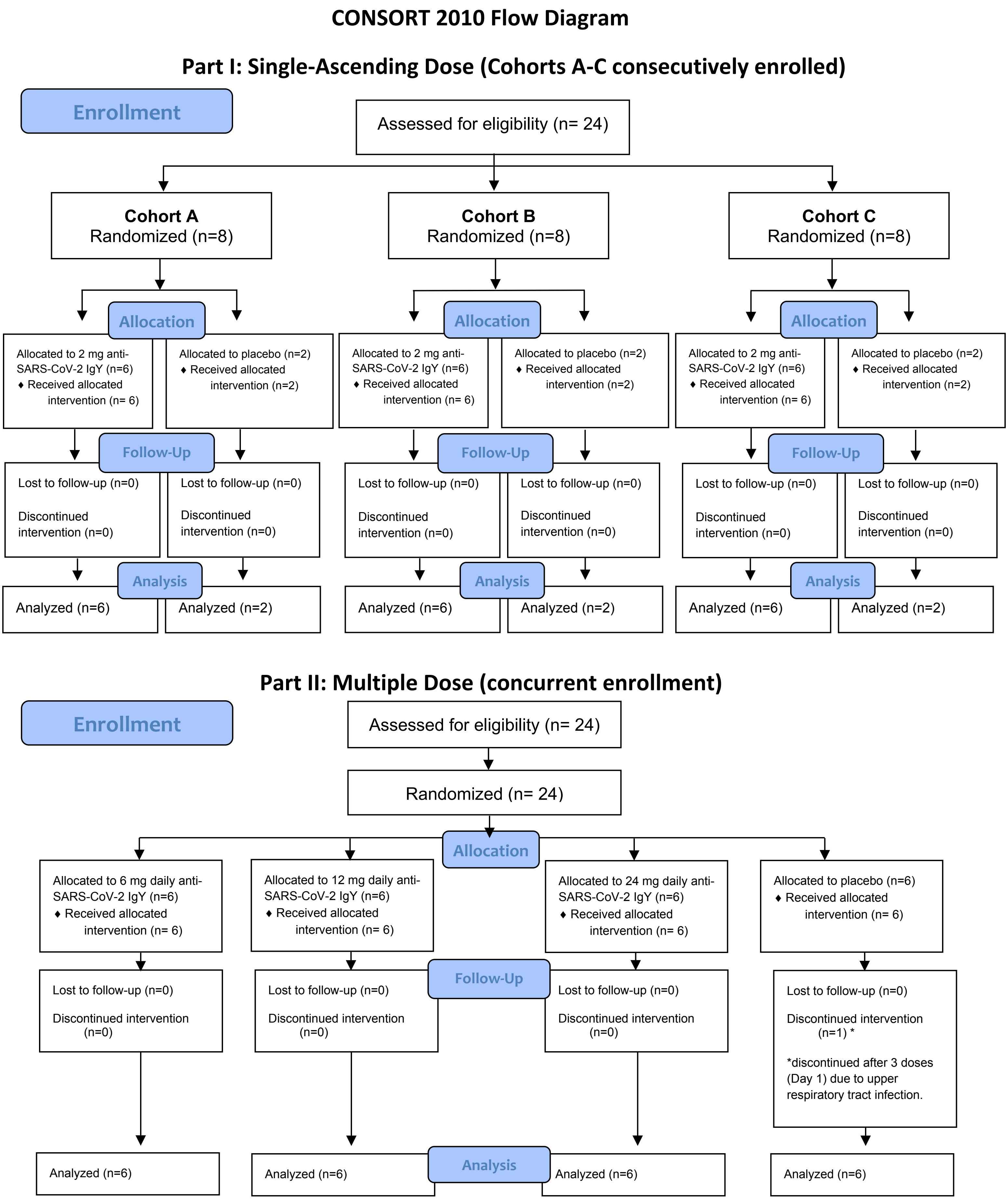

Supplementary Figure 2. Hamster efficacy study

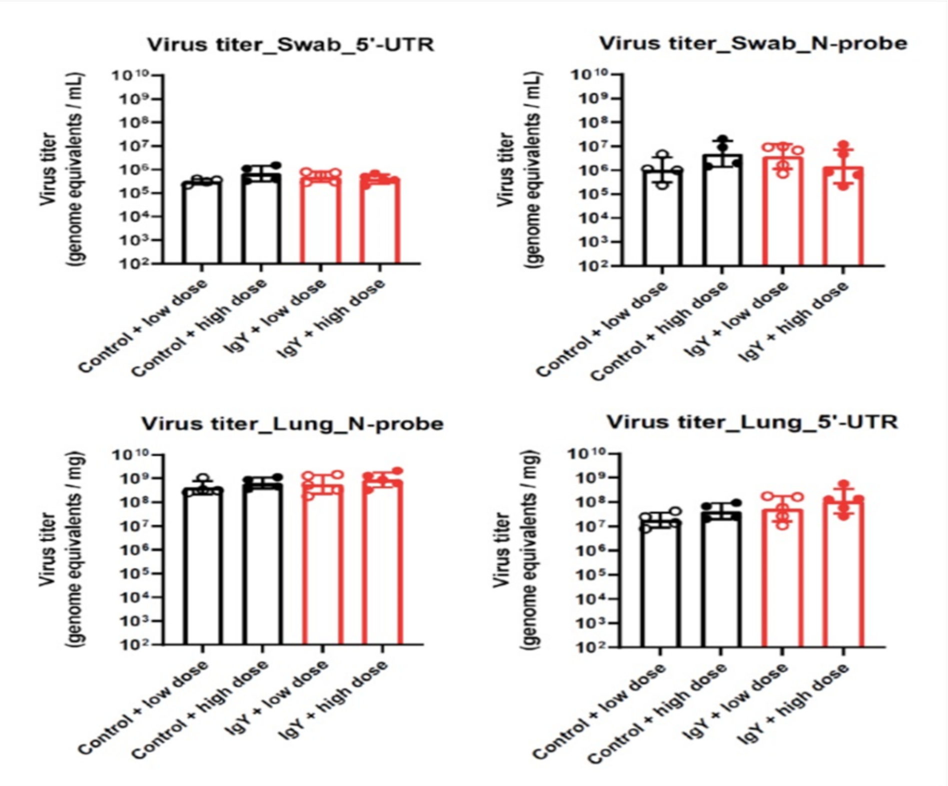

Placebo control and IgY-treated Syrian hamsters were challenged with a high (4 x 10^6) or 'low' (8 x 10^5) dose of SARS-CoV-2. Infectious virus titers were measured in the lungs 3 days post-infection by plaque assay. Each symbol is a single animal. One animal in the placebo group challenged with a low dose of the virus did not have any detectable virus in the lung homogenate. This was ruled an outlier based on the viral RNA levels in other assays. Viral RNA levels were quantified RT-PCR in lung homogenates of nasal swabs using 2 different primer-probe sets. Each symbol is a single animal and the bars represent the geometric mean with geometric standard deviation.

Supplementary Figure S3 (next page). Serum cytokines for human participants in the phase 1 study.

Blood cytokine levels before treatment (D1 pre-dose), 2 hours after the first treatment (D1H2), 24 hours after 3-times daily intranasal (D2 pre-dose), and 2 hours after the first dose on the second day (D2H2) of anti-SARS-CoV-2 RBD IgY (0, 2, 4 and 8 mg/dose or 0, 6, 12, and 24 mg/day). D1 pre-dose is common comparator within a treatment. Red denotes false discovery rate (FDR) significant p-value ($p<0.05$).

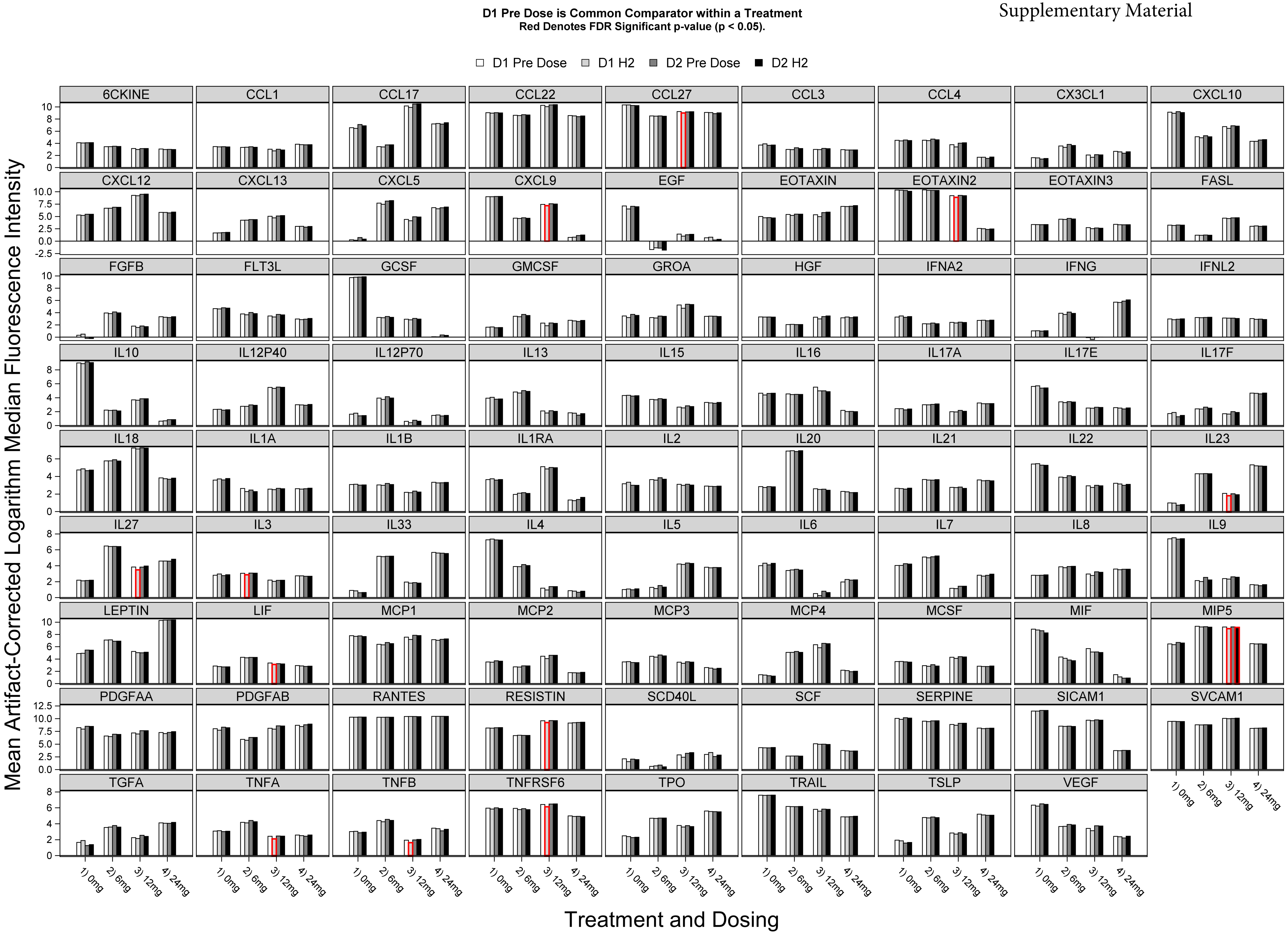
D1 Pre Dose is Common Comparator within a Treatment
Red Denotes FDR Significant p-value (p < 0.05).
D1 Pre Dose
D1 H2
D2 Pre Dose
D2 H2
6CKINE
CCL1
CCL17
CCL22
CCL27
CCL3
CCL4
CX3CL1
CXCL10
CXCL12
CXCL13
CXCL5
CXCL9
EGF
EOTAXIN
EOTAXIN2
EOTAXIN3
FASL
FGFB
FLT3L
GCSF
GMCSF
GROA
HGF
IFNA2
IFNG
IFNL2
IL10
IL12P40
IL12P70
IL13
IL15
IL16
IL17A
IL17E
IL17F
IL18
IL1A
IL1B
IL1RA
IL2
IL20
IL21
IL22
IL23
IL27
IL3
IL33
IL4
IL5
IL6
IL7
IL8
IL9
LEPTIN
LIF
MCP1
MCP2
MCP3
MCP4
MCSF
MIF
MIP5
PDGFAA
PDGFAB
RANTES
RESISTIN
SCD40L
SCF
SERPINE
SICAM1
SVCAM1
TGFA
TNFA
TNFB
TNFRSF6
TPO
TRAIL
TSLP
VEGF
1) 0mg
2) 6mg
3) 12mg
4) 24mg
Mean Artifact-Corrected Logarithm Median Fluorescence Intensity
Treatment and Dosing

1. MATERIALS AND METHODS

1.1 Study Design

The experiments were conducted in four parts: 1) production of immunogen (recombinant RBD), immunization of 12 SPF hens, IgY collection from egg yolks, and in vitro characterization of the IgY anti-SARS CoV-2 RBD, 2) Good Laboratory Practice (GLP) blinded safety studies in rat, treated intranasally twice daily for 28 days with a total of 16 mg/kg IgY or vehicle, 3) a preliminary efficacy study of hamsters treated with IgY or phosphate-buffered saline (PBS) for 4 hours before viral challenge, and 4) a placebo-controlled, double-blind phase 1 safety, tolerability, and pharmacokinetic (PK) study conducted in healthy humans using intranasal IgY or vehicle in single-ascending doses followed by multiple doses (3-times daily every 4 hours) for 14 days. Both the single-ascending and multiple-dose parts were followed by a 7-day nontreatment period to further evaluate safety.

1.2 Recombinant SARS-CoV-2 RBD

SARS-CoV-2 RBD (residues 328-533) of the 2019 novel coronavirus index virus (2019-nCoV) was expressed in cell-free protein synthesis reactions at Sutro Biopharma, Inc. (South San Francisco, CA) using the XpressCFTM platform and was constructed as a carboxy-terminal fusion to a small ubiquitin-related modifier (SUMO) sequence to enable the production of a tagless RBD protein post enzymatic cleavage. The his-SUMO tag was constructed as previously described with an N-terminal his6-tag followed by a GGS-linker and the yeast SUMO protein Smt3 for Ulp1-enabled cleavage.

Briefly, cell-free reactions were prepared by the addition of 37.5% v/v iodoacetamide-treated S30 extract, 5 µg/mL plasmid, and a supermix containing amino acids, nano-microspheres, and small molecules for energy generation. T7 RNA polymerase was over-expressed in Escherichia coli and added to the cell-free reaction as a reagent lysate at <1% v/v. Reactions were carried out in a DASbox stirred tank (Eppendorf) at 250 mL volume with pH, dissolved oxygen, and temperature control. Reactions were run for 16 hours at a temperature of 25°C, pH was controlled at 7.0 using 1 M citrate and 1 M potassium hydroxide, and dissolved oxygen was maintained at 20%.

The XpressCF reaction of SARS-CoV-2-RBD 328-533 was clarified by centrifugation at 10,000 rpm for 20 minutes (Beckman, JLA-10.500 rotor) and filtered through a 0.22-µm membrane filter. The clarified material was loaded onto a 5-mL his-Trap Excel affinity column equilibrated with binding buffer (15 mM Tris-HCl, 500 mM NaCl, pH 8.0). After 20 column volumes were applied to wash unbound impurities, the bound proteins were eluted with 20 mM Tris-HCl, 300 mM imidazole, pH 8.0. The eluted fractions were then analyzed by 4-12% sodium dodecyl sulfate-polyacrylamide gel electrophoresis (SDS-PAGE) and protein concentrations were determined by measuring the absorbance at 280 nm by NanoDrop (Thermo Fisher Scientific). The his-SUMO tag was removed by Ulp1 protease digestion for 1 hour at room temperature. The digested reaction was analyzed by 4-12% SDS-PAGE to verify full cleavage of the his-SUMO tag before flow-through mode purification by Capto Q (Cytiva). Twenty mM Tris-HCl, pH 8.0, was used to equilibrate the column, and the flow-through containing the cleaved protein was collected. To further purify the CoV-2-RBD, the Capto Q flow-through fraction was bound to a Capto SP ImpRes cation column equilibrated with 20 mM Tris-HCl, pH 8.0. The bound protein was eluted with a 30-column volume linear gradient using elution buffer (20 mM Tris-HCl, 300 mM NaCl, pH 8.0). An Amicon Ultra-15, 3kD centrifugal filter was used to concentrate and buffer exchange the target peak fraction into 6% sucrose in PBS at pH 7.2. The final purity of the product was demonstrated by high-performance liquid chromatography (HPLC) analysis.

1.2.1. Characterization of the cell-free expressed recombinant RBD using surface plasmon resonance

Binding kinetic of cell-free expressed SARS-CoV-2 RBD construct or mammalian expressed his-tagged RBD control (ACROBiosystems SPD-C52H1) were then measured on a

Biacore T200 instrument, using Fc-tagged hACE2 receptor protein (ACROBiosystems AC2-H5257). To this end, an anti-human Fc antibody (Jackson ImmunoResearch Labs) was immobilized on all flow cells of a CM5 chip (GE Healthcare). Fc-tagged human ACE2 receptor protein (ACROBiosystems AC2-H5257) was captured at ~100 replication units (RU). Binding of cell-free expressed SARS-CoV-2 S protein RBD construct or mammalian expressed RBD control (ACROBiosystems SPD-C52H1) was measured at concentrations up to 100 nM. Kinetic experiments were performed at 25°C at a flow rate of 50 µL/min. RBD samples were diluted in HBS-EP buffer (Teknova) and injected over the chip for 180 seconds followed by a 420-second dissociation. The chip was regenerated with 10 mM glycine pH 1.5 after each injection. Affinities were calculated using Biacore T200 Evaluation Software.

1.3 Hen immunization and IgY purification and characterization

Nine SPF hens were obtained from Charles River Laboratories and housed in a filtered air, positive pressure barrier room (3 more hens from the same lot were added to the study after 5 months) at Avian Vaccine Services, Charles River Laboratories (Storrs, CT). Each hen was caged individually with access to feed and water. Upon receipt, hens were immunized with an inoculum containing 50 µg of recombinant cell-free expressed RBD fragment derived from S1 spike protein and a water-in-oil adjuvant. Test bleeds were taken before the first immunization and every 2 weeks after the first immunization throughout the project. Serum samples from each hen were tested using an indirect enzyme-linked immunoassay (ELISA) and Western blot. Hens received a boost 14 days after immunization and every 4 weeks after the previous immunization, unless otherwise indicated.

Eggs were collected weekly. Yolks in batches up to 100 eggs/batch were separated and IgY was purified. ELISA titration of IgY binding to full-length S1 (ACROBiosystems, S1N-C5255) expressed in human 293 cells and the cell-free expressed RBD of the SARS-CoV-2 spike protein was performed in 96-well plates. IgY was then purified from the yolks. Eggs were collected weekly and egg white was separated from the egg yolk and discarded. Yolks were stored frozen at -20˚C. For purification of IgY, yolks were thawed and diluted 1:10 in sterile PBS. The pH was reduced to 5.0 and the yolk solution was incubated at 4˚C overnight. The yolk solution was then centrifuged for 10 minutes at 9000 rpm. The supernatant was removed, pooled, the pH was increased to 7.0, and final filtration (0.45 µm) was performed.

Hen sera and purified IgY were tested by ELISA and Western blot analysis using both the immunogen (RBD) and full length glycosylated S1 protein (ACROBiosystems, cat# S1N-C5255). Lot Y0120 of purified IgY was derived from 50 yolks, starting 2 weeks after the primary immunization. Lot Y0130 was derived from the next 50 yolks, starting 3 weeks after the primary immunization. Lot Y0140 and above were derived from the next 100 yolks collected over the preceding 2 weeks. Lots of purified IgY were tested by indirect ELISA and Western blot to determine antibody response levels within the yolk after purification as well as affinity to the cell-free expressed RBD fragment of S1 spike protein and the entire glycosylated S1 protein (ACROBiosystems, cat# S1N-C5255). Protein concentration was also measured using the Bradford assay. IgY purity was determined using SDS-PAGE under reducing conditions and stained with Coomassie Blue.

ELISA titration of IgY binding to full length and the RBD of the SARS-CoV-2 spike protein was performed as follows: 96-well clear flat bottom polystyrene high-binding ELISA plates (Corning, Cat# 9018) were coated with 100 µl/well of 5 µg/mL recombinant his-tagged (Sino Biological) RBD protein overnight at 4°C. Plates were then washed twice with PBS and blocked with 1% bovine serum albumin (BSA)-PBS. After blocking, 100 µL of serial dilutions of IgY was added to the wells and incubated at room temperature for 2 hours. The plates were then washed twice and incubated with horseradish peroxidase (HRP)-conjugated mouse rat anti-chicken IgY (Sapphire, Cat# LO-IgY-16-P-0.5) 1:2000 diluted in 0.5% BSA and 0.05% Tween-20 in PBS) for 60 minutes. After

3 final washes using 0.05% PBS-Tween, the plates were read using a chemiluminescent substrate (Pierce TMB Substrate Kit, Thermo Fisher, Cat# 34021). Luminescence was read on a plate reader and analyzed with SoftMaxPro 6.0 (Molecular Devices).

Hen sera were diluted with 1% BSA at various dilutions and tested in ELISA. One hundred microliters were added to wells and the plate was incubated at room temperature for 1 hour. Plate wells were then rinsed with 1% Tween 20 wash solution, as indicated above. Anti-chicken HRP conjugate (Invitrogen, cat# SA-19509) diluted 1:5000 was added to each well. The plate was incubated at room temperature for 1 hour, followed by another 1% Tween 20 wash. 3,3′,5,5′-tetramethylbenzidine substrate (KPL, cat# 5120-0050) was added to each well and the plate was incubated at room temperature for 15 minutes. Optical density was measured using an ELISA reader (Molecular Devices) at 650 nm.

Western blot analysis was performed with the cell-free expressed RBD fragment of the index S1 protein or the entire mammalian-expressed S1 protein (ACROBiosystems). One microgram of denatured protein was added to Mini-PROTEAN TGX 4-20% SDS-PAGE. Gel was run at 200V for 45 minutes in Tris-glycine buffer and stained using Simply Blue Safestain (Invitrogen) at room temperature for 1 hour. Gels were destained twice using deionized water.

An unstained SDS-PAGE gel was transferred to the polyvinylidene difluoride membrane using Trans-Blot Turbo Transfer System (Bio-Rad Laboratories) and cut into strips. Strips were then stored at -20˚C until ready to use. Five percent nonfat dry milk was added to strips and incubated at room temperature for 30-60 minutes. Test bleed sera and IgY were diluted 1:2000 and purified IgY was diluted 1:500 in 0.2 µm-filtered PBS and added to strips. Strips were incubated at room temperature for 2 hours followed by 3 washes in filtered PBS. Rabbit anti-chicken IgY (H+L) secondary antibody, HRP (Invitrogen) diluted 1:3000 was added to each strip and incubated at room temperature for 1-2 hours. The strips were washed once again in filtered PBS. Opti-4CN substrate (Bio-Rad Laboratories) was added to each strip and incubated at room temperature for up to 30 minutes in a rocking incubator. Strips were washed twice in deionized water and images were taken using Bio-Rad EZ Gel Imager.

1.4 Evaluation of IgY

1.4.1. ELISA evaluation of IgY titer against SARS-CoV-2 variants of concern

ELISA titer of the final IgY preparation used in the clinical studies against the Alpha, Beta, Delta, and Omicron-derived RBD [amino acids 319-537; ACROBiosystems SPD-C52H1(index), SPD-C52Hn (Alpha), SPD-C52Hp (Beta), SPD-C525e (Delta), and SPD-C522e (Omicron)]) was carried out as described above.

1.4.2 In culture viral neutralization studies using pseudovirus

The neutralization assays using pseudovirus were performed at RetroVirox (San Diego, CA). The assay used 3 non-replicative vesicular stomatitis virus (VSV) pseudoviruses carrying a firefly luciferase reporter gene and expressing the following S protein of SARS-CoV-2 on the surface: the index SARS-CoV-2 Hu-1 spike, a truncated spike with a C-terminal 19 amino acid deletion; the Beta variant (K417N/ E484K / N501Y / D614G full-length spike); and a D614G spike variant with a full-length sequence of the index spike protein. The IgY preparation or plasma was diluted in Dulbecco's Modified Eagle Medium (DMEM) with 5% fetal bovine serum (FBS) to perform pre-incubation with pseudovirus before addition to target cells. The neutralization assay was performed with HEK 293T-hACE2, a human embryonic kidney cell line overexpressing hACE2. Test item was preincubated for 60 minutes at 37°C with a previously titrated inoculum of pseudovirus. The mixture was then added to 293T-hACE2 cells and infection was allowed for 24 hours. Pseudovirus infection was determined by measuring firefly luciferase activity (RLU, relative light unit ratio) 24 hours after infection. Putative neutralizing antibodies (human sera, purified IgY, or vehicle) were present in the cell culture for the entire duration of the experiment. The ability of the test item to neutralize pseudovirus

carrying SARS-CoV-2 spike was compared with samples treated with vehicle alone. Nine concentrations of the IgY sample were tested in duplicates with each variant in 5-fold serial dilutions starting at 10,000 μg/mL. The half-maximal inhibitory concentration (IC_{50}) and 50% neutralization titer (NT_{50}) values for the IgY and positive control human serum were determined using GraphPad Prism software (GraphPad Software).

Quality controls for the pseudovirus neutralization assays were performed to determine: 1) signal-to-background values; 2) variation of the assay, estimated as the average of the coefficient of variation for data points for which 50% or greater infection (RLUs) was observed compared to cells with pseudovirus in the presence of vehicle alone, and 3) neutralization with plasma from an individual who received 2 doses of the Moderna (mRNA-1273) COVID-19 vaccine. All controls worked as anticipated for each assay.

1.4.3 In culture neutralization studies using live virus

At the United States Army Medical Research Institute of Infectious Diseases (USAMARIID; Frederick, MD), all work with authentic (live) SARS-CoV-2 was completed in Biosafety Level 3 laboratories following federal and institutional biosafety standards and regulations. Vero-76 cells were inoculated with SARS-CoV-2/Was1 (MT020880.1) at a multiplicity of infection of 0.01 and incubated at 37°C with 5% CO_2 and 80% humidity. At 50 hours post-infection, cells were frozen at -80°C for 1 hour, allowed to thaw at room temperature, and supernatants were collected and clarified by centrifugation at ~2,500 ×g for 10 minutes. The clarified supernatant was aliquoted and stored at -80°C.

Authentic (live) SARS-CoV-2/B.1.617.2 at a multiplicity of infection of 1 was incubated for 1 hour at 37°C with serially diluted antibodies. Vero-E6 monolayers were exposed to the antibody-virus mixture at 37°C for 1 hour. Following incubation, the viral inoculum was removed and fresh cell culture media was added for an additional 23 hours at 37°C. Cells were washed with PBS, fixed in 10% formalin, and permeabilized with 0.2% Triton-X for 10 minutes. Detection of infection was accomplished using an anti-SARS-CoV-2 nucleocapsid protein detection antibody (Sino Biological), and a goat α-rabbit secondary antibody conjugated to AlexaFluor488. Infected cells were identified using the PerkinElmer Operetta high-content imaging instrument and data analysis was performed using the Harmony software (PerkinElmer).

At RetroVirox (San Diego, CA), purified IgY (lot Y0180) was tested against three live SARS-CoV-2 clinical isolates: MEX-BC15/2021 (lineage B.1.617.2, Delta), USA/SD-RVX01/2022 (lineage B.1.529, Omicron) and MEX-BC2/2020 (lineage B.1, carrying the D614G mutation). A virus-induced cytopathic effect (CPE)-based neutralization assay was performed by infecting Vero E6 cells in the presence or absence of test items. Vero E6 cells were maintained in DMEM with 10% FBS. Twenty-four hours after cell seeding, test samples were submitted to serial dilutions with DMEM with 2% FBS in a different plate. Then, virus diluted in the same media alone was pre-incubated with test items for 1 hour at 37°C in a humidified incubator. Following incubation, the media was removed from the cells. The cells were then challenged with the SARS-CoV-2/antibody pre-incubated mix. The amount of viral inoculum was previously titrated to result in a linear response inhibited by antivirals with known activity against SARS-CoV-2. Cell culture media with the virus inoculum was not removed after virus adsorption, and antibodies and virus were maintained in the media for the duration of the assay (96 hours). After this period, the extent of cell viability was monitored with the neutral red uptake assay.

The virus-induced CPE was monitored under the microscope after 3 days of infection, and cells were stained with neutral red to monitor cell viability the following day. Viable cells incorporate neutral red in their lysosomes. The uptake of neutral red relies on the ability of live cells to maintain the pH inside the lysosomes lower than in the cytoplasm, a process that requires ATP. Inside the lysosome, the dye becomes charged and is retained. After a 3-hour incubation with neutral red (0.017%), the extra dye is washed away, and the neutral red is extracted from lysosomes by

incubating cells for 15 minutes with a solution containing 50% ethanol and 1% acetic acid. The amount of neutral red is estimated by measuring absorbance at 540 nm in a plate reader.

Antibodies were evaluated in triplicates using five-fold serial dilutions starting at 10.0 mg/mL. Controls included uninfected cells and infected cells treated with vehicle alone. Some cells were treated with plasma from an uninfected individual who received two doses of Moderna's mRNA COVID-19 vaccine as a positive control.

1.4.4 CPE-based neutralization assay

The average absorbance at 540 nm (A540) observed in infected cells in the presence of vehicle alone was calculated, and then subtracted from all samples to determine the inhibition of the virus-induced CPE. Data points were then normalized to the average A540 signal observed in uninfected cells after subtraction of the absorbance signal observed in infected cells. In the neutral red CPE-based neutralization assay, uninfected cells remained viable and uptake the dye at higher levels than non-viable cells. In the absence of test items, the virus-induced CPE kills infected cells and leads to lower A540 (this value equals 0% inhibition). In contrast, incubation with neutralizing agents prevents the virus-induced CPE and leads to absorbance levels similar to those observed in uninfected cells. Full recovery of cell viability in infected cells represents 100% neutralization of the virus.

1.5 GMP IgY formulation, analytical studies, and stability

Using Good Manufacturing Practice (GMP), anti-S1 RBD IgY was formulated for use as intranasal drops as 0 (placebo control), 5, 10, and 20 mg/mL anti-S1 RBD IgY preparations in sterile 2% microcrystalline cellulose and carboxymethylcellulose sodium at Bravado Pharmaceuticals (Lutz, FL). The suspension was packed in a 1.5-mL dropper bottle and tests for GMP drug product release complied with the relevant standards and methods, including microbiological examination of nonsterile products (USP <1111>, <61> and <62>). All formulated products were 100% stable as measured by physical and analytical properties, including HPLC, when stored for at least 6 months at 2-8°C and about 1 month when stored at room temperature. Ten and 20 mg/mL solutions stored at room temperature showed 100% analytical stabilities for 3 months. However, several samples had some physical visual abnormalities (slight discoloration and opacity) that occurred beginning at 1 month when stored at room temperature. Therefore, all formulated samples used in the subsequent studies were stored at 2-8°C. For stability determination, formulated IgY (20 mL) was chromatographed on a TSK Gel G3000swxl sizing column (with guard column) on HPLC at 0.4 mL/min at room temperature, and the eluate was monitored at 214 nm. For the rat toxicity and safety study, and for the human study, we used only 20 mg/mL as the highest dose because it was readily soluble.

1.6 GLP rat toxicity and safety study

Thirty-five female and 35 male >8-week-old Sprague Dawley rats were used in a GLP study conducted at Charles River Laboratories (Spencerville, OH). Rats were identified using a subcutaneously implanted electronic identification chip and were acclimated to their housing for at least 4 days before the first day of dosing. Animals were randomly assigned to groups; males and females were randomized separately and housed in accordance with the United States Department of Agriculture (USDA) Animal Welfare Act (9 CFR, Parts 1, 2, and 3) and as described in the Guide for the Care and Use of Laboratory Animals.

For psychological and environmental enrichment, a hiding device, a chewing object, and edible enrichment treats were offered throughout the study and a cycle of 12 hours light and 12 hours dark was maintained. Water and food were freely available. The experimental protocol is summarized in **Supplementary Tables S1-S3**.

Detailed clinical observation was conducted once a week and included body weight and food consumption. Ophthalmic examinations were conducted before treatment and during the last week of dosing by a veterinary ophthalmologist and included a short-acting mydriatic solution treatment to

each eye to facilitate the ocular examinations. Clinical laboratory assessments included the presence of circulating IgY (in serum) and standard hematology, coagulation, clinical chemistry, and urinalysis parameters tested at pretreatment and Day 28 (last day of treatment) after 4 hours of fasting.

Hematology parameters included red blood cell count, hemoglobin concentration, hematocrit, mean corpuscular volume, red blood cell distribution width, mean corpuscular hemoglobin concentration, mean corpuscular hemoglobin, reticulocyte count (absolute), platelet count, white blood cell count, neutrophil count (absolute), lymphocyte count (absolute), monocyte count (absolute), eosinophil count (absolute), basophil count (absolute), large unstained cells (absolute) and other cells (as appropriate). Clinical chemistry parameters included alanine aminotransferase, aspartate aminotransferase, alkaline phosphatase, gamma-glutamyltransferase, creatine kinase, total bilirubin, urea nitrogen, creatinine, calcium, phosphorus, total protein, albumin, globulin (calculated), albumin/globulin ratio, glucose, cholesterol, triglycerides, sodium, potassium, chloride, and sample quality. Urinalysis included color, appearance/clarity, specific gravity, volume, pH, protein, glucose, bilirubin, ketones, and blood.

1.6.1 Cytokine level measurement and analysis

This non-GLP blinded assay was performed on serum by the Immunoassay Team at the Human Immune Monitoring Center at Stanford University (Stanford, CA). Assay kits (RECYMAG65K27PMX Rat) were purchased from EMD Millipore and used according to the manufacturer's recommendations, with modifications described as follows. Briefly, samples were mixed with antibody-linked magnetic beads on a 96-well plate and incubated overnight at 4°C while shaking. Cold and room temperature incubation steps were performed on an orbital shaker at 500-600 rpm. Plates were washed twice with wash buffer in a Biotek ELx405 washer. Following 1-hour incubation at room temperature with a biotinylated detection antibody, streptavidin-PE was added for 30 minutes while shaking. Plates were washed as described above. PBS was then added to wells for reading in the Luminex FLEXMAP 3D Instrument with a lower bound of 50 beads per sample per cytokine. Each sample was measured in duplicate. Custom Assay Chex control beads were purchased from Radix BioSolutions and added to all wells.

Median fluorescence intensity data were corrected for plate and nonspecific binding artifacts and regressed on the time point. Regression analysis employed linear mixed models in SAS®/STAT (SAS Institute) with a separate model fit for each treatment and cytokine, and with separate sets of models with and without gender as a covariate, for 108 models in total. Of the 108 models, 84% met the assumption of normally distributed residuals and 78% for random effects.

1.6.2 IgY in sera of treated rats

The presence of anti-SARS-CoV-2 IgY in the sera of rats was evaluated using GLP standards in a qualified ELISA assay at Charles River Laboratories (Reno, NV). ELISA 96-well plates were coated with goat anti-chicken IgY (Thermo Fisher; A16056). After blocking, the plates were incubated with the samples containing anti-SARS-CoV-2 IgY at various concentrations for 1 hour at room temperature. After washing the microplate, rabbit anti-chicken IgY (Thermo Fisher; A16130) - HRP conjugate was added and incubated for 1 hour at room temperature. The ELISA plate was washed and substrate (3,3',5,5'-tetramethylbenzidine) added to the wells and incubated for 20 minutes. The color development was stopped by the addition of 2N sulfuric acid and the color intensity measured in a microplate reader at 450 nm. A calibration curve from the absorbance values was obtained from the standards using a 4-parameter curve fit with a weighting equation of 1/y2. The concentrations of anti-SARS-CoV-2 IgY in the samples were determined from the calibration curve. After 4 analytical method validations, lower and upper limit of detection, intra- and inter-assay precision and accuracy, dilution integrity, and short-term stability, IgY levels in the blood samples of rats before and after 28 days of treatment were below the limit of detection.

1.7 Human tissue cross-reactivity study

A GLP study examining human tissue reactivity of the anti-SARS-CoV-2 RBD IgY was conducted at Charles River Laboratories (Frederick, MD) using at least 3 tissues from at least 3 donors (**Supplementary Table S4**). Ten mL of 10 mg/mL or 20 mg/mL IgY control (negative control) and anti-SARS-CoV-2 RBD IgY or an anti-human hypercalcemia of malignancy peptide (amino acid residues 1-34, Sigma-Aldrich; Catalog No. H9148; positive control), each in 1% BSA, were incubated for 1 hour with acetone-fixed human tissues (normal) of at least 3 separate donors after 20 minutes of incubation, with block solution of PBS + 1% BSA, 0.5% casein and 5% normal rabbit serum. After PBS washes, the secondary antibody (peroxidase [HRP]-conjugated rabbit anti-chicken IgY; Testing Facility antibody tracking No. A45764; of 2 µg/mL) was added for 30 minutes. After PBS washes, DAB (3,3′-diaminobenzidine) was applied for 4 minutes as a substrate for the peroxidase reaction. All slides were rinsed with tap water, counterstained, dehydrated, and mounted for visualization. For the β2-microglobulin antibody (positive control staining) 1 µg/mL of antibodies were incubated with the slides for 1 hour and bound antibodies were detected with biotinylated secondary antibody (goat anti-rabbit IgG; 2 µg/mL) for 30 minutes, as above. The slides were then visualized by light microscopy.

1.8 Virus preparation for the efficacy study in a hamster model of COVID-19 and quantitation of viral load in the animals

Syrian hamsters develop mild-to-moderate disease with progressive weight loss that starts several days after SARS-CoV-2 infection by intranasal inoculation. SARS-CoV-2 (strain 2019-nCoV/USA-WA1/2020) was propagated on Vero-TMPRSS2 cells, and the virus titer was determined by plaque assays on Vero-hACE2 and Vero-hACE2-TMPRSS2 cells. Briefly, cells were seeded in 24-well plates, and the next day, virus stocks were serially diluted 10-fold, starting at 1:10, in cell infection medium [Minimum Essential Media (MEM) containing 2% FBS, L-glutamine, penicillin, and streptomycin]. Two hundred and fifty microliters of the diluted virus were added to a single well per dilution per sample. After 1 hour at 37°C, the inoculum was aspirated and a 1% methylcellulose overlay in MEM supplemented with 2% FBS was added. Seventy-two hours after virus inoculation, the cells were fixed with 4% formalin and the monolayer was stained with crystal violet (0.5% w/v in 25% methanol in water) for 1 hour at 20°C. The number of plaques was counted and used to calculate the plaque-forming units (PFU)/mL.

1.8.1 Study protocol: hamster infection with SARS-CoV-2

Five- to 6-week-old Syrian golden hamsters (Charles River Laboratories) infected with SARS-CoV-2 as previously described were housed at the Washington University (St. Louis, MO) Biosafety Level 3 facility in HEPA-filtered rodent cages. Before challenge with SARS-CoV-2, all animals received 100 µL of a placebo control fluid or fluid-formulated anti-SARS-CoV-2 RBD IgY (1 mg/50 mL of the 20 mg/mL solution per nare). The amount of nasal anti-SARS-CoV-2 RBD IgY drops was limited by the volume that could be given per nare. Four hours after the delivery, the animals were challenged with 10^4 or 5 x 10^4 plaque-forming units (PFU) of SARS-CoV-2 (titer determined on Vero-hACE2; see below). The protocol included 18 hamsters in 4 planned groups: Group 1: Control with 5 x 10^4 PFU (4 animals); Group 2: Control with 1 x 10^4 PFU (4 animals); Group 3: Anti-SARS-CoV-2 RBD IgY with 5 x 10^4 PFU (5 animals); and Group 4: Anti-SARS-CoV-2 RBD IgY with 1 x 10^4 PFU (5 animals). Note that the viral titer in Vero-hACE2-hTMPRSS2 cells, which express the essential protease to liberate the RBD from the S protein on the surface of the virus, was subsequently found to be almost 100-fold higher for Groups 1 and 3 (4 x 10^6 PFU) and Groups 2 and 4 (0.8 x 10^6 PFU).

Animal weight was recorded daily. Three days after challenge, the animals were sacrificed and lungs were collected. The left lobe was homogenized in 1.0 mL of DMEM and the clarified

supernatant was used for viral load analysis by plaque assay and quantitative reverse transcription PCR.

1.8.2 Virus titration assays from hamster samples

Plaque assays were performed on Vero-hACE2 and Vero-hACE2-TMPRSS2 cells in 24-well plates. Lung tissue homogenates were serially diluted 10-fold, starting at 1:10, in cell infection medium (DMEM containing 2% FBS, L-glutamine, penicillin, and streptomycin). Two hundred and fifty microliters of the diluted lung homogenates were added to a single well per dilution per sample. After 1 hour at 37°C, the inoculum was aspirated and a 1% methylcellulose overlay in MEM supplemented with 2% FBS was added. Seventy-two hours after virus inoculation, the cells were fixed with 4% formalin, and the monolayer was stained with crystal violet (0.5% w/v in 25% methanol in water) for 1 hour at 20°C. The number of plaques was counted and used to calculate the PFU/mL. To quantify viral load in lung tissue homogenates, RNA was extracted from 100 µL samples using a QIAamp viral RNA mini kit (Qiagen) and eluted with 50 µL of water. RNA (4 µL) was used for real-time quantitative reverse transcription PCR to detect and quantify N gene levels of SARS-CoV-2 using TaqMan™ Fast Virus 1-Step Master Mix (Applied Biosystems) or using the following primers and probes for the N-gene, forward: GACCCCAAAATCAGCGAAAT; reverse: TCTGGTTACTGCCAGTTGAATCTG, probe: ACCCCGCATTACGTTTGGTGGACC, or the 5'-UTR, forward: ACTGTCGTTGACAGGACACG, reverse: AACACGGACGAAACCGTAAG, probe: CGTCTATCTTCTGCAGGCTG. Viral RNA was expressed as gene copy numbers per mg for lung tissue homogenates and per mL for nasal swabs, based on a standard included in the assay that was created by in vitro transcription of a synthetic DNA molecule containing the target region of the N gene or 5'-UTR.

1.9 Statistical Analysis

Data were analyzed with GraphPad Prism 9.0 and statistical significance was assigned when P values were < 0.05. All tests and values are indicated in figure legends.

Supplementary Methods. Protocol for "Phase 1 Study in Healthy Participants to Evaluate the Safety, Tolerability, and Pharmacokinetics of Single-Ascending and Multiple Doses of an Anti-Severe Acute Respiratory Syndrome Coronavirus 2 (SARS-CoV-2) Chicken Egg Antibody (IgY)"

Protocol Number: CVR001

A Phase 1 Study in Healthy Participants to Evaluate the Safety, Tolerability, and Pharmacokinetics of Single-Ascending and Multiple Doses of an Anti-Severe Acute Respiratory Syndrome Coronavirus 2 (SARS-CoV-2) Chicken Egg Antibody (IgY)

Investigational Drug: Anti-SARS-CoV-2 IgY

Sponsor:
SPARK at Stanford

Sponsor Contact:
Daria Mochly-Rosen, PhD
Professor, Dept. of Chemical and Systems Biology
George D. Smith Professor of Translational Medicine
CCSR Room 3145a, 269 Campus Drive
Stanford University School of Medicine
Stanford, California 94305-5174
Tel: (650) 725-7720 (mobile)
E-mail: mochly@stanford.edu

Version 3.0, dated 29 September 2020

The information contained in this protocol is confidential and intended for the use of the study staff. The information in this document is the property of the sponsor and may not be disclosed unless federal or state law or regulations require such disclosure. Subject to the foregoing, this information may be disclosed only to those persons involved in the study who need to know, with the obligation not to further disseminate this information.

PROTOCOL SYNOPSIS

Protocol Number: CVR001

Title: A Phase 1 Study in Healthy Participants to Evaluate the Safety, Tolerability, and Pharmacokinetics of Single -Ascending and Multiple Doses of an Anti-Severe Acute Respiratory Syndrome Coronavirus 2 (SARS-CoV-2) Chicken Egg Antibody (IgY)

Objectives:

Part 1: Single Ascending Dose

The primary objective of Part 1 is to assess the safety and tolerability of anti-SARS-CoV-2 IgY when given as single ascending doses administered intranasally to healthy participants.

Part 2: Multiple Dose

The primary objective of Part 2 is to assess the safety and tolerability of anti-SARS-CoV-2 IgY when given as multiple doses administered intranasally in healthy participants. A secondary objective is to assess the pharmacokinetics of anti-SARS-CoV-2 IgY when given as multiple doses administered intranasally to healthy participants.

Study Design:

This is a single-center, randomized, double-blind, placebo-controlled phase 1 study of anti-SARS-CoV-2 IgY given intranasally to healthy participants.

Part 1: Participants will be randomly assigned to receive a single dose of anti-SARS-CoV-2 IgY antibodies or placebo in a sequential escalating manner. It is planned that up to 3 groups will be dosed with 8 healthy participants per group (6 active and 2 placebo in each group). Each group in Part 1 will include the initial dosing of a sentinel group (1 anti-SARS-CoV-2 IgY and 1 placebo) at least 24 hours prior to dosing the remaining 6 subjects in the cohort (5 anti-SARS-CoV-2 IgY and 1 placebo). The remainder of the cohort will only be dosed if, in the opinion of the investigator, there are no significant safety concerns identified in the sentinel subjects within the first 24 hours after administration of the dose (anti-SARS-CoV-2 IgY or placebo). The following regimens are planned:

- Regimen A: 2 mg anti-SARS-CoV-2 IgY preparation (2 drops of 0.5 mg/100 µL/drop to each nostril) or placebo.
- Regimen B: 4 mg anti-SARS-CoV-2 IgY preparation (2 drops of 1 mg/100 µL/drop to each nostril) or placebo.
- Regimen C: 8 mg anti-SARS-CoV-2 IgY preparation (2 drops of 2 mg/100 µL/drop to each nostril) or placebo.

Part 2: Participants will be randomly assigned to receive multiple daily administrations of anti-SARS-CoV-2 IgY or placebo every 4 hours (3-times daily) for 14 days in a parallel-group manner. It is planned that up to 24 healthy participants will be randomised to 1 of 4 treatment regimens (6 participants per regimen). The following regimens are planned:

- Regimen A: 6 mg total daily dose anti-SARS-CoV-2 IgY preparation (2 drops of 0.5 mg/100 µL/drop to each nostril 3-times daily) for 14 days
- Regimen B: 12 mg total daily dose anti-SARS-CoV-2 IgY preparation (2 drops of 1 mg/100 µL/drop to each nostril 3-times daily) for 14 days.
- Regimen C: 24 mg total daily dose anti-SARS-CoV-2 IgY preparation (2 drops of 2 mg/100 µL/drop to each nostril) for 14 days.
- Regimen D: 0 mg total daily dose placebo preparation (2 drops of 0 mg/100 µL/drop to each nostril) for 14 days.

Summary of Participant Eligibility Criteria:

Inclusion Criteria:

- Healthy males or non-pregnant, non-lactating females aged 18 to 45 years.
- Body weight of at least 50 kg.
- Body mass index ≥ 18.0 and ≤32.0 kg/m^2. If outside this range, eligible if not considered clinically significant.
- Good state of health (mentally and physically) in the opinion of the investigator as indicated by a comprehensive clinical assessment (detailed medical history and a complete physical examination), electrocardiogram (ECG), and laboratory investigations (hematology, clinical chemistry, coagulation, and urinalysis).
- Female participants are eligible to participate if they are not pregnant, not breastfeeding, and at least 1 of the following conditions applies:
  - Not of childbearing potential, defined as surgically sterile (hysterectomy, bilateral salpingectomy, tubal ligation or bilateral oophorectomy - verbal confirmation through medical history review acceptable) or postmenopausal (no menses for 12 months and confirmed by FSH level ≥40 mlU/mL);
  - Of childbearing potential and agrees to practice true abstinence or agrees to use a highly effective method of contraception consistently from 30 days prior to Day 1 until at least 30 days after dosing. Highly effective contraception includes hormonal contraception (oral, injected, implanted or transdermal) plus use of a condom, placement of an intrauterine device or intrauterine system plus use of a condom, or a vasectomized male partner (performed at least 6 months prior) who has been documented to no longer produce sperm - verbal confirmation through medical history review acceptable. Contraception requirements do not apply for participants in an exclusively same-sex relationship

- Male participants must agree to practice true abstinence; be surgically sterilized (performed at least 6 months prior and documented to no longer produce sperm - verbal confirmation through medical history review acceptable); or agree to use a condom plus effective contraception (i.e. established use of hormonal contraception - started at least 30 days prior to Day 1; or placement or an intrauterine device or intrauterine system) for their female partner, if of childbearing potential, from screening and for at least 90 days after dosing and refrain from donating sperm during this period. Contraception requirements do not apply for participants in an exclusively same-sex relationship
- Must provide written informed consent before any study procedure is performed

Exclusion Criteria:

- Participants who have received any investigational drug in a clinical research study within the previous 30 days prior to screening or 5 half-lives, whichever is longer.
- Participants who are study site employees, or immediate family members of a study site or sponsor employee.
- History of any drug or alcohol abuse in the past 2 years defined as >21 units of alcohol per week for males and >14 units of alcohol per week for females. Where 1 unit = 360 mL of beer, 150 mL wine, or 45 mL of spirits.
- Current smokers or users of e-cigarettes and nicotine replacement products and those who have used more than 3 of these products per month within the last 6 months.
- Females of childbearing potential who are pregnant or lactating or planning to become pregnant during the study (female participants of childbearing potential must have a negative pregnancy test at screening and Day 1). A woman is considered of childbearing potential unless she is permanently sterile (hysterectomy, bilateral salpingectomy and bilateral oophorectomy - verbal confirmation through medical history review acceptable) or is postmenopausal (had no menses for 12 months without an alternative medical cause and a serum follicle-stimulating hormone [FSH] concentration ≥40 IU/L).
- Participants who do not have suitable veins for multiple venipunctures/cannulation as assessed by the investigator at screening.
- Clinically significant abnormal biochemistry, hematology, coagulation, or urinalysis as judged by the investigator.
- Positive drugs of abuse test result.
- Evidence of renal impairment at screening, as indicated by an estimated creatinine clearance of <80 mL/min using the Cockcroft-Gault equation.

- Abnormal liver function tests as indicated by alanine aminotransferase (ALT) > 1.5 x upper limit of normal (ULN), aspartate aminotransferase (AST) > 1.5 x ULN or total bilirubin >1.5 x ULN. Note: may be repeated once at the discretion of the investigator. Participants with Gilbert's Syndrome may be enrolled at the discretion of the investigator.
- Positive test result for hepatitis B, hepatitis C or Human Immunodeficiency Virus (HIV) at Screening
- Positive test result for SARS-CoV-2 by RT-PCR or positive SARS-CoV-2 serology result at Screening.
- Evidence or history of clinically significant allergic (except for untreated, asymptomatic, seasonal allergies at time of dosing), hematological, endocrine, pulmonary, gastrointestinal, cardiovascular, hepatic, renal, psychiatric, or neurological disease.
- Participants with a history of egg allergy.
- History of nasal surgical procedures (e.g., turbinectomy, rhinoplasty, etc).
- Frequent or recurrent nasal conditions, as determined by the investigator, or any nasal conditions within the 7 days prior to Day 1 (e..g., rhinitis, hay fever).
- Current use of any nasal preparations (e.g. decongestants, steroids, etc).
- Acute illness (gastrointestinal, infection [e.g., influenza] or known inflammatory process) at screening or Day 1.
- Abnormal screening ECG, including QT intervals corrected with Fridericia's formula >450 msec for males or >470 msec for females, or subject has any cardiac rhythm other than sinus rhythm that is interpreted by the investigator to be clinically significant.
- Supine resting bradycardia (pulse heart rate [HR] <40 bpm) or a supine resting tachycardia (HR >100 bpm) at screening or Day 1.
- Hypertension, defined as a supine resting systolic blood pressure >140 mm Hg or a supine resting diastolic blood pressure >90 mm Hg at screening or Day 1.
- Hypotension, defined as a supine resting systolic blood pressure <90 mm Hg or a supine resting diastolic blood pressure <40 mm Hg at screening or Day 1.
- Known personal or family history of congenital long QT syndrome or known family history of sudden death.
- Any condition possibly affecting drug absorption or elimination, such as previous surgery on the nose.
- Donation or loss of greater than 400 mL of blood within the previous 3 months from screening.

- Taking, or have taken, any prescribed or over-the-counter drug (other than acetaminophen, or hormone replacement therapy and hormonal contraception) or herbal remedies in the 14 days before screening. Exceptions may apply on a case-by-case basis, if considered not to interfere with the objectives of the study, as agreed by the principal investigator and sponsor's medical monitor.
- Failure to satisfy the investigator of fitness to participate for any other reason.

Investigational Drug:
anti-SARS-CoV-2 IgY

Study Drug Doses and Route of Administration:

- Anti-SARS-CoV-2 IgY or placebo given intranasally. Study drug will be provided as drops from a bottle. Part 1 will include single ascending doses of anti-SARS-CoV-2 IgY preparation 2 mg, 4 mg, and 8 mg. Part 2 will include multiple doses of anti-SARS-CoV-2 IgY preparation 2 mg, 4 mg, and 8 mg every 4 hours while awake (3-times daily) for 14 days.

Safety Assessments:

- Clinical chemistry, hematology, coagulation, and urinalysis.
- Vital signs (blood pressure, heart rate, oral temperature, respiratory rate).
- Electrocardiogram.
- Physical examination.
- Adverse events.

Pharmacokinetic Assessment:

- Serum anti-SARS-CoV-2 IgY concentration.

Criteria for Dose Escalation:

- Progression to the next dose group in Parts 1 will be permitted after review of safety data suggests that it is safe to do so. For dose escalation to proceed, data must be available from a minimum of 7 participants who have completed the planned safety assessments up to 48 h after dosing to ensure that at least 5 participants in Part 1 received active study drug.
- The following data are required for interim study decisions: adverse events (mandatory), vital signs, safety laboratory values, ECG, and physical examinations.
- Dose escalation will not occur with any of the following:
  - a serious adverse reaction (i.e., a serious adverse event considered at least possibly related to the active drug) in one subject.
  - severe non-serious adverse reactions (i.e., severe non-serious adverse event considered at least possibly related to the active drug administration) in two participants in the same cohort, independent of within or not within the same system organ class.

Sample Size, Power, and Number of Sites:

- No formal sample size calculation was done. Based on experience from previous similar studies, the target number of participants to be enrolled is appropriate for the assessment of safety and tolerability.
- In Part 1, 24 healthy participants will be enrolled into 3 groups, each of 8 participants. In Part 2, 24 healthy participants will be enrolled into 4 parallel treatment arms of 6 participants each.
- In both Parts 1 and 2, up to 2 replacement participants may be enrolled per group. Participants withdrawn due to an adverse event related to the anti-SARS-CoV-2 IgY will be considered evaluable and will not be replaced.
- Planned number of sites: one (Australia).

Removal of Participants from Study Drug or Assessment:

A participant must prematurely discontinue study drug under any of the following circumstances:

- The participant wishes to discontinue study drug.
- The investigator wishes the participant to discontinue study drug, especially but not limited to the investigator concluding that further treatment puts the participant at unacceptable risk.
- The participant develops a condition or begins a therapy that would have excluded entry into the study.
- The participant becomes pregnant during the study period. In this circumstance, the pregnancy must be immediately reported by telephone to the sponsor.
- The participant develops an adverse event with the Division of AIDS (DAIDS) Table for Grading the Severity of Adult and Pediatric Adverse Events that is grade 3 or greater toxicity and related to study drug in the judgment of the investigator.

A participant who discontinues study drug should complete all scheduled study visits *provided that written consent to do so has not been withdrawn*.

A participant must be withdrawn from the study (and discontinue any study drug) under the following circumstances:

- The participant requests discontinuation from the study.

Statistical Analysis:

Safety will be assessed on an intent-to-treat basis for all participants who have received study drug. Data will be summarized using descriptive statistics (number of participants, mean, median, standard deviation, minimum, and maximum) for continuous variables and frequency and percentages for categorical variables. Where confidence intervals are presented, they will be two-sided 95% confidence intervals.

Sponsor: SPARK at Stanford

TABLE OF CONTENTS

1. LIST OF ABBREVIATIONS 10
2. INTRODUCTION 11
2.1 Severe Acute Respiratory Syndrome Coronavirus 2 (SARS-CoV-2) Infection 11
2.2 Immunoglobulin Y 11
2.3 Use of anti-SARS-CoV-2 IgY for Prevention of SARS-CoV-2 Infection 12
3. OBJECTIVES 14
4. INVESTIGATIONAL PLAN 15
4.1 Summary of Study Design 15
4.2 Outline of Visit Schedule 16
4.2.1 Screening Evaluations 16
4.2.1.1 Part 1: Single-Ascending Dose Schedule of Events 17
4.2.1.2 Part 2: Multiple Dose Schedule of Events 19
5 DISCUSSION OF DESIGN 22
6 STUDY POPULATION 23
6.1 Inclusion & Exclusion Criteria 23
6.2 Removal of Participants from Study Drug or Assessment 26
6.2.1 Early Discontinuation of Study Drug 26
6.2.2 Participant Withdrawal from the Study 26
7 TREATMENTS 27
7.1 Participant Assignment 27
7.2 Method of Assignment to Treatment 27
7.3 Materials and Supplies 27
7.3.1 Formulation, Packaging and Labeling 27
7.3.2 Storage and Handling 29
7.3.3 Final Disposition of Clinical Supplies 29
7.4 Dosage Administration 29
7.5 Blinding 30
7.6 Concomitant Therapy 30
7.7 Dosing Diary 30
8 ADVERSE EVENT REPORTING 31
8.1 Definition of Adverse Event 31

8.1.1 Reporting Procedures for All Adverse Events ... 31
8.1.2 Adverse Event Severity ... 32
8.1.3 Adverse Event Relationship to Study Drug ... 32
8.1.4 Serious Adverse Event Definition and Reporting Procedures ... 33
8.1.5 Laboratory Tests ... 34
8.1.6 Safety Monitoring ... 34
9 QUALITY CONTROL AND QUALITY ASSURANCE ... 35
10 DATA ANALYSIS METHODS ... 36
10.1 Determination of Sample Size ... 36
10.2 Safety Variables ... 36
10.3 Pharmacokinetic Variable ... 36
10.4 Immunoglobulin E ... 36
10.5 Statistical and Analytical Plans ... 36
10.5.1 General Considerations ... 36
10.5.2 Handling of Missing Data ... 37
10.5.3 Participant Disposition ... 37
10.5.4 Participant Characteristics ... 37
10.5.5 Treatment Compliance ... 37
10.5.6 Safety Analyses ... 37
10.5.7 Pharmacokinetic Analysis ... 38
10.5.8 Interim Analyses ... 38
11 ADMINISTRATIVE, ETHICAL, AND REGULATORY CONSIDERATIONS ... 39
11.1 Ethical Review ... 39
11.2 Regulatory Considerations ... 39
11.2.1 Investigator Information ... 39
11.2.2 Protocol Amendments and Study Termination ... 40
11.2.3 Study Documentation, Privacy, and Records Retention ... 40
11.3 Study Finances ... 40
11.4 Publications ... 40
12 REFERENCES ... 41
APPENDIX A: Sponsor Protocol Approval ... 46

1. LIST OF ABBREVIATIONS

ABBREVIATION	DEFINITION
ACE2	angiotensin-converting enzyme 2
AE	adverse event
ALT	alanine transaminase
AST	aspartate transaminase
BUN	blood urea nitrogen
CLIA	Clinical Laboratory Improvement Amendments
COVID-19	coronavirus disease 2019
CRF	case report form
DAIDS	Division of AIDS
EC	Ethics Committee
FSH	follicle-stimulating hormone
hCG	human chorionic gonadotropin
HIPAA	Health Insurance Portability and Accountability Act
HIV	human immunodeficiency virus
IgG	immunoglobulin G
IgM	immunoglobulin M
IgY	immunoglobulin Y
INR	international normalized ratio
ITT	intent-to-treat
MedDRA	Medical Dictionary for Regulatory Activities
mg	milligram
mL	milliliter
µL	microliter
PT	prothrombin time
aPTT	activated partial thromboplastin time
rt-PCR	reverse transcription-polymerase chain reaction
SAE	serious adverse event
SARS-CoV-2	severe acute respiratory syndrome coronavirus 2
SDS-PAGE	sodium dodecyl sulfate-polyacrylamide gel electrophoresis
SMC	Safety Monitoring Committee
WHO	World Health Organization

2. INTRODUCTION

2.1 Severe Acute Respiratory Syndrome Coronavirus 2 (SARS-CoV-2) Infection

Coronaviruses are a large family of single-stranded RNA viruses that infect mammals and birds, causing respiratory infection. In December 2019, a novel coronavirus was identified as the cause of a cluster of pneumonia cases in Wuhan, China. Its rapid spread resulted in an epidemic throughout China that was followed by a dramatically increased number of cases worldwide. In February 2020, the World Health Organization (WHO) designated COVID-19 (coronavirus disease 2019) to be caused by a novel coronavirus termed severe acute respiratory syndrome coronavirus 2 (SARS-CoV-2) (World Health Organization 2020). As of August 3, 2020, over 18 million persons with COVID-19 had been identified in 215 countries with an estimated 692,000 deaths (World Health Organization 2020). The progression from isolated cases in China in late 2019 to the current worldwide pandemic is underscored by asymptomatic (Chan et al., 2020; Li et al., 2020; Mizumoto and Chowell, 2020) and presymptomatic (Arons et al., 2020; He et al., 2020; Kimball et al., 2020) infection and transmission, in addition to potential transmission through environmental contamination (Ong et al., 2020). Viral entry is most prominent through the nasal passage, where binding of the viral spike protein to the human angiotensin-converting enzyme 2 (ACE2) receptor facilitates cell entry (Sungank et al., 2020; Zhou et al., 2020). The ACE2 receptor is localized on mucosal epithelial cells (Hamming et al., 2004; Jia et al., 2006). There is currently no drug or vaccine that prevents or reduces the risk of acquiring SARS-CoV-2 infection.

2.2 Immunoglobulin Y

Antibody-based passive immunization with immunoglobulin G (IgG) has a long history of effectiveness in both preventing and treating human infectious diseases (Keller and Stiehm, 2000). The possibility of protective immunity following infection with SARS-CoV-2 has led to uncontrolled studies in a small number of critically ill hospitalized patients with COVID-19 that suggest potential benefit from the effect of convalescent plasma containing neutralizing antibodies (Duan et al., 2020; Salazar et al., 2020; Shen et al., 2020). As such, the potential of passive immunization with egg yolk antibodies against SARS-CoV-2 infection has also been raised (Constantin et al., 2020; Somasundaram et al., 2020).

Egg yolk antibodies, termed immunoglobulin Y (IgY), are present in birds, reptiles, and amphibians, and considered the evolutionary precursor of immunoglobulins such as IgG present only in mammals (Kovas-Nolan and Mine, 2012). Immunoglobulin Y does not cross-react with mammalian antibodies or other unintentional targets or activate the mammalian complement system or Fc receptors (Warr et al., 1995; Zhang et al., 2017).

Immunoglobulin Y has been beneficial with favorable safety and tolerability when given prophylactically in both animal models and clinical settings of viral diseases, including respiratory infections (Kovas-Nolan and Mine, 2012). For example, animal studies have

shown that the prophylactic treatment of calf and newborn piglets with orally administered IgY was highly effective and well tolerated against coronaviruses and rotavirus (Ikemori et al., 1997; Vega et al., 2011). Intranasal administration of IgY was beneficial and well tolerated in mouse models of influenza infection (Nguyen at al., 2010; Wallach et al., 2011; Yang et al., 2014) and *P. aeruginosa* acute pneumonia (Ranjbar et al., 2019), and a guinea pig model of allergic rhinitis (Guo-Zhu et al., 2015; Wei-Xu et al., 2016). Inhaled anti-*Pseudomonas* IgY were protective and well tolerated in a ventilated piglet model of *P. aeruginosa* infection (Otterbeck et al., 2019).

In humans, continuous prophylactic treatment by daily mouth rinse with specific IgY against *P. aeruginosa* in 17 patients with cystic fibrosis for up to 12 years (114 patient-years) significantly reduced pulmonary *P. aeruginosa* infections compared with 23 cystic fibrosis control patients, with no adverse events (Kollberg et al., 2003; Nilsson et al., 2008). A randomized, double-blind, placebo-controlled phase 3 trial of 164 patients with cystic fibrosis conducted at 47 European sites from 2011 to 2015 evaluated nightly treatment with a gargling solution of anti- *P. aeruginosa* IgY (Schuster et al., 2019). No clinically meaningful differences in adverse events were reported between the treatment arms. Additional clinical studies evaluating IgY given orally include (a) treatment with a gel containing anti-*Candida* IgY, which improved tongue coating and the number of *Candida* colonies (Takeuchi et al., 2016), (b) treatment with lozenges containing anti-*P. gingivalis* IgY, which improved gum pocket depth, gingival bleeding, and bacterial levels (Yokoyama et al., 2007; Nguyen et al., 2018), (c) ingestion of anti-rotavirus IgY, which reduced stool frequencies, viral excretion, and lowered hydration needs in children with rotavirus diarrhea (Sarker et al., 2001; Rahman et al., 2012), and (d) suppression of *H. pylori* after ingestion of yogurt containing anti-*H.pylori* IgY (Horie et al., 2004).

Overall, available data to date suggest that IgY given systemically do not have off-target inflammatory effects and are nontoxic to humans, thus permitting clinical applications in a wide range of vulnerable populations including the elderly, immunocompromised, and young children (Rahman et al., 2013).

2.3 Use of anti-SARS-CoV-2 IgY for Prevention of SARS-CoV-2 Infection

There are no published studies that have evaluated the use of anti-SARS-CoV-2 IgY to prevent human SARS-CoV-2 infection. Active vaccination requires the induction of an immune response that takes time to develop and varies depending on the vaccine and recipient. In contrast, passive antibody administration may provide immediate immunity to susceptible persons.

Passive immunization with sera from recovered patients infected with SARS-CoV-2 is currently under investigation and requires the availability of a population of donors who have recovered from the disease and can donate convalescent serum, blood banking facilities to

process the serum donations, availability of assays to detect SARS-CoV-2 in serum, virologic assays to measure viral neutralization, virology laboratory support to perform these assays, and clinical protocols that are well designed and comply with country-specific and local regulatory approval (Casadevall and Pirofski, 2020). Passive immunization with anti-SARS-CoV-2 IgY may offer advantages in the known safety profile of IgY, applicability to a wide array of individuals, high yield per egg, and rapid mass production at a low cost given the large production of eggs for human consumption (Constantin et al., 2020; Somasundaram et al., 2020). In addition, anti-SARS-CoV-2 IgY would not be expected to elicit antibody-dependent enhancement of infection due to an inability to bind to human IgG Fc receptors on immune cells.

Intranasal antibody prophylaxis is an especially effective means against multiple viral pathogens (Hemmingsson and Hammarström, 1993; Giraudi et al., 1997; Heikkinen et al., 1998; Weltzin and Monath, 1999). Further, key characteristics of the mechanism of action of anti-SARS-CoV-2 IgY may be ideal for effective immunization: a) anti-SARS-CoV-2 IgY may bind to the spike protein on the surface of the virus, competing with the binding of the viral spike protein to the human ACE2 receptor to prevent cell entry and infection, b) anti-SARS-CoV-2 IgY may also agglutinate SARS-CoV-2 on the surface of the mucosa, thus preventing viral entry across the mucosa, and c) intranasal administration can deliver anti-SARS-CoV-2 IgY to the nasal passage and throat mucosa (through mucociliary clearance), the main routes of viral entry and replication.

In the absence of an active vaccine, this approach may be especially valuable for immediate and short-lived protection when used with personal protective equipment and other preventative measures to provide added protection for frontline health care personnel or other service workers at increased risk of infection.

Neutralization test results showed that SARS-CoV-2 IgY was effective in neutralizing SARS-CoV-2 (SPARK at Stanford, data on file). More detailed information about nonclinical and preclinical studies can be found in the Investigator’s Brochure.

3. OBJECTIVES

Part 1: Single Ascending Dose

The primary objective of Part 1 is to assess the safety and tolerability of anti-SARS-CoV-2 IgY when given as single-ascending doses administered intranasally to healthy participants.

Part 2: Multiple Dose

The primary objective of Part 2 is to assess the safety and tolerability of anti-SARS-CoV-2 IgY when given as multiple doses administered intranasally to healthy participants. A secondary objective is to assess the pharmacokinetics of anti-SARS-CoV-2 IgY when given as multiple doses administered intranasally to healthy participants.

Safety will be evaluated using adverse event (AE), physical examination (including vital signs), electrocardiogram, and clinical laboratory data. Pharmacokinetics will be evaluated by serum anti-SARS-CoV-2 IgY concentration.

4. INVESTIGATIONAL PLAN

4.1 Summary of Study Design

This is a single-center, randomized, double-blind, placebo-controlled phase 1 study of anti-SARS-CoV-2 IgY given intranasally to healthy participants at a single site in Australia. Participants will be recruited from the Linear Clinical Research Ltd panel or by direct advertising to the public.

Part 1: Participants will be randomly assigned to receive a single dose of anti-SARS CoV-2 IgY or placebo in a sequential escalating manner. It is planned that up to 3 groups will be dosed with 8 healthy participants per group (6 active and 2 placebo in each group). Each group in Part 1 will include the initial dosing of a sentinel group (1 anti-SARS-CoV-2 IgY and 1 placebo) at least 24 hours prior to dosing the remaining 6 subjects in the cohort (5 anti-SARS-CoV-2 IgY and 1 placebo). The remainder of the cohort will only be dosed if, in the opinion of the investigator, there are no significant safety concerns identified in the sentinel subjects within the first 24 hours after administration of the dose (anti-SARS-CoV-2 IgY or placebo). The following regimens are planned:

- Regimen A: 2 mg anti-SARS-CoV-2 IgY preparation (2 drops of 0.5 mg/100 µL/drop to each nostril) or placebo.
- Regimen B: 4 mg anti-SARS-CoV-2 IgY preparation (2 drops of 1 mg/100 µL/drop to each nostril) or placebo.
- Regimen C: 8 mg anti-SARS-CoV-2 IgY preparation (2 drops of 2 mg/100 µL/drop to each nostril) or placebo.

Part 2: Participants will be randomly assigned to receive multiple daily administrations of anti-SARS-CoV-2 IgY or placebo every 4 hours (3-times daily) for 14 days in a parallel-group manner. It is planned that up to 24 healthy participants will be randomised to 1 of 4 treatment regimens (6 participants per regimen). The following regimens are planned:

- Regimen A: 6 mg total daily dose anti-SARS-CoV-2 IgY preparation (2 drops of 0.5 mg/100 µL/drop to each nostril 3-times daily) for 14 days.
- Regimen B: 12 mg total daily dose anti-SARS-CoV-2 IgY preparation (2 drops of 1 mg/100 µL/drop to each nostril 3-times daily) for 14 days.
- Regimen C: 24 mg total daily dose anti-SARS-CoV-2 IgY preparation (2 drops of 2 mg/100 µL/drop to each nostril) for 14 days.
- Regimen D: 0 mg total daily dose placebo preparation (2 drops of 0 mg/100 µL/drop to each nostril) for 14 days

4.2 Outline of Visit Schedule

4.2.1 Screening Evaluations

Screening assessments must be done to determine participant eligibility. Written consent must be obtained before conducting any study procedures.

4.2.1.1 Part 1: Single-Ascending Dose Schedule of Events

**Part 1: Single-Ascending Dose Schedule of Events**										
**Study Assessment**	**Screening**	**Treatment Period**						**Phone Visit**	**Phone Visit**	**EOS Visit**
**Assessment**	**Day -28 to -1**	**Day 1**						**Day 2[11]**	**Day 3**	**Day 8**
		**Pre-dose**	**0 hrs**	**30 min**	**1 hr**	**1.5 hrs**	**2 hrs**	**24 hrs**	**N/A**	**N/A**
**Visit Windows**	**NA**	**-2 hrs**	**NA**	**(± 10 min)**	**(± 15 min)**	**(± 15 min)**	**(± 15 min)**		**(+ 1 day)**	**(+ 1 day)**
Visit to study site[1]	**X**	X								**X**
Informed consent[2]	X									
Inclusion/exclusion criteria	X	X[3]								
Demographic data	X									
Medical history	X	X								
Prior medication history	X	X								
Vein assessment	X									
Physical examination[4]	X						X (abbrev)			X (abbrev)
Height	X									
Weight	X	X								
Vital signs[5]	X	X		X	X	X	X			X
12-lead electrocardiogram[6]	X	X					X			
Randomization		X								
Investigational Product			X							
Adverse event reporting			X *(continuous reporting)*					X	X	X
Concomitant medications			X *(continuous reporting)*					X	X	X
Alcohol breath test	X	X								
Drugs of abuse screen	X	X								
Pregnancy screen[7]	X	X								
FSH[8]	X									

Part 1: Single-Ascending Dose Schedule of Events										
**Study Assessment**	**Screening**	**Treatment Period**						**Phone Visit**	**Phone Visit**	**EOS Visit**
**Assessment**	**Day -28 to -1**	**Day 1**						**Day 2[11]**	**Day 3**	**Day 8**
		**Pre-dose**	**0 hrs**	**30 min**	**1 hr**	**1.5 hrs**	**2 hrs**	**24 hrs**	**N/A**	**N/A**
**Visit Windows**	**NA**	**-2 hrs**	**NA**	**(± 10 min)**	**(± 15 min)**	**(± 15 min)**	**(± 15 min)**		**(+ 1 day)**	**(+ 1 day)**
Serology[9]	X									
Clinical Safety Labs[10]	X						X			X
SARS-CoV-2 Test	X									
IgE Test[12]	X									

Footnotes

1 Participants will be required to attend the study site on the morning of Day 1 and will be confined to the study site until after collection of the 2-hour post-dose assessments;
2 Informed consent will be obtained prior to any study procedures being performed;
3 Pre-dose on Day 1 continued eligibility following the screening visit will be confirmed prior to dosing;
4 Physical examination will include examination of: general appearance, head, ears, eyes, nose, throat, neck (including thyroid), skin, cardiovascular system, respiratory system, gastrointestinal system, musculoskeletal system, lymph nodes and nervous system. Abbreviated exam will include at a minimum assessment of general appearance, head, nose, throat, respiratory system and cardiovascular system.
5 Vital signs include blood pressure, heart rate, respiratory rate and oral temperature and will be collected following a minimum 5 minutes rest in a seated or supine position;
6 ECGs will be collected following a minimum 5 minutes rest in a supine position;
7 Females of childbearing potential will be screened for pregnancy. The screening pregnancy test will be by blood sample to detect the presence of β-HCG (collected as part of clinical chemistry sample). Pre-dose on Day 1, pregnancy will be determined by urine dipstick.
8 Only for women of postmenopausal status.
9 Serology screening will include tests for tests hepatitis B surface antigen, hepatitis C virus and human immunodeficiency virus;
10 Clinical safety labs will be collected non-fasted and include:

- Haematology: hemoglobin, hematocrit, erythrocyte count (RBC), mean cell volume (MCV), neutrophils, lymphocytes, monocytes, eosinophils, basophils, platelets, reticulocyte count;
- Clinical chemistry: Serum concentrations of sodium, potassium, chloride, total bilirubin, indirect bilirubin (screening only), direct bilirubin (screening only), alkaline phosphatase, alanine transaminase (ALT/SGPT), aspartate transaminase (AST/SGOT), blood urea nitrogen (BUN), creatinine, uric acid, phosphorous, calcium, plasma glucose, total protein, albumin, cholesterol, creatinine kinase (CK), creatinine clearance (screening only);
- Coagulation: prothrombin time (PT), activated partial thromboplastin time (aPTT), international normalized ratio (INR); and
- Urinalysis: specific gravity, pH, protein, glucose, blood, nitrites, leukocyte esterase. Microscopy will be performed if abnormalities noted for protein, blood or leukocytes, or if clinically indicated.

11 Follow-up phone call 24 hours post-dose for sentinel participants only, to confirm dosing in rest of cohort.
12 IgE sample is not required as an entry criterion and may be collected any time prior to dosing on Day 1.

4.2.1.2 Multiple Dose Schedule of Events

**Part 2: Multiple Dose Schedule of Events**																						
**Study Assessment**	**Screening**			**Treatment Period**																	**F/U Visit**	**EOS Visit**
**Assessment**	**Day -28 to -1**	**Day 1**						**Day 2-4**			**Day 5-6**	**Day 7 (+1 day)[14]**		**Day 8-13**	**Day 14**						**Day 16**	**Day 21**
		**Pre-dose**	**0 hrs**	**30 min**	**1 hr**	**1.5 hrs**	**2 hrs**	**Pre-dose**	**0 hrs**	**2 hrs**		**Pre-dose**	**0 hrs**		**Pre-dose**	**0 hrs**	**30 min**	**1 hr**	**1.5 hrs**	**2 hrs**	**N/A**	**N/A**
**Visit Windows**	**NA**	**-2 hrs**	**NA**	**± 10 min**	**± 15 min**	**± 15 min**	**± 15 min**	**-2 hrs**	**NA**	**± 15 min**		**-2 hrs**	**NA**		**-2 hrs**	**NA**	**± 10 min**	**± 15 min**	**± 15 min**	**± 15 min**	**+ 1 day**	**+ 1 day**
Visit to study site[1]	X	X						X				X			X						X	X
Informed consent[2]	X																					
Inclusion/exclusion criteria	X	X[3]																				
Demographic data	X																					
Medical history	X	X																				
Prior medication history	X	X																				
Vein assessment	X																					
Physical examination[4]	X						X (abb)			X (abb)										X (abb)		X (abb)
Height	X																					
Weight	X	X						X				X			X							
Vital signs[5]	X	X		X	X	X	X	X		X		X			X		X	X	X	X	X	X
12-lead electrocardiogram[6]	X	X					X	X[7]							X					X		
Randomization		X																				
Administer Study Drug			X						X		X		X	X		X						

Part 2: Multiple Dose Schedule of Events																						
**Study Assessment**	**Screening**			**Treatment Period**																	**F/U Visit**	**EOS Visit**
**Assessment**	**Day -28 to -1**	**Day 1**						**Day 2-4**			**Day 5-6**	**Day 7 (+1 day)[14]**		**Day 8-13**	**Day 14**						**Day 16**	**Day 21**
		**Pre-dose**	**0 hrs**	**30 min**	**1 hr**	**1.5 hrs**	**2 hrs**	**Pre-dose**	**0 hrs**	**2 hrs**		**Pre-dose**	**0 hrs**		**Pre-dose**	**0 hrs**	**30 min**	**1 hr**	**1.5 hrs**	**2 hrs**	**N/A**	**N/A**
**Visit Windows**	**NA**	**-2 hrs**	**NA**	**± 10 min**	**± 15 min**	**± 15 min**	**± 15 min**	**-2 hrs**	**NA**	**± 15 min**		**-2 hrs**	**NA**		**-2 hrs**	**NA**	**± 10 min**	**± 15 min**	**± 15 min**	**± 15 min**	**+ 1 day**	**+ 1 day**
Dispense Study Drug							X			X			X									
Dosing Diary Review								X				X			X							
Adverse event reporting			X *(continuous reporting)*																		X	X
Concomitant medications			X *(continuous reporting)*																		X	X
Alcohol breath test	X	X																				
Drugs of abuse screen	X	X																				
Pregnancy screen[8]	X	X													X							
FSH[9]	X																					
Serology[10]	X																					
Clinical Safety Labs[11]	X						X			X[12]										X		X
SARS-CoV-2 Test	X																					
IgE Test[13]	X																					
Serum anti-SARS-CoV-2 IgY concentration		X		X	X	X	X	X		X					X		X	X	X	X		

Footnotes

1 Participants will be required to attend the study site on the morning of Day 1-4, Day 7 and Day 14, and will be confined to the study site until after collection of the 2-hour post-dose assessments on Days 1-4 and 14. On Day 7, participants can be discharged following dosing;

2 Informed consent will be obtained prior to any study procedures being performed;

3 Pre-dose on Day 1 continued eligibility following the screening visit will be confirmed prior to dosing;

4 Physical examination will include examination of: general appearance, head, ears, eyes, nose, throat, neck (including thyroid), skin, cardiovascular system, respiratory system, gastrointestinal system, musculoskeletal system, lymph nodes and nervous system. Abbreviated exam will include at a minimum assessment of general appearance, head, nose, throat, respiratory system and cardiovascular system.

5 Vital signs include blood pressure, heart rate, respiratory rate, and oral temperature and will be collected following a minimum 5 minutes rest in a seated or supine position;

6 ECGs will be collected following a minimum 5 minutes rest in a supine position;

7 Day 4 only

8 Females of childbearing potential will be screened for pregnancy. The screening pregnancy test will be by blood sample to detect the presence of β-HCG (collected as part of clinical chemistry sample). Pre-dose on Day 1, pregnancy will be determined by urine dipstick.

9 Only for women of postmenopausal status.

10 Serology screening will include tests for tests hepatitis B surface antigen, hepatitis C virus and human immunodeficiency virus;

11 Clinical safety labs will be collected non-fasted and include:

- Haematology: hemoglobin, hematocrit, erythrocyte count (RBC), mean cell volume (MCV), neutrophils, lymphocytes, monocytes, eosinophils, basophils, platelets, reticulocyte count;
- Clinical chemistry: Serum concentrations of sodium, potassium, chloride, total bilirubin, indirect bilirubin (screening only), direct bilirubin (screening only), alkaline phosphatase, alanine transaminase (ALT/SGPT), aspartate transaminase (AST/SGOT), blood urea nitrogen (BUN), creatinine, uric acid, phosphorous, calcium, plasma glucose, total protein, albumin, cholesterol, creatinine kinase (CK), creatinine clearance (screening only);
- Coagulation: prothrombin time (PT), activated partial thromboplastin time (aPTT), international normalized ratio (INR); and
- Urinalysis: specific gravity, pH, protein, glucose, blood, nitrites, leukocyte esterase. Microscopy will be performed if abnormalities noted for protein, blood or leukocytes, or if clinically indicated.

12 Haematology, clinical chemistry and coagulation samples collected on Days 2 -4. Urinalysis only collected on Day 4

13 IgE sample is not required as an entry criterion and may be collected any time prior to dosing on Day 1.

14 Participants will attend the study site on the morning of Day 7 (or Day 8 if the +1 day window is utilized). On the day they do not attend the study site, all doses for that day will be administered at home.

5 DISCUSSION OF DESIGN

This is a single-center, randomized, double-blind, placebo-controlled phase 1 study of anti-SARS-CoV-2 IgY given intranasally to healthy participants.

Part 1: Participants will be randomly assigned to receive a single dose of anti-SARS-CoV-2 IgY or placebo in a sequential escalating manner. It is planned that up to 3 groups will be dosed with 8 healthy participants per group (6 active and 2 placebo in each group).

Part 2: Participants will be randomly assigned to receive multiple daily administrations of anti-SARS-CoV-2 IgY antibodies or placebo every 4 hours (3-times daily) for 14 days in a parallel-group manner. It is planned that up to 24 healthy participants will be randomised to 1 of 4 treatment regimens (6 participants per regimen).

Dose: The maximum feasible solubility of IgY is approximately 20 mg/mL. The total maximum daily dose of anti-SARS-CoV-2 IgY to be used in the present study (24 mg daily) is based in part on solubility considerations and is less than the daily dose of anti- *P. aeruginosa* IgY given prophylactically as continuous oral treatment for up to 12 years (114 patient-years) to prevent pulmonary infections in 17 patients with cystic fibrosis (Nilsson et al., 2008). This study showed a significant reduction in *P. aeruginosa* compared with 23 control patients with cystic fibrosis, with no adverse events (Nilsson et al., 2008). Further, an equivalent of ½ egg/treatment was used/dose, which translates to about 50 mg IgY/dose given once daily (Dr. Anders Larsson, Uppsala University, Sweden, personal communication). As a result, the maximum daily dose in the planned Phase 1 study of 24 mg given intranasally to healthy volunteers for 14 days is significantly less than the oral daily dose given continuously to patients with cystic fibrosis over long periods. For the maximum anti-SARS-CoV-2 IgY dose of 4 mg/nare, we calculate a favorable ratio of IgY to viral particles, even when viral amount covers 100% of the nasal pathway and 100% of the mucosa thickness.

Entry Criteria: Participants will be healthy adults and receive standard assessments to determine eligibility.

Safety Endpoints: The safety assessments of physical examination, vital signs, clinical laboratories, electrocardiogram, and adverse event collection used in this trial are standard assessments for phase 1 studies evaluating the safety of single and repeated dosing.

Pharmacokinetic Endpoint: Serum levels of anti-SARS-CoV-2 IgY will be measured by enzyme-linked immunosorbent assay to determine the presence of systemic exposure.

6 STUDY POPULATION

The investigators participating in this study have expertise in the conduct of phase 1 studies. The participants will comprise healthy adults between the ages of 18 and 45.

Participation in this study is voluntary. The nature of the study will be fully explained to each participant during the informed consent. The participants will have the opportunity to ask questions. An informed consent document will then be signed by the participant and the person performing the consent discussion and retained by the investigator according to Good Clinical Practice. A copy of the signed informed consent document will be given to the participant.

Eligibility for enrollment will be based on the results of screening for the following inclusion and exclusion criteria.

6.1 Inclusion & Exclusion Criteria

Inclusion Criteria

1) Healthy males or non-pregnant, non-lactating females aged 18 to 45 years.
2) Body weight of at least 50 kg.
3) Body mass index between ≥18.0 and ≤32.0 kg/m^2. If outside this range, eligible if not considered clinically significant.
4) Good state of health (mentally and physically) in the opinion of the investigator as indicated by a comprehensive clinical assessment (detailed medical history and a complete physical examination), electrocardiogram (ECG), and laboratory investigations (hematology, clinical chemistry, coagulation, and urinalysis).
5) Female participants are eligible to participate if they are not pregnant, not breastfeeding, and at least 1 of the following conditions applies:
   - Not of childbearing potential, defined as surgically sterile (hysterectomy, bilateral salpingectomy, tubal ligation or bilateral oophorectomy - verbal confirmation through medical history review acceptable) or postmenopausal (no menses for 12 months and confirmed by follicle-stimulating hormone (FSH) level ≥40 mlU/mL);
   - Of childbearing potential and agrees to practice true abstinence or agrees to use a highly effective method of contraception consistently from 30 days prior to Day 1 until at least 30 days after dosing. Highly effective contraception includes hormonal contraception (oral, injected, implanted or transdermal) plus use of a condom, placement of an intrauterine device or intrauterine system plus use of a condom, or a vasectomised male partner (performed at least 6 months prior) who has been documented to no longer produce sperm - verbal confirmation through medical history review acceptable. Contraception requirements do not apply for participants in an exclusively same-sex relationship

6) Male participants must agree to practice true abstinence; be surgically sterilised (performed at least 6 months prior and documented to no longer produce sperm - verbal confirmation through medical history review acceptable); or agree to use a condom plus effective contraception (i.e. established use of hormonal contraception - started at least 30 days prior to Day 1; or placement or an intrauterine device or intrauterine system) for their female partner, if of childbearing potential, from screening and for at least 90 days after dosing and refrain from donating sperm during this period. Contraception requirements do not apply for participants in an exclusively same-sex relationship
7) Must provide written informed consent before any study procedure is performed.

Exclusion Criteria:

1) Participants who have received any investigational drug in a clinical research study within the previous 30 days prior to screening or 5 half-lives, whichever is longer.
2) Participants who are study site employees, or immediate family members of a study site or sponsor employee.
3) History of any drug or alcohol abuse in the past 2 years defined as >21 units of alcohol per week for males and >14 units of alcohol per week for females. Where 1 unit = 360 mL of beer, 150 mL wine, or 45 mL of spirits.
4) Current smokers or users of e-cigarettes and nicotine replacement products and those who have used more than 3 of these products per month within the last 6 months.
5) Females of childbearing potential who are pregnant or lactating or planning to become pregnant during the study (female participants of childbearing potential must have a negative pregnancy test at screening and Day 1). A woman is considered of childbearing potential unless she is permanently sterile (hysterectomy, bilateral salpingectomy, and bilateral oophorectomy) or is postmenopausal (had no menses for 12 months without an alternative medical cause and a serum follicle-stimulating hormone [FSH] concentration ≥40 IU/L).
6) Participants who do not have suitable veins for multiple venipunctures/cannulation as assessed by the investigator at screening.
7) Clinically significant abnormal biochemistry, hematology, coagulation or urinalysis as judged by the investigator.
8) Positive drugs of abuse test result.
9) Evidence of renal impairment at screening, as indicated by an estimated creatinine clearance of <80 mL/min using the Cockcroft-Gault equation.

10) Abnormal liver function tests as indicated by alanine aminotransferase (ALT) > 1.5 x upper limit of normal (ULN), aspartate aminotransferase (AST) > 1.5 x ULN or total bilirubin >1.5 x ULN. Note: may be repeated once at the discretion of the investigator. Participants with Gilbert's Syndrome may be enrolled at the discretion of the investigator.
11) Positive test result for hepatitis B, hepatitis C or human immunodeficiency virus (HIV) at Screening
12) Positive test result for SARS-CoV-2 by RT-PCR or positive SARS-CoV-2 serology result at Screening.
13) Evidence or history of clinically significant allergic (except for untreated, asymptomatic, seasonal allergies at time of dosing), hematological, endocrine, pulmonary, gastrointestinal, cardiovascular, hepatic, renal, psychiatric, or neurological disease.
14) Participants with a history of egg allergy.
15) History of nasal surgical procedures (e.g., turbinectomy, rhinoplasty, etc).
16) Frequent or recurrent nasal conditions, as determined by the investigator, or any nasal conditions within the 7 days prior to Day 1 (e..g., rhinitis, hay fever).
17) Current use of any nasal preparations (e.g. decongestants, steroids, etc).
18) Acute illness (gastrointestinal, infection [e.g., influenza] or known inflammatory process) at screening or Day 1.
19) Abnormal screening ECG, including QT intervals corrected with Fridericia's formula >450 msec for males or >470 msec for females, or subject has any cardiac rhythm other than sinus rhythm that is interpreted by the investigator to be clinically significant.
20) Supine resting bradycardia (pulse heart rate [HR] <40 bpm) or a supine resting tachycardia (HR >100 bpm) at screening or Day 1.
21) Hypertension, defined as a supine resting systolic blood pressure >140 mm Hg or a supine resting diastolic blood pressure >90 mm Hg at screening or Day 1.
22) Hypotension, defined as a supine resting systolic blood pressure <90 mm Hg or a supine resting diastolic blood pressure <40 mm Hg at screening or Day 1
23) Known personal or family history of congenital long QT syndrome or known family history of sudden death.
24) Any condition possibly affecting drug absorption or elimination, such as previous surgery on the nose.
25) Donation or loss of greater than 400 mL of blood within the previous 3 months from screening.
26) Taking, or have taken, any prescribed or over-the-counter drug (other than acetaminophen, or hormone replacement therapy and hormonal contraception) or herbal remedies in the 14 days before screening. Exceptions may apply on a case-by-case basis, if considered not to interfere with the objectives of the study as agreed by the principal investigator and sponsor's medical monitor.
27) Failure to satisfy the investigator of fitness to participate for any other reason.

6.2 Removal of Participants from Study Drug or Assessment

6.2.1 Early Discontinuation of Study Drug

A participant must prematurely discontinue study drug under any of the following circumstances:

- The participant wishes to discontinue study drug.
- The investigator wishes the participant to discontinue study drug, especially but not limited to the investigator concluding that further treatment puts the participant at unacceptable risk.
- The participant develops a condition or begins a therapy that would have excluded entry into the study.
- The participant becomes pregnant during the study period. In this circumstance, the pregnancy must be immediately reported by telephone to the sponsor.
- The participant develops an adverse event with the Division of AIDS (DAIDS) Table for Grading the Severity of Adult and Pediatric Adverse Events that is grade 3 or greater toxicity and related to study drug in the judgment of the investigator.

6.2.2 Participant Withdrawal from the Study

A participant must be withdrawn from the study (and discontinue any study drug) if:

- the participant requests such study discontinuation.

The reason for study withdrawal must be recorded in the participant's case report form (CRF).

7 TREATMENTS

7.1 Participant Assignment

After signing the informed consent documents, participants who meet all eligibility criteria will be assigned to study drug assignment.

7.2 Method of Assignment to Treatment

Participants who have met all the inclusion criteria for enrollment and none of the exclusion criteria will be randomized to receive either anti-SARS-CoV-2 IgY or placebo, with dosage determined based on cohort assignment. Computer-generated random numbers will be used by the study statistician to generate the randomization allocation sequence. The randomization number assigned to the participant, which will be used to link the participant to the unique medication number for the bottle of study drug to be administered to the participant.

After signing the informed consent form, each participant will be given a screening number according to the screening order. On Day 1, following confirmation of eligibility, participants will be randomized to receive anti-SARS-CoV-2 IgY or placebo.

At the time of randomization, the participant will be assigned a unique randomization number, which will be allocated sequentially based on the pre-determined randomization schedule, and according to their chronological order of inclusion in the study. Confirmation of the treatment number allocated will be documented in the drug accountability records and recorded in the electronic case report form (eCRF).

Both the screening and randomization numbers will be used to identify the participant throughout the study period and on all study-related documentation.

7.3 Materials and Supplies

7.3.1 Formulation, Packaging and Labeling

Study drug (anti-SARS-CoV-2 IgY or placebo) will be administered to the participant in a (personal use) bottle with a dropper (Bravado Pharma, Inc, Lutz, FL) through the site pharmacy. The isolation of IgY from individual eggs was performed using the protocols similar to the one described by Akita and Nakai (1993) and Fu et al (2012). Briefly, egg white was separated from the egg yolk and discarded. The yolk was mixed vigorously with sterilized water and left standing overnight at 4 °C after a slight acidification step. The mixture was then centrifuged at 1200×g for 30 minutes at 4 °C, and the supernatant was lyophilized. The protein concentration in harvested eluate was determined according to Bradford protein assay. Each egg yolk produced ~35-40 mg of IgY-enriched preparation.

The IgY preparation was subjected to analysis by sodium dodecyl sulfate-polyacrylamide gel electrophoresis (SDS-PAGE) and Western blot. The isolated IgY had a high purity as

confirmed by SDS-PAGE and purified IgY had good biological activity as confirmed by Western blot. The activity of IgY in yolks diluted at up to 1:128,000 in phosphate buffer saline was assessed using an indirect enzyme-linked immunosorbent assay as described previously (Huang et al., 2005). After immunization of specific-pathogen-free chickens with SARS coronavirus antigen, there were no detectable antibodies against SARS coronavirus in the yolks of eggs laid in the first 3 weeks after the first immunization. The production of anti-SARS antibody in yolks was found to commence 2 weeks after the IgY was produced in serum.

Drug substance (IgY preparation) is formulated with an inactive excipient (2% Carboxymethylcellulose Sodium), at neutral pH at ~300mosm. Each bottle of drug product (anti-SARS-CoV-2 IgY preparation) and matched placebo will have liquid identical in appearance and smell.

Table 7.1 Study Drug Treatment and Packaging (Part 1, Single-Ascending Dose)

Treatment	Study Drug	Frequency	Packaging
anti-SARS-CoV-2 IgY	anti-SARS-CoV-2 IgY preparation in liquid (0.5 mg/100 µL/drop, 1 mg/100 µL/drop, and 2 mg/100 µL/drop)	2 drops to each nostril as a single administration	Nose drop bottle
Placebo	liquid (100 µL/drop)	2 drops to each nostril 3-times daily while awake (every 4 hours)	Nose drop bottle

Table 7.2 Study Drug Treatment and Packaging (Part 2, Multiple Dose)

Treatment	Study Drug	Frequency	Packaging
anti-SARS-CoV-2 IgY	anti-SARS-CoV-2 IgY preparation in liquid (0.5 mg/100 µL/drop, 1 mg/100 µL/drop, and 2 mg/100 µL/drop)	2 drops to each nostril every 4 hours (3-times daily) for 14 days	Nose drop bottle
Placebo	liquid (100 µL/drop)	2 drops to each nostril every 4 hours (3-times daily) for 14 days	Nose drop bottle

Study drug bottle will contain the following information:

- Protocol number (CVR001).
- Dose: Each vial contains 1.5 mL anti-SARS-CoV-2 IgY Preparation Nasal Suspension, 5, 10, or 20 mg/mL, or Placebo, for Intranasal application.
- Lot number (BP0001).

- Retest Date.
- Storage: 2 ºC - 8 ºC (36 ºF - 46 ºF).
- A precautionary statement that drug is limited to clinical trial use only.
- Keep out of reach of children.
- Company name (SPARK).
- Manufacturer: Bravado Pharmaceuticals, LLC, 44212 Cypress Gulch Drive, Lutz, FL 33559.

7.3.2 Storage and Handling

The study drug will be in bottles. The study drug will be stored at 2 ºC - 8 ºC (36 ºF - 46 ºF). No other special handling is required.

7.3.3 Final Disposition of Clinical Supplies

At the end of the study, study drug supply and accountability records will be reconciled as to drug shipped, drug consumed, and drug remaining. Any discrepancies noted will be documented. Final drug accountability reconciliation will be performed at the visit occurring at the end of treatment or early discontinuation. Unused study drug will be destroyed.

7.4 Dosage Administration

The investigator (or designee) will administer a single dose of study drug in Part 1 and the morning dose on Days 1-4, Day 7 and Day 14 in Part 2. All other doses in Part 2 will be self-administered by the participant. Participants will be instructed with the following language:

"Gently blow your nose before using this drug. Then tilt your head back while sitting or lying down. After the study drug is administered, keep your head tilted for a few minutes. Try not to blow your nose for at least 5 minutes after study drug administration"

In Part 2, participants will be dispensed study drug in a dropper bottle to complete the subsequent doses at home, 4 hours apart for a total of 3 doses per day. They will retain the empty bottles to return to the site at the following visit for accountability. Doses performed at home will be recorded in a dosing diary. On Day 14 there will only be a single morning dose.

The investigator (or designee) is responsible for the correct use of the study drug to the participants, confirming that instructions are followed properly, maintaining accurate records of study drug dispensing, and collection of all unused study drug, including empty drug packaging.

Participants will be instructed to contact the investigator as soon as possible if he or she has a complaint or problem with the study drug or drug delivery system so that the situation can be assessed.

7.5 Blinding

This is a randomized, double-blind, placebo-controlled study. Neither the participants nor the study personnel with direct contact with the participant will know which study drug (anti-SARS-CoV-2 IgY vs. placebo) is being administered during the treatment period. Placebo and active drug liquid for intranasal administration will be similar in appearance and smell, and the amount of study drug will be the same for each treatment group.

Emergency unblinding for AEs will be performed through the code break envelopes. Emergency unblinding for AEs may be used only if a participant's medical care requires knowledge of the participant's treatment assignment. The investigator should make every effort to contact the sponsor before unblinding a participant's treatment assignment. If a participant's treatment assignment is unblinded, the sponsor must be immediately notified by telephone.

7.6 Concomitant Therapy

Nasal treatments that may interfere with administration of study drug in the judgment of the investigator are prohibited during the study.

7.7 Dosing Diary

Participants will be issued a dosing diary to record dosing times for treatment compliance. Participants will be instructed to bring their diary with them during all visits to the clinic.

8 ADVERSE EVENT REPORTING

8.1 Definition of Adverse Event

For purposes of this trial, an AE will be defined as **any** new unfavorable or unintended sign, symptom, or disease or change of an existing condition, which occurs during or after treatment, whether or not considered treatment-related. If clinically significant laboratory values lead to or are associated with clinical symptoms(s), the diagnosis should be reported as an adverse event. Lack of drug effect is not an adverse event in clinical trials because the purpose of the clinical trial is to establish drug effect.

Before enrollment, study site personnel will note the occurrence and nature of each participant's medical condition(s) in the appropriate section of the CRF. During the remainder of the study, site personnel will again note any change in the condition(s) and the occurrence and nature of any adverse events.

If the study drug is discontinued for a participant, study site personnel must report and clearly document the circumstances and data leading to any such discontinuation, using designated case report forms. For adverse events, the participant should be followed until the event resolves or stabilizes, with frequency of follow-up at the discretion of the investigator.

In cases where the investigator notices an unanticipated benefit to the participant, study site personnel should enter "unexpected benefit" with the actual event term (for example, the complete actual term would be "unexpected benefit—sleeping longer").

Cases of pregnancy that occur during maternal or paternal exposures to study drug should be reported for tracking purposes. Data on fetal outcome and breastfeeding are collected for regulatory reporting and drug safety evaluation.

8.1.1 Reporting Procedures for All Adverse Events

Investigators are responsible for monitoring the safety of participants who have entered this study and noting any event that seems unusual, even if this event may be considered an unanticipated benefit to the participant. The investigator is responsible for appropriate medical care of participants during the study.

The investigator remains responsible for following, through an appropriate health care option, adverse events that are serious or that caused the participant to discontinue the study. The participant should be followed until the event is resolved or explained. Frequency of follow-up is left to the discretion of the investigator.

Adverse event information will be collected through Day 8 (Part 1) and Day 21 (Part 2). Participants who discontinue study drug at any time will have adverse events collected through an additional 1 week, *provided consent to continue in the study has not been withdrawn*.

The investigator is responsible for assessing and recording all adverse experiences. Each AE will be recorded and classified for intensity, seriousness, and causality. All AEs either observed by the investigator or reported by the participant will be recorded regardless of causality. The investigator will follow the participant until an AE resolves or stabilizes.

8.1.2 Adverse Event Severity

The Division of AIDS (DAIDS) Table for Grading the Severity of Adult and Pediatric Adverse Events version 2.1 (July 2017) will be used to assess and grade AE severity, including laboratory abnormalities judged to be clinically significant. If the experience is not covered in the DAIDS criteria, the following guidelines should be used to grade severity:

- Mild (grade 1): Mild symptoms causing no or minimal interference with usual social and functional activities with intervention not indicated.
- Moderate (grade 2): Moderate symptoms causing greater than minimal interference with usual social and functional activities with intervention indicate.
- Severe (grade 3): Severe symptoms causing inability to perform usual social and functional activities with intervention or hospitalization indicated.
- Life-threatening (grade 4): Potentially life-threatening symptoms causing inability to perform basic self-care functions with intervention indicated to prevent permanent impairment, persistent disability, or death.

The term “severe” is a measure of intensity and a severe AE is not necessarily serious.

8.1.3 Adverse Event Relationship to Study Drug

The relationship of an AE to the study drug should be based on the judgment of the investigator and assessed using the following guidelines:

- Definitely: Previously known toxicity of agent; or an event that follows a reasonable temporal sequence from administration of the drug; that follows a known or expected response pattern to the suspected drug; that is confirmed by stopping or reducing the dosage of the drug; and that is not explained by any other reasonable hypothesis.
- Probably: An event that follows a reasonable temporal sequence from administration of the drug; that follows a known or expected response pattern to the suspected drug; that is confirmed by stopping or reducing the dosage of the drug; and that is unlikely to be explained by the known characteristics of the participant’s clinical state or by other interventions.
- Possibly: An event that follows a reasonable temporal sequence from administration of the drug; that follows a known or expected response pattern to that suspected drug; but that could readily have been produced by a number of other factors.

- Unrelated: An event that can be determined with certainty to have no relationship to the study drug.

8.1.4 Serious Adverse Event Definition and Reporting Procedures

Any AE that meets the definition of serious noted below and occurs in a participant during the course of the study must be reported to the sponsor by telephone within 24 hours of the investigator becoming aware of the event. In addition, a serious adverse event form (SAE) must be completed by the investigator and faxed to the study sponsor within 24 hours of the investigator becoming aware of the event. In addition, the site investigator must report SAEs to their local Ethics Committee (EC) in accordance with the EC's standard operating procedures and policies.

An SAE is defined as an adverse event that suggests a significant hazard or side effect, regardless of the relationship to study drug. An SAE includes, but may not be limited to, any event that:

- Results in death.
- Is life-threatening. This definition implies that the participant, in the view of the investigator, is at immediate risk of death from the event. It does not include an event that, had it occurred in a more serious form, might have caused death.
- Requires inpatient hospitalization or prolongs existing hospitalization.
- Results in persistent or significant disability/incapacity.
- Results in a congenital anomaly or birth defect. This serious criterion applies if a participant exposed to an investigational product gives birth to a child with a congenital anomaly or birth defect.

Medical and scientific judgment will be exercised in deciding whether classification of an AE as serious is appropriate in other situations, such as important medical events that may not be immediately life-threatening or result in death or hospitalization, but may jeopardize the participant or require intervention to prevent one of the outcomes listed in the definition above. These should also usually be considered serious. Examples of such events include allergic bronchospasm requiring intensive treatment in an emergency room or at home, blood dyscrasias or convulsions that do not result in inpatient hospitalization, or the development of drug dependence or abuse.

Serious adverse events occurring after a participant is discontinued from the study will only be reported if the investigator believes that the event may have been caused by the study drug or a protocol procedure.

For the purpose of expedited reporting to regulatory agencies, an investigator will be responsible for identifying any adverse event that is serious, unexpected, and believed to be related to study drug. An adverse event or suspected adverse reaction is considered

"unexpected" if it is not consistent with the risk information described in the general investigational plan or elsewhere in the current investigational new drug application. For example, under this definition, hepatic necrosis would be unexpected (by virtue of greater severity) if the investigator brochure or investigational drug application referred only to elevated hepatic enzymes or hepatitis. Similarly, cerebral thromboembolism and cerebral vasculitis would be unexpected (by virtue of greater specificity) if the investigator brochure or investigational drug application listed only "cerebral vascular accidents.

8.1.5 Laboratory Tests

Clinical laboratory tests will be performed at the times specified in the Study Schedule (see Section 4.2). All clinical laboratory assessments will be analyzed at the participating site's local laboratory. Investigators must document their review of each laboratory report by signing or initialing and dating each report.

8.1.6 Safety Monitoring

The sponsor or designee will monitor blinded safety data throughout the study. The sponsor (or designee) will review SAEs within time frames mandated by regulatory requirements and will review trends and adverse events at periodic intervals.

Prior to dose escalation in the SAD cohorts, the Safety Monitoring Committee (SMC) will review all available safety and tolerability data for a minimum of 7 participants who have completed the planned safety assessments up to 48 hours after dosing. The SMC will be composed of the independent medical monitor, principal investigator, and Sponsor's medical representative. Other members of the investigational team may join the SMC as deemed appropriate.

The data will be reviewed blinded, unless the SMC considers it necessary to unblind the data for safety concerns. Before breaking the code per standard procedures, the potential decisions and actions will be determined.

SMC decisions on dose escalation will be taken in consensus between the members of the Safety Monitoring Committee. If consensus cannot be reached, the investigator, who has the ultimate responsibility for the safety of participants, will make the final decision on whether to continue or stop the study. The SMC's decisions and their rationale will be documented.

9 QUALITY CONTROL AND QUALITY ASSURANCE

The investigator agrees to be responsible for implementing and maintaining quality control and quality assurance systems to ensure that trials are conducted and data are generated, documented, and reported in compliance with the protocol, accepted standards of Good Clinical Practice, and all applicable federal, state, and local laws, rules and regulations relating to the conduct of the clinical study.

The investigator also agrees to conduct the study in an efficient and diligent manner and in conformance with this protocol; generally accepted standard of Good Clinical Practice; and all applicable federal, state, and local laws, rules, and regulations relating to the conduct of the clinical study.

The investigator must allow study-related monitoring, audits, and inspection by the EC, sponsor (or designee), government regulatory agencies, and, if applicable, University compliance and quality assurance groups of all trial-related documents and procedures.

The investigator shall prepare and maintain accurate study documentation in compliance with Good Clinical Practice standards and applicable federal, state, and local laws, rules, and regulations.

10 DATA ANALYSIS METHODS

10.1 Determination of Sample Size

No formal sample size calculation was done. Based on experience from previous similar studies, the target number of participants to be enrolled is appropriate for the assessment of safety and tolerability.

10.2 Safety Variables

Safety evaluations will include collection of AE, physical examination, vital sign, electrocardiogram, and clinical laboratory data. Laboratory data will include evaluation of:

- Hematology: hemoglobin, hematocrit, erythrocyte count, mean cell volume, neutrophils, lymphocytes, monocytes, eosinophils, basophils, platelets, reticulocyte count.
- Metabolic panel: Serum concentrations of sodium, potassium, chloride, total bilirubin, alkaline phosphatase, alanine transaminase (ALT), aspartate transaminase (AST), blood urea nitrogen (BUN), creatinine, uric acid, phosphorous, calcium, plasma glucose, total protein, albumin, cholesterol, creatinine kinase.
- Coagulation: prothrombin time (PT), activated partial thromboplastin time (aPTT), international normalized ratio (INR).
- Urinalysis: specific gravity, pH, protein, glucose, blood, nitrites, leukocyte esterase. Microscopy will be performed if abnormalities noted for protein, blood or leukocytes, or if clinically indicated.
- Urine human chorionic gonadotropin (hCG) test.

10.3 Pharmacokinetic Variable

Serum anti-SARS-CoV-2 IgY concentration.

10.4 Immunoglobulin E

Immunoglobulin E (IgE) blood samples will be collected from each participant. Samples will be stored and may be used for future allergen specific IgE testing.

10.5 Statistical and Analytical Plans

10.5.1 General Considerations

The analysis of safety variables will include all participants who receive study drug.

All variables will be summarized by descriptive statistics for each treatment group. The statistics for continuous variables will include mean, median, standard deviation, and number

of observations. Categorical variables will be tabulated using frequencies and percentages. Where confidence intervals are presented, they will be two-sided 95% confidence intervals.

10.5.2 Handling of Missing Data

Missing data will not be imputed for safety analyses.

10.5.3 Participant Disposition

Study participant disposition will be summarized by treatment group. Participants who discontinued study drug prematurely or withdrew from the study will be summarized and listed, with reason for early termination/withdrawal.

10.5.4 Participant Characteristics

Demographic and other baseline characteristics will be summarized by treatment group.

10.5.5 Treatment Compliance

Treatment compliance will be evaluated. This will include the date the study drug was dispensed and the final amount of liquid returned.

10.5.6 Safety Analyses

Treatment-emergent AEs are defined as AEs occurring after the first dose of study drug until Day 8 in Part 1 and Day 21 in Part 2. Duration of treatment will be summarized by treatment group.

The incidence of all reported AEs and treatment-related AEs will be tabulated by treatment group. Adverse events will be classified by system organ class and preferred term using the Medical Dictionary for Regulatory Activities (MedDRA).

Adverse events will be listed and summarized by treatment group, MedDRA preferred term, severity, seriousness, and relationship to study drug. In the event of multiple occurrences of the same AE with the same preferred term in one participant, the AE will be counted once as the worst occurrence. The incidence of AEs will be tabulated by system organ class and treatment group. AEs leading to premature discontinuation of study drug or withdrawal from the study will be summarized and listed in the same manner.

Summary statistics for actual values and for change from baseline will be summarized for laboratory results by treatment group and scheduled visit. Participants with laboratory values outside of the normal reference range at any post baseline assessment will be identified.

Data to be listed by subject and summarized by treatment will include AEs, vital signs, ECG parameters, and clinical laboratory evaluations. All values outside the clinical reference ranges will be flagged on the data listings. Other data to be listed by subject will include physical examination findings and concomitant medications.

10.5.7 Pharmacokinetic Analysis

Summary statistics for actual values and for change from baseline will be summarized by treatment group and scheduled visit.

10.5.8 Interim Analyses

No interim analyses are planned for this study.

11 ADMINISTRATIVE, ETHICAL, AND REGULATORY CONSIDERATIONS

The investigator is responsible for presenting the risks and benefits of study participation to the participant in simple terms using the informed consent document. The investigator will ensure that written informed consent is obtained from each participant by obtaining the appropriate signatures and dates on the informed consent document before the performance of protocol evaluations or procedures.

11.1 Ethical Review

The investigator will obtain documentation of the EC approval of the protocol and the informed consent document before the study may begin at the investigative site(s). The name and address of the reviewing EC are provided in the investigator file.

The sponsor will supply the following to the investigative site's EC:

- Protocol and amendments.
- Informed consent document and updates.
- Investigational New Drug Application.
- Relevant curricula vitae, if required.
- Required safety and SAE reports.
- Any additional submissions required by the site's EC.

The investigator must provide the following documentation to the sponsor or its designee:

- The EC periodic (e.g., quarterly, annual) reapproval of the protocol.
- The EC approvals of any amendments to the protocol or revisions to the informed consent document.
- The EC receipt of safety and SAE reports, as appropriate.

11.2 Regulatory Considerations

This study will be conducted in accordance with the protocol and ethical principles stated in the 2013 version of the Declaration of Helsinki or the applicable guidelines on Good Clinical Practice, and all applicable federal, state, and local laws, rules, and regulations.

All data recorded in the CRF for participants participating in this study will be transcribed from medical records. After reading the protocol, the investigator will sign the protocol signature page and return it to the sponsor or designee.

11.2.1 Investigator Information

The contact information and qualifications of the principal investigator and sub-investigators, and name and address of the research facilities are included in the investigator file.

11.2.2 Protocol Amendments and Study Termination

The sponsor will initiate changes to the protocol as necessary (except for changes to eliminate an immediate hazard to a study participant) and seek approval by the EC before implementing. The investigator is responsible for enrolling participants who have met protocol eligibility criteria. Protocol violations must be reported to the local EC in accordance with EC policies.

The sponsor may terminate the study at any time. The EC must be advised in writing of study completion or early termination.

11.2.3 Study Documentation, Privacy, and Records Retention

Government agency regulations and directives require that all study documentation pertaining to the conduct of a clinical trial must be retained by the investigator for a minimum of 5 years (or longer if required by the local EC).

To protect the safety of participants in the study and to ensure accurate, complete, and reliable data, the investigator will keep records of laboratory tests, clinical notes, and participant medical records in the participant files as original source documents for the study. If requested, the investigator will provide the applicable regulatory agencies and applicable EC with direct access to original source documents.

Records containing participant medical information must be handled in accordance with the requirements of the Health Insurance Portability and Accountability Act (HIPAA) Privacy Rule and consistent with the terms of the participant authorization contained in the informed consent document for the study. Care should be taken to ensure that such records are not shared with any person or for any purpose not contemplated by the informed consent document. Furthermore, CRFs and other documents should be completed in strict accordance with the instructions provided by the sponsor, including the instructions regarding the coding of participant identities.

11.3 Study Finances

This study is financed through Linear Clinical Research Ltd and SPARK at Stanford.

11.4 Publications

Neither the complete nor any part of the results of the study carried out under this protocol, nor any of the information provided by the sponsor for the purposes of performing the study, will be published or passed on to any third party without the consent of the study sponsor. Any investigator involved with this study is obligated to provide the sponsor with complete test results and all data derived from the study.

12 REFERENCES

Akita EM, Nakai S (1993) Production and purification of Fab' fragments from chicken egg yolk immunoglobulin Y (IgY). *J Immunol Methods* 162(2):155-164.

Arons MM, Hatfield KM, Reddy SC, et al. (2020) Presymptomatic SARS-CoV-2 Infections and Transmission in a Skilled Nursing Facility. *N Engl J Med* 382(22):2081-2090.

Bostanci Ceran B, Ozates S (2020) Ocular manifestations of coronavirus disease 2019. *Graefes Arch Clin Exp Ophthalmol*. Jun 6:1-5. doi: 10.1007/s00417-020-04777-7. (Online ahead of print).

Cahill TJ, Cravatt B, Goldman LR, et al. (2020) Scientists to Stop COVID-19. Accessed May 1, 2020.
https://s.wsj.net/public/resources/documents/Scientists_to_Stop_COVID19_2020_04_23_FINAL.pdf

Casadevall A, Pirofski LA (2020) The convalescent sera option for containing COVID-19. *J Clin Invest* 130(4):1545-1548.

Chan JF, Yuan S, Kok KH, et al. (2020) A familial cluster of pneumonia associated with the 2019 novel coronavirus indicating person-to-person transmission: a study of a family cluster *Lancet*. 395(10223): 514-523.

Constantin C, Neagu M, Supeanu TD, Chiurciu V, Spandidos A (2020) IgY - turning the page toward passive immunization in COVID-19 infection (Review). *Exp Ther Med* 20(1):151-158.

Duan K, Liu B, Li C, Zhang H, Yu T, Qu J, et al. (2020) Effectiveness of convalescent plasma therapy in severe COVID-19 patients. *Proc Natl Acad Sci USA* 117(17):9490-9496.

Fu CY, Huang H, Wang XM, Liu YG, Wang ZG, Cui SJ, et al. (2006) Preparation and evaluation of anti-SARS coronavirus IgY from yolks of immunized SPF chickens. *J Virol Methods* 133(1): 112-115.

Giraudi V, Riganti C, Torales MR, Sédola H, Gaddi E (1997) Upper respiratory infections in children: Response to endonasal administration of IGA. *Int J Pediatr Otorhinolaryngol* 39(2): 103-110.

Gisondi P, PIaserico S, Bordin C, Alaibac M, Girolomoni G, Naldi L (2020) Cutaneous manifestations of SARS-CoV-2 infection: a clinical update. *J Eur Acad Dermatol Venereol* Jun 25. doi: 10.1111/jdv.16774. (Online ahead of print).

Guo-Zhu H, Xi-Ling Z, Zhu W, Li-Hua W, Dan H, Xiao-Mu W, et al. (2015) Therapeutic potential of combined anti-IL-1β IgY and anti-TNF-α IgY in guinea pigs with allergic rhinitis induced by ovalbumin. *Int Immunopharmacol* 25(1):155-161.

Hamming I, Timens W, Bulthuis ML, Lely AT, Navis G, van Goor H (2004) Tissue distribution of ACE2 protein, the functional receptor for SARS coronavirus. A first step in understanding SARS pathogenesis. *J Pathol* 203(2): 631-637.

He X, Lau EHY, Wu P, Deng X, Wang J, Hao X, et al. (2020) Temporal dynamics in viral shedding and transmissibility of COVID-19. *Nat Med* 26(5):672-675.

Heikkinen TA, Ruohola A, Ruuskanen O, Waris M, Uhari M, Hammarström L (1998) Intranasally administered immunoglobulin for the prevention of rhinitis in children. *Pediatr Infect Dis J* 17(5): 367-372.

Hemmingsson P, Hammarström L (1993) Nasal administration of immunoglobulin as effective prophylaxis against infections in elite cross-country skiers. *Scand J Infect Dis* 25(6):783-785.

Horie K, Horie N, Abdou AM, Yang J-O, Yun S-S, Chun H-N, et al. (2004) Suppressive effect of functional drinking yogurt containing specific egg yolk immunoglobulin on *Helicobacter pylori* in humans. *J Dairy Sci* 87(12): 4073-4079.

Ikemori Y, Ohta M, Umeda K, Icatlo FC Jr, Kuroki M, Yokoyama H, et al. (1997) Passive protection of neonatal calves against bovine coronavirus-induced diarrhea by administration of egg yolk or colostrum antibody powder. *Vet Microbiol* 58(2-4):105-111.

Jia HP, Look DC, Hickey M, Shi L, Pewe J, Netland M, et al. (2006) Infection of human airway epithelia by SARS coronavirus is associated with ACE2 expression and localization. *Adv Exp Med Biol* 581: 479-484.

Keller MA, Stiehm ER (2000) Passive immunity in prevention and treatment of infectious diseases. *Clin Microbiol Rev* 13(4):602-614.

Kimball A, Hatfield KM, Arons M, James A, Taylor J, Spicer K, et al; and Public Health – Seattle & King County; CDC COVID-19 Investigation Team (2020) Asymptomatic and Presymptomatic SARS-CoV-2 Infections in Residents of a Long-Term Care Skilled Nursing Facility - King County, Washington, March 2020. *MMWR Morb Mortal Wkly Rep* 69(13):377-381.

Kollberg H, Carlander D, Olesen H, Wejåker PE, Johannesson M, Larsson A (2003) Oral administration of specific yolk antibodies (IgY) may prevent Pseudomonas aeruginosa infections in patients with cystic fibrosis: a phase I feasibility study. *Pediatr Pulmonol* 35(6):433-40.

Kovacs-Nolan J, Mine Y (2012) Egg yolk antibodies for passive immunity. *Ann Rev Food Sci Technol* 3: 163-182.

Li Q, Guan X, Wu P, Wang X, Zhou L, Tong Y, et al. (2020) Early Transmission Dynamics in Wuhan, China, of Novel Coronavirus-Infected Pneumonia. *N Engl J Med* 382(13):1199-1207.

Long QX, Liu BZ, Deng HJ, Wu GC, Deng K, Chen YK, et al. (2020) Antibody responses to SARS-CoV-2 in patients with COVID-19. *Nat Med* 26(6): 845-848.

Mercante G, Ferreli F, De Virgilio A, et al. (2020) Prevalence of Taste and Smell Dysfunction in Coronavirus Disease 2019. *JAMA Otolaryngol Head Neck Surg* Jun 18. doi: 10.1001/jamaoto.2020.1155. (Online ahead of print)

Mizumoto K, Chowell G (2020) Transmission potential of the novel coronavirus (COVID-19) onboard the diamond Princess Cruises Ship, 2020. *Infect Dis Model* 5:264-270.

Nilsson E, Larsson A, Olesen HV, Wejåker PE, Kollberg H (2008) Good effect of IgY against Pseudomonas aeruginosa infections in cystic fibrosis patients. *Pediatr Pulmonol* 43(9):892-899.

Nguyen SV, Nguyen MTH, Tran BC, Ho MTQ, Umeda K, Rahman S (2018) Evaluation of lozenges containing egg yolk antibody against porphyromonas gingivalis gingipains as an adjunct to conventional non-surgical therapy in periodontitis patients: A randomized controlled clinical trial. *J Periodont* 89(11): 1334-1339.

Nguyen HH, Tumpey TM, Park HJ, Byun YH, Tran LD, Nguyen VD, et al. (2010) Prophylactic and therapeutic efficacy of avian antibodies against influenza virus H5N1 and H 1N1 in mice. *PLoS One* 5(4):e10152.

Ong SWX, Tan YK, Chia PY, Lee T, Ng O, Su M, et al. (2020) Air, Surface Environmental, and Personal Protective Equipment Contamination by Severe Acute Respiratory Syndrome Coronavirus 2 (SARS-CoV-2) From a Symptomatic Patient. *JAMA* 323(16):1610-1612.

Otterbeck A, Hanslin K, Lantz EL, Larsson A, Stålberg J, Lipcsey M (2019) Inhalation of specific anti-Pseudomonas aeruginosa IgY antibodies transiently decreases P. aeruginosa colonization of the airway in mechanically ventilated piglets. *Intensive Care Med Exp* 7(1):21. Erratum in: I*ntensive Care Med Exp*. 2019. 7(1):24.

Rahman S, Higo-Moriguchi K, Htun KW, Taniguchi K, Icatlo FC Jr, Tsuji T, et al. (2012) Randomized placebo-controlled clinical trial of immunoglobulin Y as adjunct to standard supportive therapy for rotavirus-associated diarrhea among pediatric patients. *Vaccine* 30(31):4661-4669.

Rahman S, Van Nguyen S, Icatlo FC Jr., Umeda K, Kodama Y (2013) Oral passive IgY-based immunotherapeutics: A novel solution for prevention and treatment of alimentary tract diseases. *Hum Vaccin Immunother* 9(5): 1039-1048.

Ranjbar M, Behrouz B, Norouzi F, Gargari S (2019) Anti-PcrV IgY Antibodies Protect Against Pseudomonas Aeruginosa Infection in Both Acute Pneumonia and Burn Wound Models. *Mol Immunol* 116:98-105.

Salazar E, Perez KK, Ashraf M, Chen J, Castillo B, Christensen PA, et al. (2020) Treatment of COVID-19 Patients with Convalescent Plasma. *Am J Pathol* 190:1680-1690.

Sarker SA, Casswall TH, Juneja LR, Hoq E, Hossain I, Fuchs GJ, et al. (2001) Randomized, placebo-controlled, clinical trial of hyperimmunized chicken egg yolk immunoglobulin in children with rotavirus. *J Pediatr Gastroenterol Nutr* 32(1):19-25.

Schuster A, Bend J, Høiby N, Verde PE, Rottmann A, Larsson A (2019) Clinical study to evaluate an anti-pseudomonas aeruginosa IgY gargling solution (EUDRACT 2011-000801-39). *J Cyst Fibros* 18, Suppl 1, S23 (abstract WS12-5).

Sethuraman N, Jeremiah SS, Ryo A (2020) Interpreting Diagnostic Tests for SARS-CoV-2. *JAMA*. May 6. doi: 10.1001/jama.2020.8259 (Epub ahead of print).

Shen C, Wang Z, Zhao F, Yang Y, Li J, Yuan J, et al. (2020) Treatment of 5 Critically Ill Patients With COVID-19 With Convalescent Plasma. *JAMA* 323(16):1582-1589.

Somasundaram R, Choraria A, Antonysamy M (2020) An Approach Towards Development of Monoclonal IgY Antibodies Against SARS CoV-2 Spike Protein (S) Using Phage Display Method: A Review. *Int Immunopharmacol* June 3. 85:106654 [Online ahead of print].

Sungnak W, Huang N, Bécavin C, Berg M, Queen R, Litvinukova M, et al. and HCA Lung Biological Network (2020) SARS-CoV-2 entry factors are highly expressed in nasal epithelial cells together with innate immune genes. *Nat Med* 26 (5): 681-687.

Takeuchi S, Motohashi J, Kimori H, Nakagawa Y, Tsurumoto A (2016) Effects of oral moisturising gel containing egg yolk antibodies against Candida albicans in older people. *Gerodontology* 33(1): 128-134.

Vega C, Bok M, Chacana P, Saif L, Fernandez F, Parreno V (2011) Egg yolk IgY: protection against rotavirus induced diarrhea and modulatory effect on the systemic and mucosal antibody responses in newborn calves. *Vet Immunol Immunopathol* 142(3-4): 156-169.

Wallach MG, Webby RJ, Islam F, Walkden-Brown S, Emmoth E, Feinstein R. (2011) Cross-protection of chicken immunoglobulin Y antibodies against H5N1 and H1N1 viruses passively administered in mice. *Clin Vaccine Immunol* 18(7): 1083-1090.

Warr GW, Magor KE, Higgins DA (1995) IgY: Clues to the origins of modern antibodies. *Immunol Today* 16(8): 392-398.

Wei-Xu H, Wen-Yun Z, Xi-Ling Z, Zhu W, Li-Hua W, Xiao-Mu W, et al. (2016) Anti-interleukin-1 beta/tumor necrosis factor-alpha IgY antibodies reduce pathological allergic responses in guinea pigs with allergic rhinitis. *Mediators Inflamm* 2016:3128182.

Weltzin R, Monath TP (1999) Intranasal antibody prophylaxis for protection against viral disease. *Clin Microbiol Rev* 12(3): 383-393.

Wölfel R, Corman VM, Guggemos W, et al (2020). Virological assessment of hospitalized patients with COVID-2019. *Nature* 581(7809):465-469.

World Health Organization (2020) Coronavirus Disease (COVID-19) Pandemic. Accessed August 3, 2020. https://www.who.int/emergencies/diseases/novel-coronavirus-2019.

Yang YE, Wen J, Zhao S, Zhang K, Zhou Y (2014) Prophylaxis and therapy of pandemic H1N1 virus infection using egg yolk antibody. *J Virol Methods* 206: 19-26.

Yokoyama K, Sugano N, Shimada T, Shofiqur RA, Ibrahim el-SM, Isoda R, et al. (2007) Effects of egg yolk antibody against Porphyromonas gingivalis gingipains in periodontitis patients. *J Oral Sci* 49(3): 201-206.

Zhang X, Calvert RA, Sutton BJ, Doré KA (2017) IgY: A key isotype in antibody evolution. *Biol Rev Camb Philos Soc* 92(4): 2144-2156.

Zhou P, Yang XL, Wang XG, Hu B, Zhang L, Zhang W, et al. (2020). A pneumonia outbreak associated with a new coronavirus of bat origin. *Nature* 579(7798):270-273.

Appendix A:
Sponsor Protocol Approval

**Protocol Title:**	A Phase 1 Study in Healthy Participants to Evaluate the Safety, Tolerability, and Pharmacokinetics of Single-Ascending and Multiple Doses of an Anti-Severe Acute Respiratory Syndrome Coronavirus 2 (SARS-CoV-2) Chicken Egg Antibody (IgY)
**Protocol Number:**	CVR001

This study will be conducted in compliance with the clinical study protocol (and amendments), International Council for Harmonisation of Technical Requirements for Pharmaceuticals for Human Use, Good Clinical Practice and applicable regulatory requirements

This study protocol has been approved by the following persons:

Signed: ______________________________ Date: ________________________________

Daria Mochly-Rosen, PhD
Founder and Co-Director
SPARK at Stanford

Investigator Protocol Signature Page

Protocol Title: A Phase 1 Study in Healthy Participants to Evaluate the Safety, Tolerability, and Pharmacokinetics of Single-Ascending and Multiple Doses of an Anti-Severe Acute Respiratory Syndrome Coronavirus 2 (SARS-CoV-2) Chicken Egg Antibody (IgY)

Protocol Number: CVR001

By signing this protocol, the investigator agrees to conduct the study in accordance with the protocol, generally accepted standards of Good Clinical Practice, and all applicable federal, state, and local laws, rules, and regulations relating to the conduct of the clinical study. In addition, the investigator agrees to provide the sponsor with accurate financial information to allow the sponsor to submit complete and accurate certification and disclosure statements as required by federal regulations.

By signing this protocol, the investigator agrees to be responsible for implementing and maintaining quality control and quality assurance systems with written procedures to ensure that the trials are conducted and data are generated, documented, and reported in compliance with the protocol, accepted standards of Good Clinical Practice, and all applicable federal, state, and local laws, rules, and regulations relating to the conduct of the study.

Investigator's Signature	Print Name	Date

Site Address and Telephone
